# Monitoring blood-flow in the mouse cochlea using an endoscopic laser speckle contrast imaging system

**DOI:** 10.1371/journal.pone.0191978

**Published:** 2018-02-28

**Authors:** Tae Hoon Kong, Sunkon Yu, Byungjo Jung, Jin Sil Choi, Young Joon Seo

**Affiliations:** 1 Department of Otorhinolaryngology-Head and Neck Surgery, Yonsei University Wonju College of Medicine, Wonju, South Korea; 2 Department of Biomedical Engineering, Yonsei University College of Health Science, Wonju, South Korea; Hokkaido Daigaku, JAPAN

## Abstract

Laser speckle contrast imaging (LSCI) enables continuous high-resolution assessment of microcirculation in real-time. We applied an endoscope to LSCI to measure cochlear blood-flow in an ischemia–reperfusion mouse model. We also explored whether using xenon light in combination with LSCI facilitates visualization of anatomical position. Based on a previous preliminary study, the appropriate wavelength for penetrating the thin bony cochlea was 830 nm. A 2.7-mm-diameter endoscope was used, as appropriate for the size of the mouse cochlea. Our endoscopic LSCI system was used to illuminate the right cochlea after dissection of the mouse. We observed changes in the speckle signals when we applied the endoscopic LSCI system to the ischemia-reperfusion mouse model. The anatomical structure of the mouse cochlea and surrounding structures were clearly visible using the xenon light. The speckle signal of the cochlea was scattered, with an intensity that varied between that of the stapes (with the lowest signal), the negative control, and the stapedial artery (with the highest signal), the positive control. In the cochlear ischemia–reperfusion mouse model, the speckle signal of the cochlea decreased during the ischemic phase, and increased during the reperfusion phase, clearly reflecting cochlear blood-flow. The endoscopic LSCI system generates high-resolution images in real-time, allowing visualization of blood-flow and its changes in the mouse cochlea. Anatomical structures were clearly matched using LSCI along with visible light.

## Introduction

Despite years of investigation, the causes of several inner ear disorders, including sudden sensorineural hearing loss, noise-induced hearing loss, Meniere’s disease, and presbycusis, remain unclear [1]. Viral infection, autoimmune disease, and impaired blood-flow are suggested as possible causes [2–4]. There are several studies on inner ear disorders and impaired cochlear blood-flow in animal models [5]; decreased cochlear blood-flow was observed in a hearing-impaired mouse, in response to noise, etc., and the consequences of hearing loss were investigated after mice had been subjected to blood-flow manipulation or hypoxia [6–9]. Therefore, measuring blood-flow in the cochlea is important. Nevertheless, direct measurement of cochlear blood-flow is difficult and techniques for assessing blood-flow are still under development [5]. Numerous studies have been conducted to evaluate methods for measuring cochlear blood-flow, including the traditional histopathological method, contrast-enhanced magnetic resonance imaging (MRI), laser-Doppler flowmetry (LDF), and the microsphere method [10,11]. However, visualization of cochlear blood-flow is limited; contrast-enhanced MRI is invasive, and poses risks associated with the contrast agent disrupting the physiology of the cochlea [6], and LDF reflects blood-flow within a large volume of the cochlea, which is surrounded by thin bony structures, and it is, therefore, difficult to ascertain precisely which anatomical structures contribute to the measured signal [11]. Moreover, given that it cannot reflect real-time blood-flow, the microsphere method requires labeled microspheres to be injected into the bloodstream, animals to be sacrificed, and the cochlea to be removed [11].

Laser speckle-contrast imaging (LSCI) is widely used for imaging microvascular blood-flow [12]. According to previous studies, LSCI is also an appropriate modality for assessing microvascular reperfusion [13]. Moreover, LSCI is non-invasive, and does not require contact with the tissue. Illumination of the tissue surface with laser light gives rise to a phenomenon called “laser speckle,” where scattered light creates an interference effect due to the irregularities of the structure [14]. Depending on the functional level of the image-acquisition camera, LSCI can provide high spatiotemporal resolution imaging [12]. Thus, LSCI can provide high resolution, continuous, real-time assessment of microcirculation. Currently, LSCI is widely used in the ophthalmologic field to measure retinal blood-flow [15–18]. A few studies have used LSCI to visualize the cochlea [19,20]; however, they did not generate specific anatomical information through laser speckle imaging, the image reproducibility was low when a third observer was involved (i.e. operator-dependent), and they did not present high resolution imaging because the cochlea is hidden deep in the temporal bone.

Therefore, to overcome these limitations, we used an endoscope to deliver the laser light close to the deeply located cochlea and obtain high-resolution images; we also used xenon light to identify anatomical structures in mouse cochlea that are difficult to distinguish via laser light only. We also investigated changes in mouse cochlear blood-flow via endoscopic LSCI in our ischemia–reperfusion mouse models.

## Methods

### System setup

**Fig 1:**
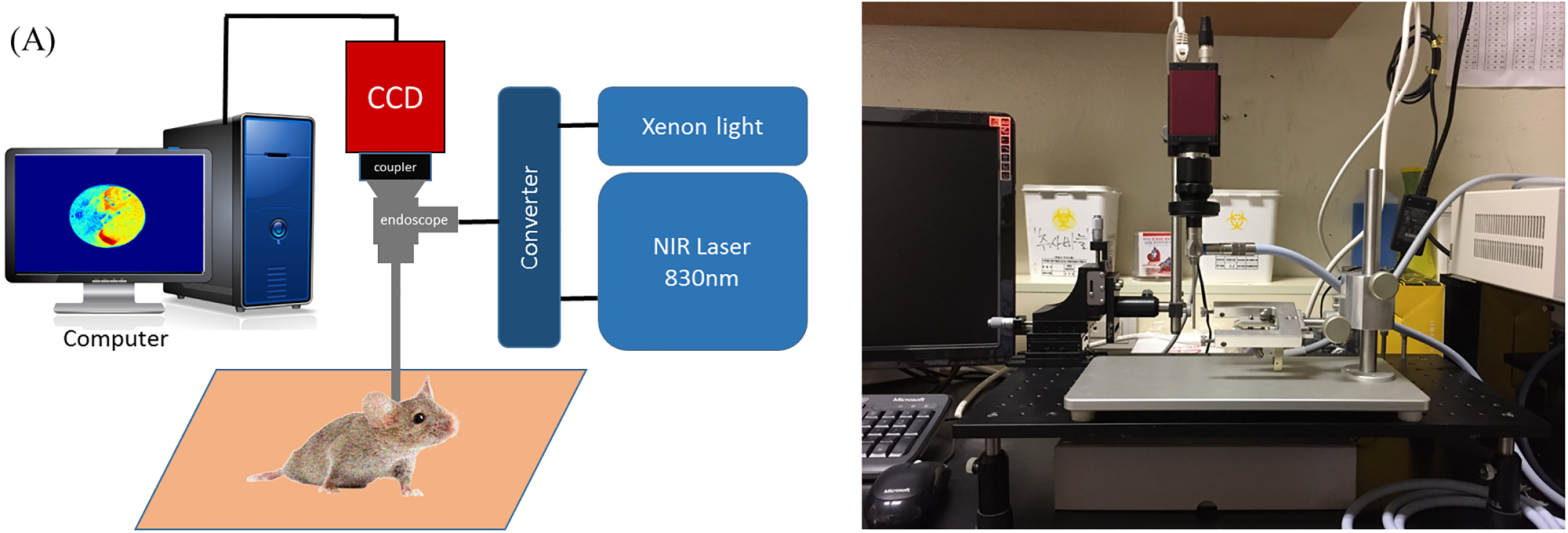
Endoscopic laser speckle imaging system setup. (A) A schematic of our system. (B) Real system. CCD = charge-coupled device; NIR = near infrared.

The endoscopic LSCI system is presented in Fig 1.

The system consists of an image processing computer, acquisition camera, and light source containing a laser diode and xenon light (Xenon Nova 330, Karl Storz Endoscope, Tuttlingen, Germany). The cochlear blood vessel is surrounded by thin, bony cochlea that could potentially interfere with laser transmission. As described by Professor Jung, from the Laboratory of Biomedical Optics in our institute in a previous study to determine the appropriate wavelength for sufficient permeability to penetrate the bony cochlea, the laser diode emitted a near infrared (NIR) wavelength of λ = 830 nm. However, as the laser light source showed a black and white speckle pattern in the raw laser speckle image, a visible-light-wavelength-light-source was additionally required to clearly observe the anatomical structure of the cochlea; thus, a xenon light was used. We set a converter between the xenon light and laser diode to allow us to collect anatomical images and LSCI simultaneously, without moving the animal.

An endoscope with a diameter of 2.7 mm was used, given the size of the mice cochlear (0°, 2.7-mm diameter, 60-mm length, Mega Medical, Seoul, South Korea). By using an endoscope, it was possible to observe the cochlea enlarge with the high-resolution display. Moreover, despite disturbance to the structures around the cochlea deep in the temporal bone, it was possible to observe the cochlea more specifically by moving the end of endoscope close to the cochlea. The reflected light was collected by an NIR charge-coupled device camera (Manta G-145B NIR, Allied Vision Technology, Haaksbergen, Netherlands) connected to the endoscope using an endoscope zoom coupler (18–35 mm, LEOA1835, LenOpTec, Fuzhou, China). The final laser output power was 1.4 mW, within the maximum permissible exposure. In summary, an NIR laser wavelength of 830 nm was emitted from a light source, through a light cable and endoscope, to the mouse cochlea. The reflected laser signals from the mouse cochlea passed through the endoscope to the charge-coupled device (CCD). Each laser signal obtained from the CCD was implemented as a contrast image through a series of computer algorithms. All systems were set and their stability verified by Professor Jung’s group at the Laboratory of Biomedical Optics of Department of Biomedical Engineering, Yonsei University, Wonju, South Korea.

### Animal preparation

Mice (C57BL/6J) aged 8 weeks were used in our study. All procedures were performed with the approval of the Institutional Animal Care and Use Committee at the Yonsei University Wonju College of Medicine (Protocol YWC-160826-1).

**Fig 2:**
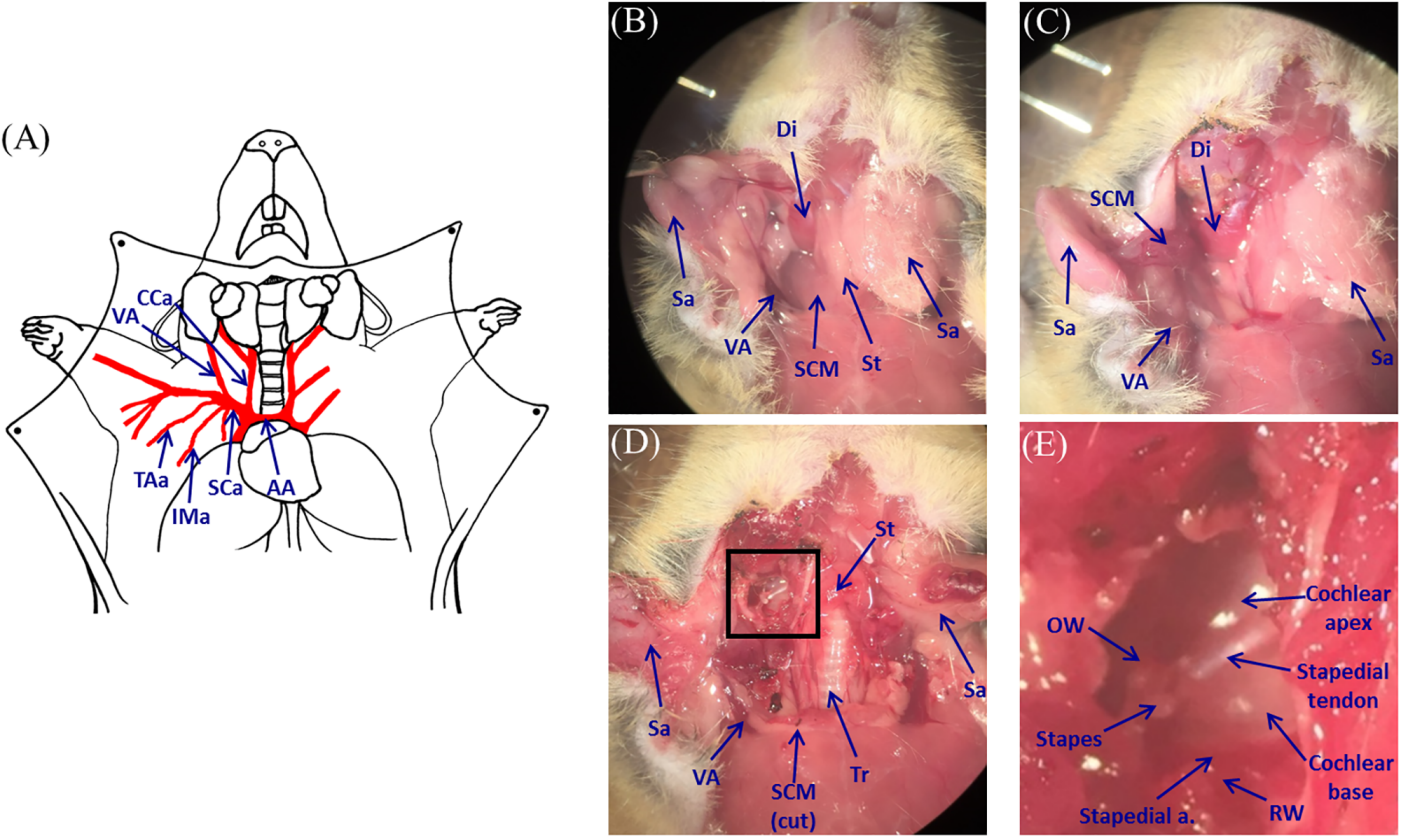
Mouse dissection and exposure of mouse cochlea. (A) Diagram of mouse neck anatomy. (B) After making an incision along the mid-line of the neck, salivary glands were lateralized. (C) After cutting the sternocleidomastoid muscle on the proximal portion and anterior belly of the digastric muscle. (D) The cochlea was exposed after identifying and removing the bulla. (E) Magnified image of the cochlea, from the square in (D). Cca = common carotid artery; VA = vertebral artery; AA = aortic arch; SCa = subclavian artery; IMa = internal mammary artery; TAa = thoraco-acromial artery; Sa = salivary gland; Di = digastric muscle; St = strap muscle; SCM = sternocleidomastoid muscle; Tr = trachea; OW = oval window; RW = round window.

Five mice were anesthetized using a mixture of 100 mg/kg of ketamine and 10 mg/kg of xylazine, administered intraperitoneally. The head of the mouse was fixed onto a platform to exclude motion artifacts. We performed dissection of the neck and exposed the right cochlea via a ventral surgical approach, after slight modification of the previously described method [21,22]. An incision was made to the midline of the neck, and the salivary glands were dissected and lateralized (Fig 2B).

We successfully located the sternocleidomastoid muscle (SCM), digastric muscle, strap muscles, vertebral artery (VA), common carotid artery, and other cervical structures. After the right SCM was cut proximally, and the right digastric muscle was cut at the intermediate tendon, both muscles were lateralized to obtain a wider visual field (Fig 2C). Thereafter, we were able to locate additional cervical structures, such as the hypoglossal and facial nerves. To ensure minimal disturbance to the circulatory system, major vessels, including the jugular vein, were not ligated. After dissection of the cervical structures, we marked both the vertebral arteries for later clamping and the bulla was identified using the cut SCM, hypoglossal nerve, and digastric muscle as landmarks. After removing the bulla, the cochlea and surrounding anatomical structures were exposed (Fig 2D). As observed in a magnified view of the cochlea, we could observe specific anatomical structures around the cochlea (Fig 2E). During the dissection, only limited soft cotton pledgets and electric cauterization were used to control bleeding from the capillaries.

### Obtaining speckle contrast images

A speckle contrast image (K) is defined as the ratio of the standard deviation (*σ*) to the mean intensity (〈*I*〉) in a small lesion of the raw speckle image (Eq 1). Raw speckle data were converted to speckle contrast images in real-time using an optimized algorithm. Our system calculated speckle contrast images at a rate of 8 frames per second and showed the image on the monitor. The speckle images were simultaneously saved. Each set of 30 speckle contrast images was averaged to reduce image-to-image variations.

$$\text{K}=\frac{\sigma }{<I>}$$ (1)

### Study protocol

**Fig 3:**
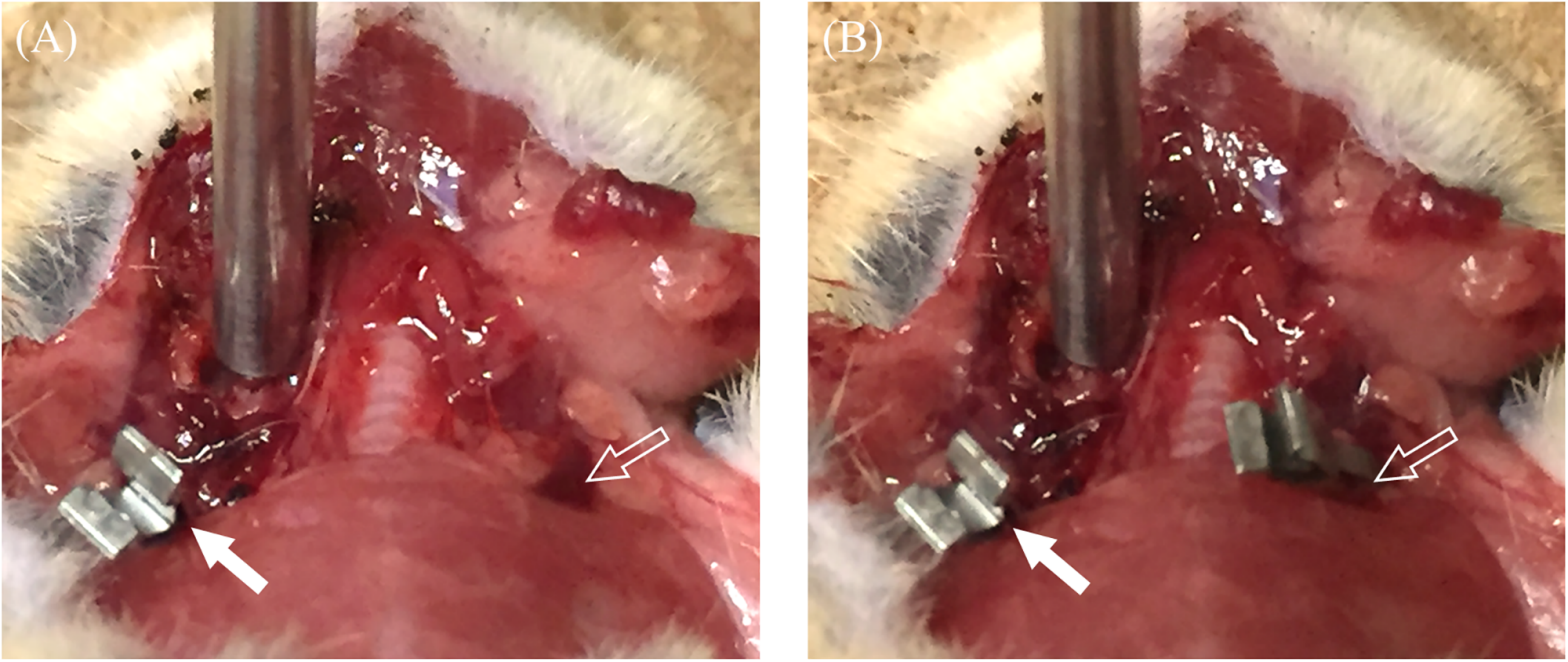
Clamping of the vertebral artery. (A) Single clamping of the ipsilateral side of the vertebral artery. (B) Additional clamping of the contralateral side of the vertebral artery. (white-filled arrow: clamping of the right vertebral artery; outline arrow: left vertebral artery).

After dissection, the mouse was placed on a stereotaxic apparatus to avoid motion. First, our endoscopic LSCI system illumined the mouse cochlea using xenon light, to identify the anatomy of the cochlea and properly position the endoscope. The end of the endoscope was placed as close to the cochlea as possible, without making contact. Next, the light source was changed to NIR laser (λ = 830 nm), and our endoscopic LSCI system was used to measure the image for approximately 5 seconds. We also generated a mouse cochlear ischemia-reperfusion model, and used the endoscopic LSCI system to evaluate cochlear blood-flow in this model. To generate minimal ischemia in the right cochlea, we clamped the ipsilateral VA using a microvascular clamp (Acland^®^ clamp, S&T, Neuhausen am Rheinfall, Switzerland) without moving the mouse (Fig 3A).

After stabilizing the speckle signal, the images were recorded for 5 seconds. We then performed additional clamping of the contralateral VA (Fig 3B). After signal stabilization, the image was acquired for 5 seconds. Finally, we simultaneously released the clamps on both VAs, and again recorded images for 5 seconds using the endoscopic LSCI system after speckle-signal stabilization. This reperfusion of the cochlea was performed after about 20 seconds of total ischemic time.

### Comparison between speckle images and statistical analysis

To assess the ischemic effect of the cochlea, we used the averaged images (from the 30 speckle contrast images) to compare each step of manipulation of the VAs. For each averaged speckle contrast image, we obtained raw data for the following regions of interest (ROIs): 1) stapes, 2) base of the cochlea, 3) apex of the cochlea. Statistical analyses of ROI values were performed using the Statistical Package for Social Sciences Software (SPSS 22.0 for Windows, SPSS Inc., Chicago, IL, USA). We performed analyses of variance to compare the speckle-signal conditions of each mouse, and Kruskal-Wallis tests to compare the speckle-signal conditions between mice. Differences were considered statistically significant when *p* values were less than 0.05.

## Results

### Visualization of cochlea using endoscopic laser speckle contrast imaging

**Fig 4:**
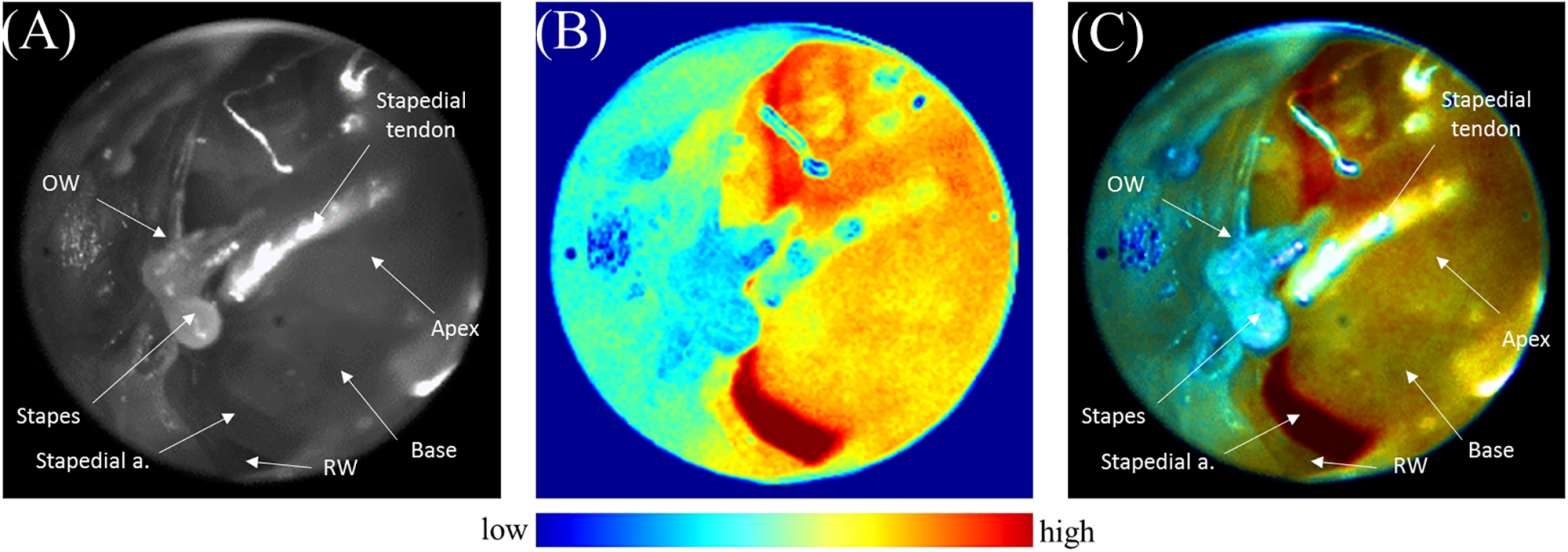
Visualization of the cochlea using endoscopic laser speckle. (A) Visualization of the cochlea with a xenon light source. (B) Visualization of the cochlea with a laser speckle light source. (C) Merged image using both xenon and laser speckle imaging.

Xenon light in our endoscopic LSCI system allowed good visualization of the anatomy of the mouse cochlea and surrounding structures, including the stapes, stapedial tendon, stapedial artery, round window, etc. (Fig 4A).

After we switched from xenon to NIR laser light, we could identify blood-flow in the cochlea in the speckle contrast image. Using the averaged speckle contrast image, we could observe blood-flow in the cochlea more clearly (Fig 4B). The stapedial artery was visualized clearly, showing the highest speckle signal in our system, whilst the stapes (consisting of bone) showed the lowest speckle signal. We used the stapedial artery as a positive control for the speckle images, which verified that the endoscopic LSCI system was operating appropriately; conversely, the speckle signals from the stapes were used as a negative control. The microvasculature of the cochlea is covered by thin bony cochlea; thus, the speckle signal is represented by scattered signals between the stapes and stapedial artery.

We fused the xenon light image and averaged speckle image, modifying the opacity of the images, to compare anatomy and blood-flow (Fig 4C). Anatomical structures, including the stapes, stapedial tendon and artery, round window, and cochlea, were well correlated between the xenon light and averaged speckle images.

### Blood-flow changes in a cochlear ischemia-re-perfusion mouse model using endoscopic laser speckle contrast imaging

**Fig 5:**
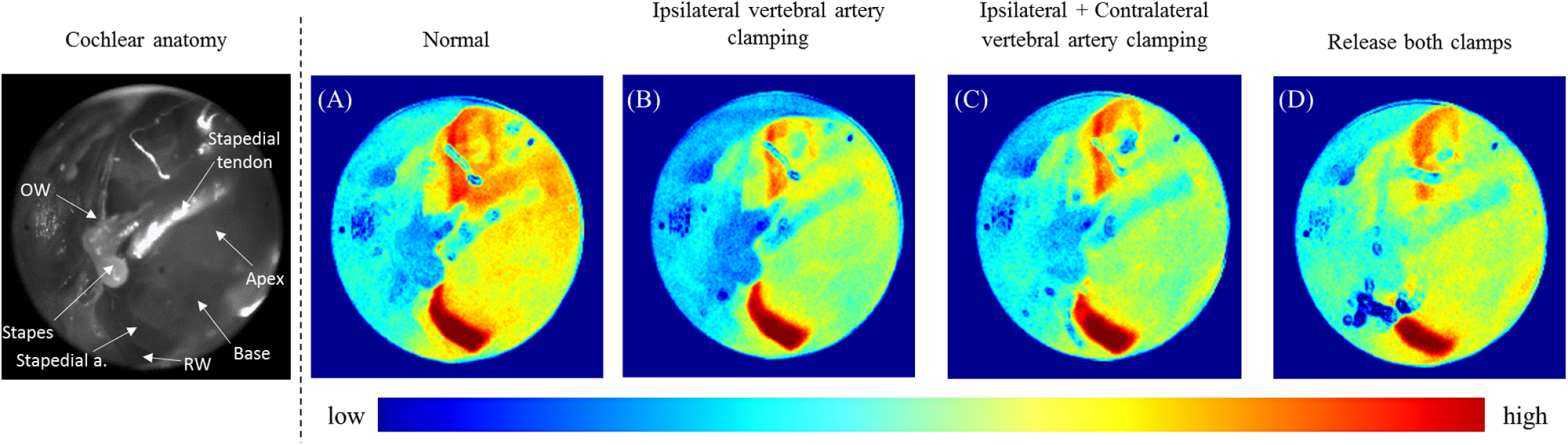
Averaged laser speckle contrast imaging using 30 speckle contrast images at baseline and after ipsilateral vertebral artery clipping, additional contralateral vertebral artery clipping, and releasing both clamps.

In our cochlear ischemia–reperfusion mouse model (generated by manipulation of VAs as described above) we obtained speckle contrast images. As it was difficult to identify the difference between each naïve laser speckle image manipulation, we used averaged images to identify the differences. Fig 5 shows the averaged speckle images from each manipulation, including with control, ipsilateral VA clamping, additional contralateral VA clamping, and both clamps released.

Averaged images were produced from 30 naïve speckle contrast images. Considering the anatomy of the cochlea and surrounding structures, the lowest speckle signal was found in the stapes and the highest speckle signal in the stapedial artery. The speckle signals from the cochlea varied between these two values (Fig 5A). After clamping the ipsilateral VA, a relative decrease in the speckle signal of the cochlea was observed, indicating a reduction in blood-flow. However, the lowest signal of the stapes and the highest signal of stapedial artery remained unchanged (Fig 5B). After additional clamping of the contralateral VA, the speckle signal of the cochlea was slightly reduced; however, once again, the lowest and highest signals (of the stapes and stapedial artery, respectively) remained unchanged (Fig 5C). When both the ipsilateral and contralateral VAs were unclamped simultaneously, the speckle signals of the cochlea were increased, but remained less than in the control (Fig 5D). Although it is not easy to distinguish, via the naked eye, we observed similar findings in a consecutive view of representative un-averaged naïve speckle images (S1 Fig).

### Comparison of speckle signal values for cochlea regions of interest

To quantify the differences between each laser speckle contrast image, we collected and analyzed raw speckle data at each manipulation of the mouse at each ROI—1) stapes, 2) base of the cochlea, 3) apex of the cochlea. To standardize values, we used the cochlear base–stapes (base/stapes) and cochlear apex–stapes (apex/stapes) ratios of speckle signals. Tables 1 and 2 show the mean values of base/stapes and apex/stapes.

**Table 1:** Comparison between apex/stapes ratios of regions of interest in each mouse and condition. (VA: Vertebral artery).

	Normal	Ipsilateral VA clamping	Ipsilateral + Contralateral Va clamping	Release	*p*-value
**Mouse 1**	2.38±0.63	2.04±0.57	1.77±0.35	2.18±0.17	<0.001
**Mouse 2**	2.36±0.62	2.06±0.58	1.80±0.37	2.16±0.18	<0.001
**Mouse 3**	2.32±0.59	2.08±0.57	1.80±0.36	2.15±0.19	<0.001
**Mouse 4**	2.35±0.64	2.08±0.56	1.79±0.35	2.16±0.18	<0.001
**Mouse 5**	2.38±0.58	2.08±0.64	1.79±0.35	2.17±0.18	<0.001

**Table 2:** Comparison between base/stapes ratios of regions of interest in each mouse and condition. (VA: Vertebral artery).

	Normal	Ipsilateral VA clamping	Ipsilateral + Contralateral Va clamping	Release	*p*-value
**Mouse 1**	2.68±0.79	2.23±0.56	2.05±0.33	2.40±0.22	<0.001
**Mouse 2**	2.66±0.78	2.25±0.55	2.08±0.37	2.39±0.21	<0.001
**Mouse 3**	2.63±0.74	2.27±0.57	2.07±0.36	2.38±0.20	<0.001
**Mouse 4**	2.68±0.79	2.29±0.56	2.07±0.21	2.38±0.21	<0.001
**Mouse 5**	2.69±0.74	2.29±0.65	2.06±0.36	2.39±0.22	<0.001

**Fig 6:**
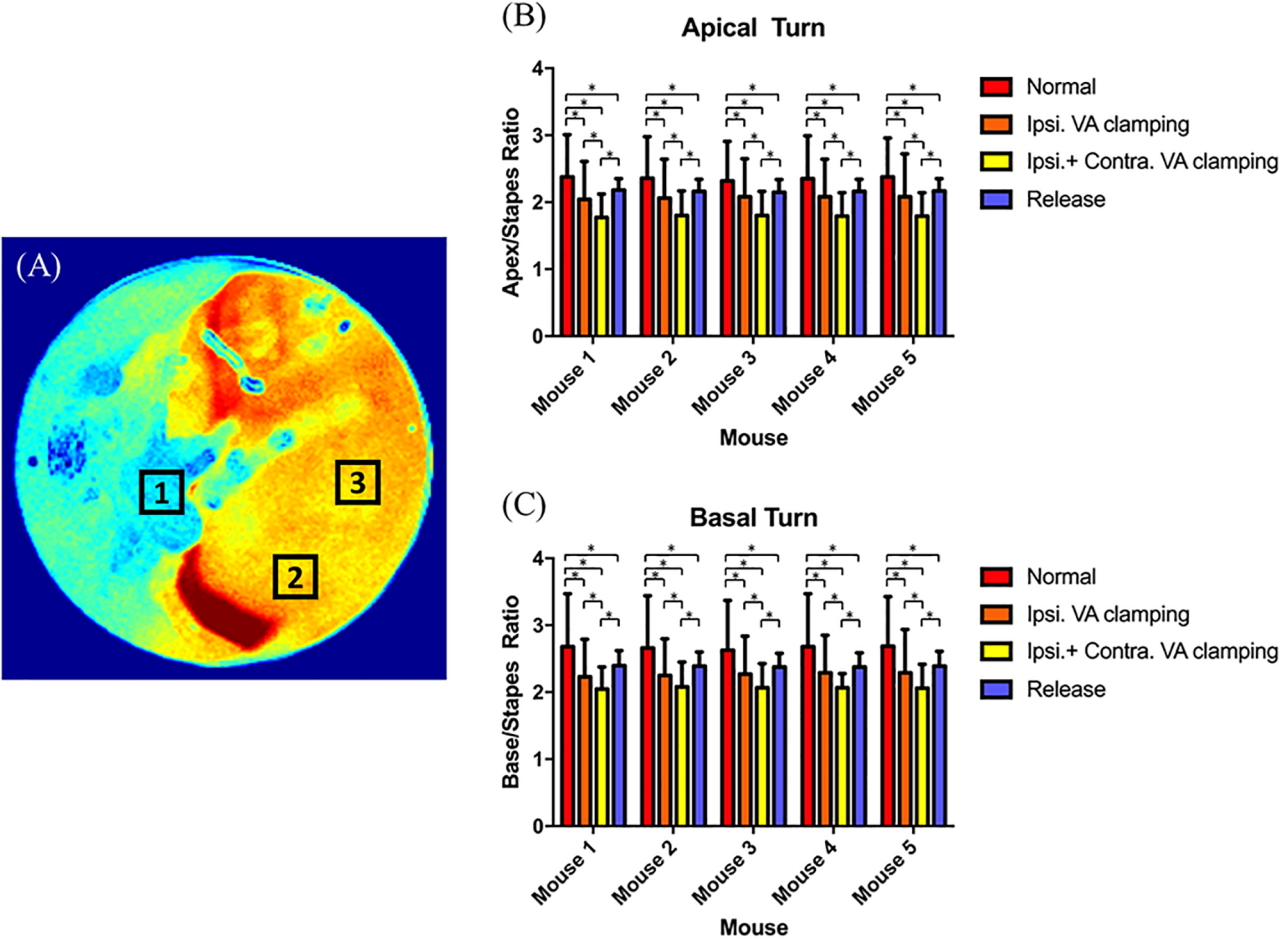
Comparison between speckle signaling values in regions of interest under each condition in each mouse. (A) Selecting the regions of interest (ROIs): 1) stapes; 2) base of the cochlea; 3) apex of the cochlea. (B) Comparison between apex/stapes ratio in each mouse. (C) Comparison between base/stapes ratio in each mouse. Ipsi. = ipsilateral; VA = vertebral artery; Contra. = contralateral.

There were statistically significant differences between the baseline states, after ipsilateral VA clamping, bilateral VA clamping, and subsequent unclamping, of all mice. However, within the same manipulation status, there were no significant differences between the base/stapes and apex/stapes across mice. These findings are indicative of significant differences between states within individuals, but not between individuals within states. Fig 6 shows that post-hoc analyses revealed statistically significant differences between groups, except between ipsilateral VA clamping and all unclamping in base/stapes and apex/stapes of all mouse.

## Discussion

The evidence from previous studies using animal models suggests that insufficient cochlear blood-flow is one of the mechanisms underlying inner ear disorders [23–25]. Therefore, direct measurement of blood-flow in the cochlea, particularly in real-time, could provide important information on the pathophysiology and treatments of various inner ear diseases in the cochlea. The LSCI technique provides high-resolution images in real-time, and does not require any invasive procedures [12]. Lasers used in LSCI are harmless to the human body, even if irradiated directly into the eye in healthy subjects; thus, the target area must simply be exposed for observation [14,26]. The final laser output power was 1.4 mW, within the maximum permissible exposure. However, LSCI remains limited as it cannot measure absolute blood-flow units (e.g. mL/s); rather, the results are expressed as flux, measured in arbitrary laser speckle perfusion units, and thus images may appear differently, depending on the range set for the arbitrary units [14].

The structure of the inner ear is very small and difficult to observe, as it is a complex structure located deep inside the temporal bone. The use of endoscopes can overcome these limitations, as they provide high resolution and wide fields of view within narrow physical ranges [27]. Given these advantages, trends in otological surgery have recently shifted, from microscopic to endoscopic surgery [28]. The cochlea in mice is small and has 1.75 turns, including the apical and basal turns [29,30]. We chose to use a 2.7-mm-diameter endoscope. Our system, the endoscopic LSCI system, provides a wide visual field with high-resolution images that allow observation of blood-flow in the cochlea and surrounding structures, despite the narrow space and small size of the cochlea.

By using a specific NIR-laser wavelength, the LSCI can detect blood flow within a certain depth. Davis et al reported the relationship between depth-dependence and degree of multiple scattering using LSCI with a wide range of visible and NIR wavelengths [31]. Based on a preliminary study using a phantom model, the appropriate wavelength (λ) to penetrate the bony mouse cochlea and detect blood-flow was calculated to be 830 nm. We could not observe the contour of the cochlear microvasculature as a speckle image, but could observe it as scattered signals thought to be generated when the speckle signal penetrated the bony cochlea whilst being reflected from the cochlea.

In our study, we used a combination of xenon and laser light. This was very helpful for identifying anatomical structures that are difficult to distinguish via laser light only. In our endoscopic LSCI system, the xenon light image was observed in grayscale and was sufficient for capturing the anatomical structures. There was no color interference when the xenon light image and LSCI scan were fused, because LSCI scans do not consist of black and white colors. The two images were fused as a separate action after the experiment; during the experiment, when the laser speckle image was observed after confirming the anatomical structure with the xenon image, the signal in the speckle image clearly reflected the anatomical structures.

During generation of the cochlear ischemia–reperfusion mouse model, when we clamped the VA on the ipsilateral side, the speckle signals of the cochlea decreased. When we additionally clamped the contralateral VA, the speckle signals of the cochlea decreased further; however, the speckle signal was not totally absent, indicating the presence of residual flow. Furthermore, the speckle signals from the stapedial artery remained unchanged during manipulation of the VAs. The cochlea is supplied principally from the inner ear artery (labyrinthine artery), which is a branch of the vertebrobasilar system [11], and the stapedial artery originates from the internal carotid artery [32]. By clamping the VAs, we effectively blocked the blood-flow of the vertebrobasilar system, but not the vessels originating from the carotid arteries. There have been reports that blood vessels, other than those of the vertebrobasilar system, may affect blood-flow in the cochlea and surrounding bone due to residual flow observed by the LDF and microsphere methods [32]. Therefore, residual speckle imaging signals, after clamping both the ipsilateral and contralateral VAs in our study, are consistent with reports from previous studies.

To obtain a reperfusion effect in the mouse cochlea, we released the clamps of both VAs after approximately 20 seconds of ischemia; the speckle signal was increased, but did not reach the same levels as before clamping. The signals, when both clamps were released, were not significantly different from the signals when ipsilateral clamping was performed. This suggests that the speckle signal increased due to recovery of cochlear blood-flow by unclamping, but also that blood-flow in the mouse cochlea did not increase sufficiently. Various experiments have been performed on several mammalian models to observe the effects of cochlear ischemia [11,33–38]. The stria vascularis and spiral ligament, which are supplied by the spiral modiolar artery, organ of Corti, and spiral ganglion, are strikingly vulnerable to occlusion of the vertebrobasilar system [39]. In the organ of Corti, the inner hair cells are more vulnerable to ischemia than the outer hair cells [39,40]. As the stria vascularis plays a crucial role in maintaining endocochlear potential, ion transport, and endolymphatic fluid balance essential for ear sensitivity, a cochlea with ischemic injury may lead to hearing loss [41–43]. Ren et al [36] reported that hearing function is changed according to clamping and release of the labyrinthine artery in the ischemia-reperfusion model of gerbil cochlea. In their study, cochlear blood-flow gradually decreased over time, only after a few seconds of clamping [36]. In our study, during the 20 seconds of ischemic time, ischemic injury of the cochlea may have occurred. Speckle signals that did not increase sufficiently after reperfusion may reflect ischemic injury of the mouse cochlea; such injury may be confirmed by audiometry or histopathological methods, but these were not carried out in the present study. We also only observed changes in blood-flow and did not match the hearing function of the mice after each manipulation of the VAs. Additional studies are required to confirm whether changes in blood-flow can be observed with LSCI in animal models with reduced hearing function.

The 830-nm infrared wavelength, which is highly efficient in penetrating the cochlear bone, was used in this study. However, owing to its scattering characteristics, we only observed the overall flow tendency in the cochlea and surrounding structures, not the microscopic structure of the cochlear blood vessels.

## Conclusion

Laser speckle contrast imaging was used to visualize and detect blood-flow changes in the mouse cochlea. Using an endoscope, LSCI provides wide-visual-field and high-resolution images. By combining xenon with laser light, we could identify the correct anatomical structures and found that these were well-correlated with speckle images. Blood-flow changes in the mouse cochlea, arising from clamping of VAs, were evident through LSCI, and may cause ischemic injury of the cochlea. However, additional LSCI studies in models with reduced cochlear function due to ischemic injury are required to confirm our findings.

## Supporting information

S1 Fig Serial view of representative laser speckle contrast images at normal, after ipsilateral vertebral artery(Va) clipping, after additional contralateral Va clipping and after releasing both clampings. (TIF)

S1 File Animal Research: Reporting In Vivo Experiments (ARRIVE) guidelines, developed by the national centre for the replacement, refinement & reduction of animals in research. All items in checklist of ARRIVE guideline were met in our study, confirmed by Young Joon Seo, the corresponding author.
(PDF)

S2 File The raw speckle data collected from each manipulation of each mouse. To standardize values, we used the cochlear base–stapes (base/stapes) and cochlear apex–stapes (apex/stapes) ratios of speckle signals. Each mouse follows the number in the table. The number of status is as follows. Status 1: Normal, Status 2: Ipsilateral vertebral artery clamping, Status 3: Bilateral vertebral artery clamping, Status 4: Both release.
(PDF)
