# Supplementary figures and images for: Monitoring blood-flow in the mouse cochlea using an endoscopic laser speckle contrast imaging system
Source: PLoS One. 2018 Feb 28;13(2):e0191978. doi: 10.1371/journal.pone.0191978 (PMC5830291; doi:10.1371/journal.pone.0191978)

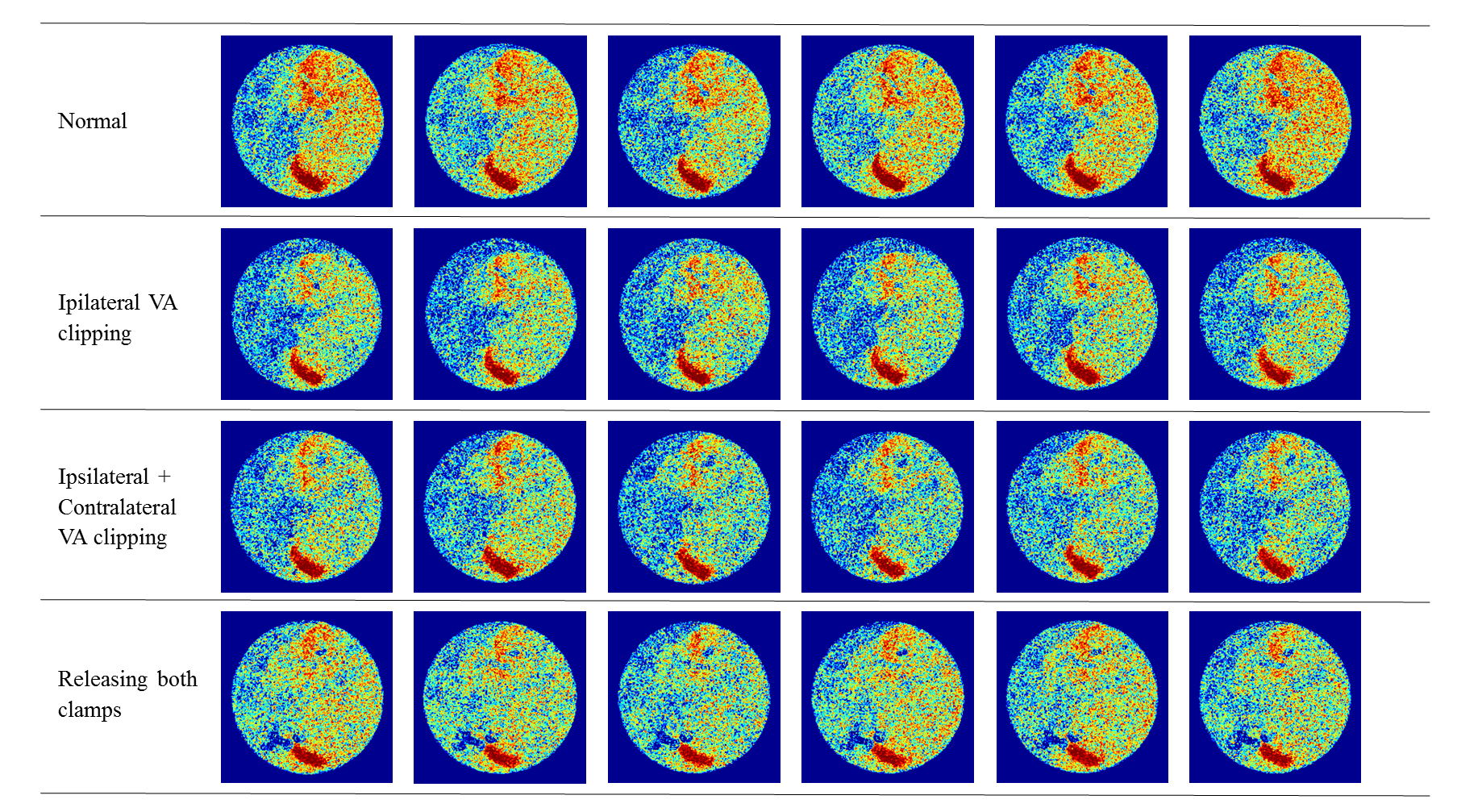

Supplement: S1 Fig — (TIF) [file pone.0191978.s001.tif]
