# Supplementary material for: Monitoring blood-flow in the mouse cochlea using an endoscopic laser speckle contrast imaging system
Source: PLoS One. 2018 Feb 28;13(2):e0191978. doi: 10.1371/journal.pone.0191978 (PMC5830291; doi:10.1371/journal.pone.0191978)
Supplement: S1 File — All items in checklist of ARRIVE guideline were met in our study, confirmed by Young Joon Seo, the corresponding author. (PDF) [file pone.0191978.s002.pdf]

NC3Rs

National Centre for the Replacement Refinement & Reduction of Animals in Research

The ARRIVE Guidelines

Animal Research: Reporting of *In Vivo* Experiments

Carol Kilkenny[1], William J Browne[2], Innes C Cuthill[3], Michael Emerson[4] and Douglas G Altman[5]

[1]The National Centre for the Replacement, Refinement and Reduction of Animals in Research, London, UK , [2]School of Veterinary Science, University of Bristol, Bristol, UK, [3]School of Biological Sciences, University of Bristol, Bristol, UK, [4]National Heart and Lung Institute, Imperial College London, UK, [5]Centre for Statistics in Medicine, University of Oxford, Oxford, UK

The ARRIVE (Animal Research: Reporting of *In Vivo* Experiments) guidelines were developed as part of an NC3Rs initiative to improve the design, analysis and reporting of research using animals – maximising information published and minimising unnecessary studies. The guidelines were published in the online journal *PLOS Biology* in June 2010 and are currently endorsed by scientific journals, major funding bodies and learned societies.

The guidelines are intended to:

- Improve reporting of research using animals.
- Guide authors as to the essential information to include in a manuscript, and not be absolutely prescriptive.
- Be flexible to accommodate reporting a wide range of research areas and experimental protocols.
- Promote reproducible, transparent, accurate, comprehensive, concise, logically ordered, well written manuscripts.
- Improve the communication of the research findings to the broader scientific community.

The guidelines are NOT intended to:

- Promote uniformity, stifle creativity, or encourage authors to adhere rigidly to all items in the checklist. Some of the items may not apply to all studies, and some items can be presented as tables/figure legends or flow diagrams (e.g. the numbers of animals treated, assessed and analysed).
- Be a guide for study design and conduct. However, some items on the checklist, such as randomisation, blinding and using comparator groups, may be useful when planning experiments as their use will reduce the risk of bias and increase the robustness of the research.

Who are the guidelines aimed at?

- Novice and experienced authors
- Journal editors
- Peer reviewers
- Funding bodies

What kind of research areas do the guidelines apply to?

- The guidelines will be most appropriate for comparative studies, where two or more groups of experimental animals are being compared; often one or more of the groups may be considered as a control. They apply also to studies comparing different drug doses, or, for example, where a single animal is used as its own control (within-subject experiment).
- Most of the recommendations also apply to studies that do not have a control group.
- The guidelines are suitable for any area of bioscience research where animals are used.

How might these guidelines be used?

The guidelines provide a checklist for those preparing or reviewing a manuscript intended for publication.

References

1. Kilkenny C, Browne WJ, Cuthill IC, Emerson M, Altman DG (2010) Improving Bioscience Research Reporting: The ARRIVE Guidelines for Reporting Animal Research. *PLOS Biol* 8(6): e1000412. doi:10.1371/journal.pbio.1000412
2. Schulz KF, Altman DG, Moher D, the CONSORT Group (2010) CONSORT 2010 Statement: updated guidelines for reporting parallel group randomised trials. *BMJ* 340:c332.

Funding

The reporting guidelines project was funded by the National Centre for the Replacement, Refinement and Reduction of Animals in Research (NC3Rs).

Acknowledgements

The NC3Rs gratefully acknowledges the expertise and advice that all the contributors have given to developing the guidelines. We would particularly like to acknowledge the contribution of the NC3Rs Reporting Guidelines Working Group. We would also like to thank: NC3Rs grant holders, the Medical Research Council, Biotechnology and Biological Sciences Research Council, Wellcome Trust, Parkinson's Disease Society, British Heart Foundation and their grant holders and funding committee members who provided feedback on the guidelines.

Further Information

www.nc3rs.org.uk/ARRIVE

enquiries@nc3rs.org.uk

@NC3Rs

	ITEM	RECOMMENDATION
Title	1	Provide as accurate and concise a description of the content of the article as possible.
Abstract	2	Provide an accurate summary of the background, research objectives, including details of the species or strain of animal used, key methods, principal findings and conclusions of the study.
**INTRODUCTION**		
Background	3	a. Include sufficient scientific background (including relevant references to previous work) to understand the motivation and context for the study, and explain the experimental approach and rationale.<br><br>b. Explain how and why the animal species and model being used can address the scientific objectives and, where appropriate, the study's relevance to human biology.
Objectives	4	Clearly describe the primary and any secondary objectives of the study, or specific hypotheses being tested.
**METHODS**		
Ethical statement	5	Indicate the nature of the ethical review permissions, relevant licences (e.g. Animal [Scientific Procedures] Act 1986), and national or institutional guidelines for the care and use of animals, that cover the research.
Study design	6	For each experiment, give brief details of the study design including:<br><br>a. The number of experimental and control groups.<br><br>b. Any steps taken to minimise the effects of subjective bias when allocating animals to treatment (e.g. randomisation procedure) and when assessing results (e.g. if done, describe who was blinded and when).<br><br>c. The experimental unit (e.g. a single animal, group or cage of animals).<br><br>A time-line diagram or flow chart can be useful to illustrate how complex study designs were carried out.
Experimental procedures	7	For each experiment and each experimental group, including controls, provide precise details of all procedures carried out.<br><br>For example:<br>a. How (e.g. drug formulation and dose, site and route of administration, anaesthesia and analgesia used [including monitoring], surgical procedure, method of euthanasia). Provide details of any specialist equipment used, including supplier(s).<br><br>b. When (e.g. time of day).<br><br>c. Where (e.g. home cage, laboratory, water maze).<br><br>d. Why (e.g. rationale for choice of specific anaesthetic, route of administration, drug dose used).
Experimental animals	8	a. Provide details of the animals used, including species, strain, sex, developmental stage (e.g. mean or median age plus age range) and weight (e.g. mean or median weight plus weight range).<br><br>b. Provide further relevant information such as the source of animals, international strain nomenclature, genetic modification status (e.g. knock-out or transgenic), genotype, health/immune status, drug or test naïve, previous procedures, etc.
Housing and husbandry	9	Provide details of:<br><br>a. Housing (type of facility e.g. specific pathogen free [SPF]; type of cage or housing; bedding material; number of cage companions; tank shape and material etc. for fish).<br><br>b. Husbandry conditions (e.g. breeding programme, light/dark cycle, temperature, quality of water etc for fish, type of food, access to food and water, environmental enrichment).<br><br>c. Welfare-related assessments and interventions that were carried out prior to, during, or after the experiment.
Sample size	10	a. Specify the total number of animals used in each experiment, and the number of animals in each experimental group.<br><br>b. Explain how the number of animals was arrived at. Provide details of any sample size calculation used.<br><br>c. Indicate the number of independent replications of each experiment, if relevant.
Allocating animals to experimental groups	11	a. Give full details of how animals were allocated to experimental groups, including randomisation or matching if done.<br><br>b. Describe the order in which the animals in the different experimental groups were treated and assessed.
Experimental outcomes	12	Clearly define the primary and secondary experimental outcomes assessed (e.g. cell death, molecular markers, behavioural changes).
Statistical methods	13	a. Provide details of the statistical methods used for each analysis.<br><br>b. Specify the unit of analysis for each dataset (e.g. single animal, group of animals, single neuron).<br><br>c. Describe any methods used to assess whether the data met the assumptions of the statistical approach.
**RESULTS**		
Baseline data	14	For each experimental group, report relevant characteristics and health status of animals (e.g. weight, microbiological status, and drug or test naïve) prior to treatment or testing (this information can often be tabulated).
Numbers analysed	15	a. Report the number of animals in each group included in each analysis. Report absolute numbers (e.g. 10/20, not 50%[2]).<br><br>b. If any animals or data were not included in the analysis, explain why.
Outcomes and estimation	16	Report the results for each analysis carried out, with a measure of precision (e.g. standard error or confidence interval).
Adverse events	17	a. Give details of all important adverse events in each experimental group.<br><br>b. Describe any modifications to the experimental protocols made to reduce adverse events.
**DISCUSSION**		
Interpretation/ scientific implications	18	a. Interpret the results, taking into account the study objectives and hypotheses, current theory and other relevant studies in the literature.<br><br>b. Comment on the study limitations including any potential sources of bias, any limitations of the animal model, and the imprecision associated with the results[2].<br><br>c. Describe any implications of your experimental methods or findings for the replacement, refinement or reduction (the 3Rs) of the use of animals in research.
Generalisability/ translation	19	Comment on whether, and how, the findings of this study are likely to translate to other species or systems, including any relevance to human biology.
Funding	20	List all funding sources (including grant number) and the role of the funder(s) in the study.

confirmed YJ Seo, as Corresponding Author.

ARRIVE

The ARRIVE Guidelines: Animal Research: Reporting of *In Vivo* Experiments. Originally published in *PLOS Biology*, June 2010[1]
