# Supplementary material for: Monitoring blood-flow in the mouse cochlea using an endoscopic laser speckle contrast imaging system
Source: PLoS One. 2018 Feb 28;13(2):e0191978. doi: 10.1371/journal.pone.0191978 (PMC5830291; doi:10.1371/journal.pone.0191978)
Supplement: S2 File — To standardize values, we used the cochlear base–stapes (base/stapes) and cochlear apex–stapes (apex/stapes) ratios of speckle signals. Each mouse follows the number in the table. The number of status is as follows. Status 1: Normal, Status 2: Ipsilateral vertebral artery clamping, Status 3: Bilateral vertebral artery clamping, Status 4: Both release. (PDF) [file pone.0191978.s003.pdf]

| Apex_R | Base_R | Artery_R | MOUSE | Status |
|---|---|---|---|---|
| 150.00 | 177.17 | 275.88 | 1.00 | 1.00 |
| 152.76 | 158.01 | 294.14 | 1.00 | 1.00 |
| 206.46 | 235.16 | 429.20 | 1.00 | 1.00 |
| 149.01 | 153.24 | 274.17 | 1.00 | 1.00 |
| 156.52 | 150.18 | 271.79 | 1.00 | 1.00 |
| 181.09 | 196.29 | 343.54 | 1.00 | 1.00 |
| 168.28 | 184.06 | 319.77 | 1.00 | 1.00 |
| 191.72 | 193.73 | 358.08 | 1.00 | 1.00 |
| 162.52 | 182.37 | 311.17 | 1.00 | 1.00 |
| 178.80 | 175.00 | 318.01 | 1.00 | 1.00 |
| 153.62 | 163.10 | 294.33 | 1.00 | 1.00 |
| 173.37 | 204.34 | 345.80 | 1.00 | 1.00 |
| 192.04 | 202.48 | 348.96 | 1.00 | 1.00 |
| 218.57 | 194.04 | 385.60 | 1.00 | 1.00 |
| 164.37 | 176.63 | 314.81 | 1.00 | 1.00 |
| 179.81 | 187.79 | 323.22 | 1.00 | 1.00 |
| 262.56 | 209.79 | 439.74 | 1.00 | 1.00 |
| 192.25 | 214.36 | 363.39 | 1.00 | 1.00 |
| 170.21 | 188.42 | 327.70 | 1.00 | 1.00 |
| 162.45 | 176.31 | 293.02 | 1.00 | 1.00 |
| 276.56 | 248.65 | 500.52 | 1.00 | 1.00 |
| 176.94 | 186.86 | 327.93 | 1.00 | 1.00 |
| 149.46 | 171.22 | 302.79 | 1.00 | 1.00 |
| 227.03 | 258.63 | 441.55 | 1.00 | 1.00 |
| 244.02 | 230.23 | 444.02 | 1.00 | 1.00 |
| 224.29 | 224.44 | 403.98 | 1.00 | 1.00 |
| 161.92 | 166.47 | 300.06 | 1.00 | 1.00 |
| 223.25 | 225.07 | 394.73 | 1.00 | 1.00 |
| 219.26 | 262.10 | 470.04 | 1.00 | 1.00 |
| 210.63 | 222.44 | 397.77 | 1.00 | 1.00 |
| 179.83 | 190.18 | 332.36 | 1.00 | 1.00 |
| 188.73 | 197.14 | 347.25 | 1.00 | 1.00 |
| 268.71 | 262.34 | 467.02 | 1.00 | 1.00 |
| 262.14 | 303.31 | 520.26 | 1.00 | 1.00 |
| 185.58 | 206.41 | 368.56 | 1.00 | 1.00 |
| 205.82 | 211.52 | 370.64 | 1.00 | 1.00 |
| 256.32 | 266.81 | 453.87 | 1.00 | 1.00 |
| 260.91 | 271.95 | 475.64 | 1.00 | 1.00 |
| 313.79 | 368.50 | 609.70 | 1.00 | 1.00 |
| 171.33 | 199.23 | 354.83 | 1.00 | 1.00 |
| 230.11 | 242.23 | 389.57 | 1.00 | 1.00 |
| 292.51 | 291.88 | 490.39 | 1.00 | 1.00 |

| | | | | |
|---|---|---|---|---|
| 239.49 | 264.06 | 432.18 | 1.00 | 1.00 |
| 305.82 | 307.41 | 553.99 | 1.00 | 1.00 |
| 244.50 | 253.44 | 453.28 | 1.00 | 1.00 |
| 244.42 | 266.15 | 491.13 | 1.00 | 1.00 |
| 213.54 | 249.36 | 364.83 | 1.00 | 1.00 |
| 249.62 | 271.02 | 428.73 | 1.00 | 1.00 |
| 275.66 | 263.62 | 455.10 | 1.00 | 1.00 |
| 268.96 | 277.74 | 476.56 | 1.00 | 1.00 |
| 213.06 | 224.17 | 402.02 | 1.00 | 1.00 |
| 292.24 | 306.24 | 539.85 | 1.00 | 1.00 |
| 170.73 | 213.59 | 336.07 | 1.00 | 1.00 |
| 268.59 | 321.39 | 480.98 | 1.00 | 1.00 |
| 254.74 | 289.94 | 456.80 | 1.00 | 1.00 |
| 302.21 | 318.77 | 549.94 | 1.00 | 1.00 |
| 212.11 | 239.21 | 413.84 | 1.00 | 1.00 |
| 244.46 | 221.81 | 410.64 | 1.00 | 1.00 |
| 244.60 | 288.27 | 467.09 | 1.00 | 1.00 |
| 231.22 | 273.75 | 408.70 | 1.00 | 1.00 |
| 255.27 | 292.14 | 445.00 | 1.00 | 1.00 |
| 230.85 | 282.79 | 443.39 | 1.00 | 1.00 |
| 303.17 | 316.87 | 505.29 | 1.00 | 1.00 |
| 193.44 | 163.46 | 302.03 | 1.00 | 1.00 |
| 262.68 | 294.97 | 512.13 | 1.00 | 1.00 |
| 213.91 | 202.87 | 365.63 | 1.00 | 1.00 |
| 266.79 | 286.20 | 466.42 | 1.00 | 1.00 |
| 193.39 | 225.13 | 364.43 | 1.00 | 1.00 |
| 281.02 | 319.23 | 483.01 | 1.00 | 1.00 |
| 224.12 | 262.10 | 406.13 | 1.00 | 1.00 |
| 158.64 | 141.15 | 266.86 | 1.00 | 1.00 |
| 245.41 | 263.57 | 439.64 | 1.00 | 1.00 |
| 239.85 | 235.86 | 421.89 | 1.00 | 1.00 |
| 241.24 | 257.14 | 429.08 | 1.00 | 1.00 |
| 228.31 | 254.64 | 404.96 | 1.00 | 1.00 |
| 222.40 | 250.22 | 384.84 | 1.00 | 1.00 |
| 160.76 | 148.09 | 273.39 | 1.00 | 1.00 |
| 220.83 | 250.18 | 401.21 | 1.00 | 1.00 |
| 199.63 | 205.06 | 381.72 | 1.00 | 1.00 |
| 308.15 | 336.17 | 561.02 | 1.00 | 1.00 |
| 259.36 | 283.69 | 459.89 | 1.00 | 1.00 |
| 210.30 | 225.36 | 364.06 | 1.00 | 1.00 |
| 160.05 | 176.36 | 297.05 | 1.00 | 1.00 |
| 179.63 | 201.88 | 364.82 | 1.00 | 1.00 |
| 202.17 | 196.12 | 382.16 | 1.00 | 1.00 |

| | | | | |
|---|---|---|---|---|
| 297.51 | 335.12 | 552.89 | 1.00 | 1.00 |
| 322.60 | 353.24 | 575.93 | 1.00 | 1.00 |
| 188.20 | 210.58 | 354.07 | 1.00 | 1.00 |
| 159.09 | 164.94 | 298.69 | 1.00 | 1.00 |
| 232.64 | 245.72 | 426.05 | 1.00 | 1.00 |
| 253.79 | 282.96 | 478.11 | 1.00 | 1.00 |
| 345.25 | 409.78 | 690.69 | 1.00 | 1.00 |
| 165.98 | 218.24 | 350.42 | 1.00 | 1.00 |
| 192.49 | 221.80 | 382.09 | 1.00 | 1.00 |
| 219.78 | 242.22 | 421.11 | 1.00 | 1.00 |
| 317.55 | 365.35 | 620.12 | 1.00 | 1.00 |
| 323.56 | 380.75 | 650.71 | 1.00 | 1.00 |
| 184.05 | 212.74 | 371.69 | 1.00 | 1.00 |
| 232.97 | 268.84 | 472.86 | 1.00 | 1.00 |
| 243.82 | 270.27 | 473.05 | 1.00 | 1.00 |
| 283.80 | 347.47 | 624.78 | 1.00 | 1.00 |
| 220.17 | 275.65 | 469.34 | 1.00 | 1.00 |
| 232.08 | 268.68 | 465.97 | 1.00 | 1.00 |
| 196.18 | 226.38 | 379.75 | 1.00 | 1.00 |
| 212.13 | 267.86 | 456.56 | 1.00 | 1.00 |
| 358.76 | 416.31 | 749.61 | 1.00 | 1.00 |
| 233.21 | 241.19 | 427.84 | 1.00 | 1.00 |
| 151.77 | 170.69 | 318.17 | 1.00 | 1.00 |
| 363.16 | 439.86 | 745.12 | 1.00 | 1.00 |
| 220.64 | 245.23 | 463.34 | 1.00 | 1.00 |
| 167.46 | 173.55 | 332.59 | 1.00 | 1.00 |
| 479.35 | 505.68 | 868.49 | 1.00 | 1.00 |
| 288.51 | 334.15 | 666.80 | 1.00 | 1.00 |
| 327.80 | 312.81 | 686.37 | 1.00 | 1.00 |
| 224.75 | 239.88 | 483.10 | 1.00 | 1.00 |
| 332.12 | 272.63 | 631.99 | 1.00 | 1.00 |
| 202.73 | 243.69 | 460.21 | 1.00 | 1.00 |
| 277.27 | 278.69 | 514.96 | 1.00 | 1.00 |
| 205.74 | 215.27 | 465.95 | 1.00 | 1.00 |
| 323.78 | 365.77 | 657.20 | 1.00 | 1.00 |
| 274.07 | 284.23 | 520.56 | 1.00 | 1.00 |
| 218.22 | 262.92 | 525.20 | 1.00 | 1.00 |
| 319.91 | 332.42 | 659.30 | 1.00 | 1.00 |
| 277.84 | 303.62 | 579.58 | 1.00 | 1.00 |
| 236.85 | 300.56 | 593.91 | 1.00 | 1.00 |
| 302.50 | 344.87 | 631.10 | 1.00 | 1.00 |
| 368.74 | 372.96 | 789.59 | 1.00 | 1.00 |
| 359.18 | 403.53 | 814.93 | 1.00 | 1.00 |

| | | | | |
|---|---|---|---|---|
| 206.34 | 296.70 | 589.25 | 1.00 | 1.00 |
| 312.34 | 357.66 | 692.38 | 1.00 | 1.00 |
| 224.84 | 211.39 | 468.05 | 1.00 | 1.00 |
| 309.43 | 469.08 | 927.81 | 1.00 | 1.00 |
| 205.84 | 256.50 | 517.39 | 1.00 | 1.00 |
| 305.67 | 390.20 | 743.71 | 1.00 | 1.00 |
| 269.45 | 275.18 | 530.90 | 1.00 | 1.00 |
| 174.01 | 215.44 | 448.96 | 1.00 | 1.00 |
| 172.28 | 227.69 | 437.74 | 1.00 | 1.00 |
| 342.84 | 423.58 | 822.49 | 1.00 | 1.00 |
| 324.47 | 416.73 | 745.73 | 1.00 | 1.00 |
| 279.06 | 295.71 | 535.90 | 1.00 | 1.00 |
| 153.78 | 198.61 | 388.18 | 1.00 | 1.00 |
| 164.27 | 195.67 | 404.47 | 1.00 | 1.00 |
| 257.92 | 347.52 | 652.51 | 1.00 | 1.00 |
| 283.59 | 364.98 | 684.38 | 1.00 | 1.00 |
| 262.15 | 299.22 | 527.15 | 1.00 | 1.00 |
| 153.99 | 207.49 | 380.19 | 1.00 | 1.00 |
| 347.71 | 434.31 | 833.12 | 1.00 | 1.00 |
| 263.94 | 269.80 | 535.34 | 1.00 | 1.00 |
| 230.60 | 306.72 | 526.87 | 1.00 | 1.00 |
| 247.22 | 261.07 | 503.40 | 1.00 | 1.00 |
| 211.59 | 264.15 | 489.55 | 1.00 | 1.00 |
| 162.86 | 170.10 | 334.83 | 1.00 | 1.00 |
| 288.95 | 376.52 | 708.94 | 1.00 | 1.00 |
| 254.23 | 263.27 | 528.87 | 1.00 | 1.00 |
| 204.32 | 258.26 | 469.39 | 1.00 | 1.00 |
| 270.06 | 390.47 | 622.73 | 1.00 | 1.00 |
| 238.58 | 286.55 | 531.74 | 1.00 | 1.00 |
| 164.44 | 219.12 | 367.60 | 1.00 | 1.00 |
| 347.86 | 353.41 | 714.87 | 1.00 | 1.00 |
| 263.28 | 306.70 | 601.49 | 1.00 | 1.00 |
| 194.30 | 228.23 | 437.19 | 1.00 | 1.00 |
| 293.33 | 345.11 | 660.78 | 1.00 | 1.00 |
| 403.71 | 582.96 | 972.75 | 1.00 | 1.00 |
| 216.00 | 318.88 | 566.64 | 1.00 | 1.00 |
| 197.48 | 242.80 | 405.97 | 1.00 | 1.00 |
| 210.90 | 227.79 | 464.16 | 1.00 | 1.00 |
| 250.63 | 299.80 | 566.66 | 1.00 | 1.00 |
| 237.50 | 312.02 | 575.71 | 1.00 | 1.00 |
| 432.63 | 534.50 | 1021.38 | 1.00 | 1.00 |
| 287.20 | 453.90 | 726.88 | 1.00 | 1.00 |
| 249.28 | 312.62 | 516.93 | 1.00 | 1.00 |

| | | | | |
|---|---|---|---|---|
| 243.52 | 309.62 | 551.52 | 1.00 | 1.00 |
| 207.67 | 243.32 | 395.31 | 1.00 | 2.00 |
| 182.93 | 209.40 | 389.42 | 1.00 | 2.00 |
| 254.49 | 280.37 | 519.58 | 1.00 | 2.00 |
| 291.78 | 300.09 | 540.62 | 1.00 | 2.00 |
| 198.50 | 174.55 | 362.43 | 1.00 | 2.00 |
| 260.46 | 258.35 | 484.51 | 1.00 | 2.00 |
| 197.32 | 186.80 | 345.53 | 1.00 | 2.00 |
| 189.89 | 182.72 | 384.90 | 1.00 | 2.00 |
| 170.76 | 194.38 | 334.59 | 1.00 | 2.00 |
| 189.60 | 198.49 | 365.98 | 1.00 | 2.00 |
| 212.92 | 219.62 | 452.15 | 1.00 | 2.00 |
| 197.22 | 200.32 | 373.28 | 1.00 | 2.00 |
| 281.50 | 278.61 | 546.62 | 1.00 | 2.00 |
| 198.15 | 230.77 | 445.97 | 1.00 | 2.00 |
| 147.55 | 178.95 | 338.61 | 1.00 | 2.00 |
| 216.99 | 198.73 | 397.39 | 1.00 | 2.00 |
| 224.62 | 185.58 | 429.36 | 1.00 | 2.00 |
| 182.57 | 204.06 | 391.24 | 1.00 | 2.00 |
| 179.47 | 193.54 | 369.59 | 1.00 | 2.00 |
| 218.70 | 180.75 | 395.19 | 1.00 | 2.00 |
| 293.46 | 248.60 | 572.40 | 1.00 | 2.00 |
| 212.83 | 218.61 | 432.23 | 1.00 | 2.00 |
| 177.75 | 189.05 | 368.18 | 1.00 | 2.00 |
| 188.49 | 187.98 | 385.76 | 1.00 | 2.00 |
| 292.41 | 252.74 | 558.74 | 1.00 | 2.00 |
| 250.93 | 246.60 | 460.71 | 1.00 | 2.00 |
| 196.74 | 222.00 | 410.33 | 1.00 | 2.00 |
| 233.64 | 229.81 | 474.70 | 1.00 | 2.00 |
| 210.44 | 222.72 | 473.31 | 1.00 | 2.00 |
| 245.09 | 223.80 | 485.67 | 1.00 | 2.00 |
| 278.52 | 268.11 | 488.92 | 1.00 | 2.00 |
| 287.98 | 281.17 | 592.44 | 1.00 | 2.00 |
| 255.09 | 252.07 | 485.70 | 1.00 | 2.00 |
| 226.34 | 213.90 | 466.50 | 1.00 | 2.00 |
| 179.69 | 192.51 | 404.24 | 1.00 | 2.00 |
| 230.43 | 258.56 | 476.54 | 1.00 | 2.00 |
| 326.88 | 283.48 | 561.71 | 1.00 | 2.00 |
| 186.10 | 211.63 | 376.81 | 1.00 | 2.00 |
| 240.54 | 229.15 | 490.61 | 1.00 | 2.00 |
| 181.10 | 175.61 | 364.59 | 1.00 | 2.00 |
| 191.03 | 214.64 | 431.27 | 1.00 | 2.00 |
| 275.67 | 266.08 | 573.17 | 1.00 | 2.00 |

| | | | | |
|---|---|---|---|---|
| 219.17 | 206.68 | 426.83 | 1.00 | 2.00 |
| 239.40 | 208.29 | 440.62 | 1.00 | 2.00 |
| 150.44 | 139.59 | 305.37 | 1.00 | 2.00 |
| 152.91 | 142.88 | 309.70 | 1.00 | 2.00 |
| 207.13 | 192.70 | 407.89 | 1.00 | 2.00 |
| 230.91 | 238.70 | 505.81 | 1.00 | 2.00 |
| 184.42 | 172.12 | 374.87 | 1.00 | 2.00 |
| 313.59 | 271.68 | 622.71 | 1.00 | 2.00 |
| 185.31 | 147.44 | 328.31 | 1.00 | 2.00 |
| 150.93 | 135.48 | 300.42 | 1.00 | 2.00 |
| 196.97 | 162.94 | 357.18 | 1.00 | 2.00 |
| 271.46 | 267.60 | 532.90 | 1.00 | 2.00 |
| 208.20 | 205.78 | 427.37 | 1.00 | 2.00 |
| 368.96 | 342.39 | 754.22 | 1.00 | 2.00 |
| 249.76 | 226.86 | 494.96 | 1.00 | 2.00 |
| 208.28 | 175.20 | 382.13 | 1.00 | 2.00 |
| 172.20 | 172.67 | 349.23 | 1.00 | 2.00 |
| 260.59 | 208.09 | 457.55 | 1.00 | 2.00 |
| 221.80 | 249.59 | 486.58 | 1.00 | 2.00 |
| 290.25 | 293.57 | 585.90 | 1.00 | 2.00 |
| 658.80 | 693.66 | 1422.83 | 1.00 | 2.00 |
| 226.88 | 201.19 | 417.44 | 1.00 | 2.00 |
| 149.85 | 147.77 | 315.70 | 1.00 | 2.00 |
| 223.61 | 166.60 | 372.55 | 1.00 | 2.00 |
| 189.55 | 203.83 | 421.45 | 1.00 | 2.00 |
| 255.41 | 256.77 | 489.91 | 1.00 | 2.00 |
| 319.05 | 337.08 | 669.28 | 1.00 | 2.00 |
| 467.59 | 453.11 | 884.94 | 1.00 | 2.00 |
| 186.29 | 191.47 | 412.36 | 1.00 | 2.00 |
| 268.50 | 212.16 | 433.83 | 1.00 | 2.00 |
| 169.45 | 192.39 | 392.90 | 1.00 | 2.00 |
| 228.30 | 252.48 | 481.89 | 1.00 | 2.00 |
| 163.12 | 172.47 | 339.63 | 1.00 | 2.00 |
| 220.51 | 263.63 | 512.63 | 1.00 | 2.00 |
| 361.02 | 387.70 | 768.10 | 1.00 | 2.00 |
| 197.27 | 186.77 | 404.17 | 1.00 | 2.00 |
| 216.11 | 223.75 | 419.93 | 1.00 | 2.00 |
| 196.57 | 195.41 | 375.74 | 1.00 | 2.00 |
| 270.37 | 270.29 | 527.69 | 1.00 | 2.00 |
| 144.34 | 165.75 | 334.13 | 1.00 | 2.00 |
| 254.27 | 255.44 | 468.38 | 1.00 | 2.00 |
| 269.98 | 245.26 | 514.07 | 1.00 | 2.00 |
| 253.25 | 232.47 | 460.94 | 1.00 | 2.00 |

| | | | | |
|---|---|---|---|---|
| 219.91 | 251.27 | 475.58 | 1.00 | 2.00 |
| 143.42 | 184.01 | 333.12 | 1.00 | 2.00 |
| 200.18 | 208.48 | 411.19 | 1.00 | 2.00 |
| 176.73 | 195.15 | 403.75 | 1.00 | 2.00 |
| 159.33 | 159.37 | 369.43 | 1.00 | 2.00 |
| 189.06 | 224.97 | 492.80 | 1.00 | 2.00 |
| 130.94 | 161.57 | 305.35 | 1.00 | 2.00 |
| 161.19 | 236.81 | 459.84 | 1.00 | 2.00 |
| 198.66 | 269.33 | 544.85 | 1.00 | 2.00 |
| 157.15 | 183.95 | 350.82 | 1.00 | 2.00 |
| 162.75 | 228.02 | 444.58 | 1.00 | 2.00 |
| 141.47 | 164.91 | 313.46 | 1.00 | 2.00 |
| 158.17 | 205.86 | 395.86 | 1.00 | 2.00 |
| 162.40 | 248.03 | 454.44 | 1.00 | 2.00 |
| 141.27 | 190.37 | 334.85 | 1.00 | 2.00 |
| 173.13 | 195.63 | 377.17 | 1.00 | 2.00 |
| 137.35 | 198.20 | 385.73 | 1.00 | 2.00 |
| 177.95 | 239.08 | 456.16 | 1.00 | 2.00 |
| 147.37 | 178.28 | 333.29 | 1.00 | 2.00 |
| 119.88 | 149.03 | 274.58 | 1.00 | 2.00 |
| 132.05 | 160.06 | 311.07 | 1.00 | 2.00 |
| 182.32 | 265.50 | 517.45 | 1.00 | 2.00 |
| 141.06 | 179.34 | 328.43 | 1.00 | 2.00 |
| 115.92 | 150.04 | 287.07 | 1.00 | 2.00 |
| 138.61 | 173.64 | 331.46 | 1.00 | 2.00 |
| 218.15 | 290.33 | 592.06 | 1.00 | 2.00 |
| 123.64 | 178.61 | 326.40 | 1.00 | 2.00 |
| 145.32 | 200.86 | 385.53 | 1.00 | 2.00 |
| 162.50 | 207.47 | 408.34 | 1.00 | 2.00 |
| 221.19 | 257.87 | 480.31 | 1.00 | 2.00 |
| 141.79 | 207.87 | 383.60 | 1.00 | 2.00 |
| 117.18 | 159.09 | 288.59 | 1.00 | 2.00 |
| 243.43 | 271.25 | 591.42 | 1.00 | 2.00 |
| 156.81 | 233.85 | 389.56 | 1.00 | 2.00 |
| 172.87 | 248.84 | 428.08 | 1.00 | 2.00 |
| 203.18 | 235.98 | 446.64 | 1.00 | 2.00 |
| 142.12 | 143.83 | 288.99 | 1.00 | 2.00 |
| 190.23 | 200.96 | 435.55 | 1.00 | 2.00 |
| 176.36 | 181.40 | 378.18 | 1.00 | 2.00 |
| 180.06 | 242.55 | 428.52 | 1.00 | 2.00 |
| 162.46 | 250.75 | 485.07 | 1.00 | 2.00 |
| 171.56 | 217.86 | 401.24 | 1.00 | 2.00 |
| 181.75 | 193.70 | 388.88 | 1.00 | 2.00 |

| | | | | |
|---|---|---|---|---|
| 267.20 | 289.85 | 588.63 | 1.00 | 2.00 |
| 187.00 | 202.58 | 371.41 | 1.00 | 2.00 |
| 175.94 | 216.01 | 437.30 | 1.00 | 2.00 |
| 294.65 | 330.42 | 687.30 | 1.00 | 2.00 |
| 169.04 | 253.00 | 469.97 | 1.00 | 2.00 |
| 170.66 | 187.89 | 387.25 | 1.00 | 2.00 |
| 206.92 | 211.01 | 435.84 | 1.00 | 2.00 |
| 200.63 | 222.06 | 414.59 | 1.00 | 2.00 |
| 223.56 | 257.10 | 526.45 | 1.00 | 2.00 |
| 335.12 | 480.11 | 836.47 | 1.00 | 2.00 |
| 205.49 | 227.79 | 447.45 | 1.00 | 2.00 |
| 152.56 | 206.05 | 419.91 | 1.00 | 2.00 |
| 180.69 | 249.88 | 453.59 | 1.00 | 2.00 |
| 156.46 | 171.26 | 339.11 | 1.00 | 2.00 |
| 184.06 | 207.47 | 419.10 | 1.00 | 2.00 |
| 294.40 | 357.01 | 663.77 | 1.00 | 2.00 |
| 236.00 | 274.36 | 608.22 | 1.00 | 2.00 |
| 230.13 | 279.23 | 524.61 | 1.00 | 2.00 |
| 214.36 | 228.18 | 452.03 | 1.00 | 2.00 |
| 156.89 | 227.82 | 429.96 | 1.00 | 2.00 |
| 154.24 | 192.83 | 334.77 | 1.00 | 2.00 |
| 303.43 | 310.48 | 697.07 | 1.00 | 2.00 |
| 239.13 | 258.60 | 499.50 | 1.00 | 2.00 |
| 196.39 | 263.79 | 493.92 | 1.00 | 2.00 |
| 243.25 | 344.95 | 604.62 | 1.00 | 2.00 |
| 235.44 | 232.85 | 472.45 | 1.00 | 2.00 |
| 125.98 | 183.76 | 343.20 | 1.00 | 2.00 |
| 207.36 | 223.36 | 433.45 | 1.00 | 2.00 |
| 201.82 | 263.49 | 489.65 | 1.00 | 2.00 |
| 287.11 | 278.32 | 604.99 | 1.00 | 2.00 |
| 223.81 | 307.08 | 543.63 | 1.00 | 2.00 |
| 178.41 | 228.35 | 430.14 | 1.00 | 2.00 |
| 188.93 | 216.36 | 411.98 | 1.00 | 2.00 |
| 173.20 | 233.66 | 405.04 | 1.00 | 2.00 |
| 202.83 | 227.00 | 445.84 | 1.00 | 2.00 |
| 233.98 | 276.55 | 565.03 | 1.00 | 2.00 |
| 180.11 | 231.17 | 413.91 | 1.00 | 2.00 |
| 133.59 | 168.10 | 345.89 | 1.00 | 2.00 |
| 216.26 | 231.00 | 449.32 | 1.00 | 2.00 |
| 158.73 | 221.51 | 420.92 | 1.00 | 2.00 |
| 163.27 | 197.04 | 346.23 | 1.00 | 2.00 |
| 137.34 | 188.66 | 357.34 | 1.00 | 2.00 |
| 192.07 | 177.49 | 354.89 | 1.00 | 3.00 |

| | | | | |
|---|---|---|---|---|
| 210.76 | 220.65 | 393.47 | 1.00 | 3.00 |
| 227.55 | 202.51 | 438.82 | 1.00 | 3.00 |
| 186.26 | 181.42 | 340.87 | 1.00 | 3.00 |
| 171.87 | 218.45 | 436.87 | 1.00 | 3.00 |
| 189.22 | 180.44 | 329.78 | 1.00 | 3.00 |
| 132.70 | 137.61 | 305.80 | 1.00 | 3.00 |
| 154.71 | 181.26 | 371.41 | 1.00 | 3.00 |
| 185.69 | 168.91 | 330.76 | 1.00 | 3.00 |
| 140.46 | 164.65 | 363.88 | 1.00 | 3.00 |
| 148.46 | 158.89 | 331.51 | 1.00 | 3.00 |
| 170.81 | 162.83 | 325.59 | 1.00 | 3.00 |
| 146.96 | 170.66 | 365.96 | 1.00 | 3.00 |
| 163.84 | 182.54 | 362.58 | 1.00 | 3.00 |
| 161.55 | 180.27 | 373.72 | 1.00 | 3.00 |
| 175.02 | 166.26 | 340.85 | 1.00 | 3.00 |
| 184.36 | 213.08 | 424.14 | 1.00 | 3.00 |
| 194.92 | 189.16 | 384.04 | 1.00 | 3.00 |
| 132.24 | 153.05 | 305.88 | 1.00 | 3.00 |
| 131.52 | 151.99 | 284.85 | 1.00 | 3.00 |
| 157.28 | 183.50 | 334.80 | 1.00 | 3.00 |
| 158.06 | 161.82 | 331.22 | 1.00 | 3.00 |
| 150.07 | 158.66 | 313.70 | 1.00 | 3.00 |
| 149.73 | 177.39 | 351.54 | 1.00 | 3.00 |
| 134.66 | 168.70 | 317.27 | 1.00 | 3.00 |
| 156.40 | 156.25 | 309.55 | 1.00 | 3.00 |
| 192.70 | 186.17 | 389.04 | 1.00 | 3.00 |
| 197.04 | 188.79 | 366.07 | 1.00 | 3.00 |
| 223.84 | 213.08 | 435.57 | 1.00 | 3.00 |
| 130.27 | 161.43 | 309.18 | 1.00 | 3.00 |
| 147.68 | 151.18 | 307.92 | 1.00 | 3.00 |
| 201.52 | 196.59 | 414.16 | 1.00 | 3.00 |
| 245.79 | 231.00 | 458.50 | 1.00 | 3.00 |
| 159.15 | 161.23 | 321.11 | 1.00 | 3.00 |
| 240.95 | 222.84 | 458.26 | 1.00 | 3.00 |
| 145.33 | 157.28 | 312.23 | 1.00 | 3.00 |
| 158.10 | 148.27 | 308.02 | 1.00 | 3.00 |
| 187.86 | 197.71 | 390.86 | 1.00 | 3.00 |
| 224.03 | 217.72 | 429.92 | 1.00 | 3.00 |
| 220.01 | 245.98 | 480.34 | 1.00 | 3.00 |
| 215.60 | 219.87 | 440.61 | 1.00 | 3.00 |
| 169.43 | 185.22 | 373.53 | 1.00 | 3.00 |
| 185.37 | 194.00 | 380.78 | 1.00 | 3.00 |
| 257.99 | 236.19 | 491.60 | 1.00 | 3.00 |

| | | | | |
|---|---|---|---|---|
| 154.54 | 157.17 | 323.30 | 1.00 | 3.00 |
| 269.19 | 267.90 | 505.67 | 1.00 | 3.00 |
| 163.45 | 178.08 | 389.56 | 1.00 | 3.00 |
| 243.75 | 282.92 | 500.22 | 1.00 | 3.00 |
| 245.31 | 241.59 | 452.75 | 1.00 | 3.00 |
| 192.61 | 205.72 | 401.35 | 1.00 | 3.00 |
| 253.20 | 200.61 | 484.36 | 1.00 | 3.00 |
| 160.08 | 161.72 | 320.85 | 1.00 | 3.00 |
| 255.23 | 241.39 | 486.09 | 1.00 | 3.00 |
| 197.47 | 231.60 | 454.88 | 1.00 | 3.00 |
| 159.20 | 241.70 | 411.31 | 1.00 | 3.00 |
| 210.45 | 248.85 | 464.23 | 1.00 | 3.00 |
| 232.11 | 220.89 | 456.15 | 1.00 | 3.00 |
| 228.87 | 198.67 | 429.18 | 1.00 | 3.00 |
| 204.82 | 204.48 | 381.97 | 1.00 | 3.00 |
| 201.38 | 190.41 | 402.86 | 1.00 | 3.00 |
| 302.25 | 261.32 | 605.75 | 1.00 | 3.00 |
| 147.05 | 198.50 | 356.31 | 1.00 | 3.00 |
| 139.69 | 182.83 | 349.87 | 1.00 | 3.00 |
| 170.87 | 194.07 | 392.20 | 1.00 | 3.00 |
| 217.86 | 199.65 | 417.76 | 1.00 | 3.00 |
| 227.54 | 239.60 | 427.19 | 1.00 | 3.00 |
| 179.99 | 181.40 | 349.66 | 1.00 | 3.00 |
| 274.28 | 253.02 | 557.55 | 1.00 | 3.00 |
| 188.29 | 195.82 | 374.37 | 1.00 | 3.00 |
| 120.44 | 159.50 | 313.72 | 1.00 | 3.00 |
| 184.71 | 206.82 | 405.12 | 1.00 | 3.00 |
| 254.61 | 240.32 | 503.49 | 1.00 | 3.00 |
| 213.19 | 229.39 | 430.74 | 1.00 | 3.00 |
| 160.84 | 151.88 | 331.07 | 1.00 | 3.00 |
| 248.42 | 225.07 | 469.32 | 1.00 | 3.00 |
| 179.14 | 183.22 | 379.18 | 1.00 | 3.00 |
| 146.15 | 198.62 | 361.56 | 1.00 | 3.00 |
| 185.92 | 161.87 | 376.32 | 1.00 | 3.00 |
| 197.30 | 239.71 | 436.63 | 1.00 | 3.00 |
| 199.35 | 195.20 | 421.42 | 1.00 | 3.00 |
| 142.88 | 155.56 | 322.00 | 1.00 | 3.00 |
| 201.57 | 174.03 | 356.64 | 1.00 | 3.00 |
| 171.41 | 185.18 | 344.42 | 1.00 | 3.00 |
| 177.72 | 182.29 | 395.17 | 1.00 | 3.00 |
| 195.56 | 234.75 | 414.79 | 1.00 | 3.00 |
| 247.44 | 249.22 | 507.29 | 1.00 | 3.00 |
| 140.47 | 167.10 | 306.84 | 1.00 | 3.00 |

| | | | | |
|---|---|---|---|---|
| 182.12 | 171.37 | 362.82 | 1.00 | 3.00 |
| 191.00 | 181.23 | 340.94 | 1.00 | 3.00 |
| 194.22 | 227.13 | 425.34 | 1.00 | 3.00 |
| 192.76 | 226.49 | 406.55 | 1.00 | 3.00 |
| 236.88 | 248.53 | 498.72 | 1.00 | 3.00 |
| 151.56 | 168.44 | 336.96 | 1.00 | 3.00 |
| 175.79 | 168.26 | 347.95 | 1.00 | 3.00 |
| 175.25 | 162.50 | 326.59 | 1.00 | 3.00 |
| 175.74 | 192.99 | 372.84 | 1.00 | 3.00 |
| 203.75 | 193.48 | 406.55 | 1.00 | 3.00 |
| 193.80 | 232.17 | 436.09 | 1.00 | 3.00 |
| 147.46 | 174.79 | 340.98 | 1.00 | 3.00 |
| 182.02 | 188.13 | 359.47 | 1.00 | 3.00 |
| 164.39 | 143.87 | 317.07 | 1.00 | 3.00 |
| 169.22 | 183.26 | 345.24 | 1.00 | 3.00 |
| 203.33 | 192.68 | 363.97 | 1.00 | 3.00 |
| 155.29 | 188.27 | 359.54 | 1.00 | 3.00 |
| 170.17 | 165.50 | 352.72 | 1.00 | 3.00 |
| 184.71 | 182.14 | 354.23 | 1.00 | 3.00 |
| 186.02 | 179.05 | 363.19 | 1.00 | 3.00 |
| 190.88 | 183.69 | 385.12 | 1.00 | 3.00 |
| 154.31 | 172.35 | 356.23 | 1.00 | 3.00 |
| 142.54 | 220.77 | 364.81 | 1.00 | 3.00 |
| 132.82 | 205.87 | 342.19 | 1.00 | 3.00 |
| 167.90 | 218.77 | 393.15 | 1.00 | 3.00 |
| 146.37 | 205.81 | 343.08 | 1.00 | 3.00 |
| 180.06 | 226.32 | 408.29 | 1.00 | 3.00 |
| 155.77 | 204.92 | 373.91 | 1.00 | 3.00 |
| 165.38 | 227.04 | 433.97 | 1.00 | 3.00 |
| 154.90 | 216.22 | 398.53 | 1.00 | 3.00 |
| 162.37 | 252.17 | 431.70 | 1.00 | 3.00 |
| 202.85 | 248.32 | 483.11 | 1.00 | 3.00 |
| 163.22 | 233.74 | 411.15 | 1.00 | 3.00 |
| 165.71 | 228.05 | 398.80 | 1.00 | 3.00 |
| 231.90 | 298.08 | 505.92 | 1.00 | 3.00 |
| 157.43 | 217.00 | 407.68 | 1.00 | 3.00 |
| 201.09 | 256.64 | 451.25 | 1.00 | 3.00 |
| 126.68 | 188.54 | 330.47 | 1.00 | 3.00 |
| 185.40 | 214.72 | 396.94 | 1.00 | 3.00 |
| 189.16 | 271.17 | 461.80 | 1.00 | 3.00 |
| 219.43 | 251.35 | 471.33 | 1.00 | 3.00 |
| 141.50 | 199.00 | 340.85 | 1.00 | 3.00 |
| 209.59 | 265.02 | 441.67 | 1.00 | 3.00 |

| | | | | |
|---|---|---|---|---|
| 175.92 | 226.79 | 443.44 | 1.00 | 3.00 |
| 207.89 | 265.66 | 453.29 | 1.00 | 3.00 |
| 150.65 | 188.38 | 355.29 | 1.00 | 3.00 |
| 184.42 | 225.38 | 400.70 | 1.00 | 3.00 |
| 221.16 | 311.76 | 499.40 | 1.00 | 3.00 |
| 197.25 | 236.49 | 472.32 | 1.00 | 3.00 |
| 209.48 | 272.47 | 441.02 | 1.00 | 3.00 |
| 136.45 | 178.43 | 329.25 | 1.00 | 3.00 |
| 181.51 | 198.12 | 357.38 | 1.00 | 3.00 |
| 210.12 | 249.31 | 445.04 | 1.00 | 3.00 |
| 227.79 | 283.87 | 485.25 | 1.00 | 3.00 |
| 185.29 | 211.47 | 378.49 | 1.00 | 3.00 |
| 134.63 | 183.70 | 300.76 | 1.00 | 3.00 |
| 138.48 | 171.70 | 302.78 | 1.00 | 3.00 |
| 195.63 | 252.69 | 422.86 | 1.00 | 3.00 |
| 145.38 | 202.86 | 354.84 | 1.00 | 3.00 |
| 241.45 | 314.35 | 535.24 | 1.00 | 3.00 |
| 169.22 | 177.14 | 336.77 | 1.00 | 3.00 |
| 118.90 | 169.07 | 284.12 | 1.00 | 3.00 |
| 139.27 | 189.05 | 349.53 | 1.00 | 3.00 |
| 146.29 | 195.84 | 352.61 | 1.00 | 3.00 |
| 173.46 | 253.43 | 446.88 | 1.00 | 3.00 |
| 172.10 | 240.81 | 408.62 | 1.00 | 3.00 |
| 170.15 | 198.74 | 332.12 | 1.00 | 3.00 |
| 124.86 | 169.19 | 280.00 | 1.00 | 3.00 |
| 137.42 | 204.84 | 353.63 | 1.00 | 3.00 |
| 162.41 | 213.79 | 388.09 | 1.00 | 3.00 |
| 137.92 | 185.34 | 345.76 | 1.00 | 3.00 |
| 126.83 | 168.84 | 290.80 | 1.00 | 3.00 |
| 158.86 | 200.22 | 339.28 | 1.00 | 3.00 |
| 161.95 | 237.24 | 397.15 | 1.00 | 3.00 |
| 108.17 | 175.88 | 303.96 | 1.00 | 3.00 |
| 151.74 | 195.79 | 359.37 | 1.00 | 3.00 |
| 134.11 | 177.98 | 319.37 | 1.00 | 3.00 |
| 145.63 | 180.03 | 320.71 | 1.00 | 3.00 |
| 143.43 | 178.29 | 315.26 | 1.00 | 3.00 |
| 129.28 | 233.65 | 364.45 | 1.00 | 3.00 |
| 127.03 | 183.93 | 332.55 | 1.00 | 3.00 |
| 151.51 | 193.01 | 344.37 | 1.00 | 3.00 |
| 176.08 | 231.67 | 405.71 | 1.00 | 3.00 |
| 158.53 | 200.89 | 341.87 | 1.00 | 3.00 |
| 124.03 | 163.37 | 299.24 | 1.00 | 3.00 |
| 141.40 | 190.51 | 355.27 | 1.00 | 3.00 |

| | | | | |
|---|---|---|---|---|
| 150.56 | 188.75 | 351.36 | 1.00 | 3.00 |
| 127.79 | 184.84 | 319.18 | 1.00 | 3.00 |
| 161.81 | 210.01 | 357.75 | 1.00 | 3.00 |
| 128.95 | 169.18 | 303.13 | 1.00 | 3.00 |
| 151.69 | 184.06 | 342.71 | 1.00 | 3.00 |
| 171.61 | 197.55 | 365.62 | 1.00 | 3.00 |
| 165.97 | 213.10 | 372.97 | 1.00 | 3.00 |
| 178.52 | 214.64 | 377.71 | 1.00 | 3.00 |
| 216.83 | 259.39 | 467.20 | 1.00 | 3.00 |
| 184.74 | 220.03 | 381.88 | 1.00 | 3.00 |
| 157.08 | 200.29 | 332.57 | 1.00 | 3.00 |
| 229.00 | 310.26 | 536.78 | 1.00 | 3.00 |
| 179.96 | 198.58 | 372.50 | 1.00 | 3.00 |
| 154.96 | 195.97 | 343.37 | 1.00 | 3.00 |
| 232.57 | 297.29 | 502.78 | 1.00 | 3.00 |
| 174.34 | 230.88 | 394.84 | 1.00 | 3.00 |
| 195.02 | 283.88 | 493.10 | 1.00 | 3.00 |
| 210.78 | 242.92 | 431.78 | 1.00 | 3.00 |
| 156.48 | 221.30 | 400.25 | 1.00 | 3.00 |
| 188.71 | 249.16 | 467.53 | 1.00 | 3.00 |
| 165.37 | 237.90 | 426.31 | 1.00 | 3.00 |
| 213.78 | 213.20 | 237.73 | 1.00 | 4.00 |
| 217.49 | 211.07 | 255.36 | 1.00 | 4.00 |
| 217.42 | 213.13 | 212.06 | 1.00 | 4.00 |
| 216.41 | 198.62 | 263.65 | 1.00 | 4.00 |
| 204.83 | 204.68 | 281.55 | 1.00 | 4.00 |
| 232.14 | 186.75 | 243.59 | 1.00 | 4.00 |
| 196.49 | 214.19 | 270.92 | 1.00 | 4.00 |
| 251.77 | 202.69 | 265.52 | 1.00 | 4.00 |
| 237.30 | 208.85 | 236.50 | 1.00 | 4.00 |
| 208.71 | 211.27 | 266.18 | 1.00 | 4.00 |
| 244.14 | 197.13 | 247.73 | 1.00 | 4.00 |
| 211.08 | 216.70 | 251.98 | 1.00 | 4.00 |
| 195.27 | 206.59 | 272.91 | 1.00 | 4.00 |
| 224.50 | 201.44 | 245.23 | 1.00 | 4.00 |
| 215.46 | 239.98 | 244.20 | 1.00 | 4.00 |
| 203.78 | 220.88 | 254.86 | 1.00 | 4.00 |
| 202.39 | 202.53 | 242.92 | 1.00 | 4.00 |
| 221.19 | 209.14 | 229.96 | 1.00 | 4.00 |
| 209.88 | 246.19 | 224.94 | 1.00 | 4.00 |
| 220.27 | 228.63 | 260.57 | 1.00 | 4.00 |
| 199.05 | 213.70 | 226.26 | 1.00 | 4.00 |
| 225.62 | 214.07 | 246.11 | 1.00 | 4.00 |

| | | | | |
|---|---|---|---|---|
| 213.55 | 247.08 | 217.31 | 1.00 | 4.00 |
| 243.85 | 235.37 | 238.20 | 1.00 | 4.00 |
| 212.41 | 226.08 | 225.99 | 1.00 | 4.00 |
| 208.91 | 197.70 | 239.48 | 1.00 | 4.00 |
| 231.48 | 264.83 | 268.89 | 1.00 | 4.00 |
| 217.03 | 239.61 | 227.01 | 1.00 | 4.00 |
| 226.58 | 230.75 | 246.35 | 1.00 | 4.00 |
| 200.55 | 203.37 | 216.75 | 1.00 | 4.00 |
| 214.91 | 209.68 | 257.34 | 1.00 | 4.00 |
| 223.96 | 258.17 | 237.15 | 1.00 | 4.00 |
| 209.22 | 244.12 | 231.33 | 1.00 | 4.00 |
| 225.06 | 222.83 | 234.64 | 1.00 | 4.00 |
| 212.32 | 202.23 | 213.53 | 1.00 | 4.00 |
| 201.90 | 200.82 | 266.53 | 1.00 | 4.00 |
| 215.74 | 231.81 | 225.63 | 1.00 | 4.00 |
| 207.49 | 251.51 | 226.76 | 1.00 | 4.00 |
| 228.47 | 214.65 | 254.94 | 1.00 | 4.00 |
| 214.36 | 210.78 | 226.87 | 1.00 | 4.00 |
| 189.28 | 205.10 | 268.74 | 1.00 | 4.00 |
| 204.37 | 216.06 | 230.59 | 1.00 | 4.00 |
| 202.75 | 228.32 | 238.81 | 1.00 | 4.00 |
| 199.70 | 238.33 | 273.24 | 1.00 | 4.00 |
| 234.72 | 215.54 | 259.65 | 1.00 | 4.00 |
| 223.60 | 210.21 | 242.42 | 1.00 | 4.00 |
| 201.77 | 211.55 | 232.26 | 1.00 | 4.00 |
| 217.97 | 225.52 | 260.42 | 1.00 | 4.00 |
| 198.27 | 228.06 | 263.70 | 1.00 | 4.00 |
| 203.54 | 244.27 | 256.92 | 1.00 | 4.00 |
| 228.76 | 227.91 | 226.34 | 1.00 | 4.00 |
| 237.01 | 219.10 | 257.02 | 1.00 | 4.00 |
| 231.50 | 227.00 | 260.56 | 1.00 | 4.00 |
| 217.53 | 234.98 | 258.63 | 1.00 | 4.00 |
| 215.71 | 228.42 | 220.55 | 1.00 | 4.00 |
| 195.33 | 226.88 | 287.91 | 1.00 | 4.00 |
| 209.29 | 248.35 | 248.92 | 1.00 | 4.00 |
| 250.24 | 229.34 | 254.74 | 1.00 | 4.00 |
| 235.96 | 228.11 | 242.15 | 1.00 | 4.00 |
| 204.42 | 239.57 | 263.48 | 1.00 | 4.00 |
| 221.83 | 245.71 | 255.10 | 1.00 | 4.00 |
| 227.71 | 230.62 | 299.97 | 1.00 | 4.00 |
| 206.83 | 242.49 | 250.82 | 1.00 | 4.00 |
| 245.73 | 254.33 | 252.34 | 1.00 | 4.00 |
| 252.34 | 226.67 | 303.45 | 1.00 | 4.00 |

| | | | | |
|---|---|---|---|---|
| 263.58 | 242.84 | 248.95 | 1.00 | 4.00 |
| 196.20 | 232.63 | 267.75 | 1.00 | 4.00 |
| 247.15 | 247.43 | 300.40 | 1.00 | 4.00 |
| 215.31 | 236.67 | 258.75 | 1.00 | 4.00 |
| 201.51 | 249.67 | 291.07 | 1.00 | 4.00 |
| 251.91 | 249.29 | 264.63 | 1.00 | 4.00 |
| 240.24 | 232.54 | 229.72 | 1.00 | 4.00 |
| 227.82 | 250.50 | 259.06 | 1.00 | 4.00 |
| 232.70 | 252.08 | 256.20 | 1.00 | 4.00 |
| 242.06 | 230.16 | 248.86 | 1.00 | 4.00 |
| 210.05 | 253.31 | 252.43 | 1.00 | 4.00 |
| 241.75 | 234.07 | 631.14 | 1.00 | 4.00 |
| 232.58 | 244.70 | 570.60 | 1.00 | 4.00 |
| 208.23 | 281.28 | 343.32 | 1.00 | 4.00 |
| 220.65 | 240.49 | 374.45 | 1.00 | 4.00 |
| 221.53 | 286.87 | 416.34 | 1.00 | 4.00 |
| 213.13 | 259.37 | 299.47 | 1.00 | 4.00 |
| 216.41 | 266.30 | 421.93 | 1.00 | 4.00 |
| 204.58 | 237.78 | 303.20 | 1.00 | 4.00 |
| 241.57 | 246.93 | 295.20 | 1.00 | 4.00 |
| 211.85 | 254.76 | 414.19 | 1.00 | 4.00 |
| 224.52 | 228.15 | 295.79 | 1.00 | 4.00 |
| 228.77 | 247.91 | 360.76 | 1.00 | 4.00 |
| 191.46 | 236.67 | 264.62 | 1.00 | 4.00 |
| 228.84 | 251.90 | 333.84 | 1.00 | 4.00 |
| 241.54 | 219.73 | 291.01 | 1.00 | 4.00 |
| 206.25 | 258.65 | 276.79 | 1.00 | 4.00 |
| 232.71 | 249.10 | 275.55 | 1.00 | 4.00 |
| 244.97 | 235.15 | 279.32 | 1.00 | 4.00 |
| 189.42 | 252.82 | 335.10 | 1.00 | 4.00 |
| 193.22 | 229.97 | 290.79 | 1.00 | 4.00 |
| 229.28 | 250.75 | 292.87 | 1.00 | 4.00 |
| 245.06 | 244.71 | 264.43 | 1.00 | 4.00 |
| 187.15 | 232.80 | 283.77 | 1.00 | 4.00 |
| 191.78 | 263.37 | 372.20 | 1.00 | 4.00 |
| 236.48 | 272.05 | 280.86 | 1.00 | 4.00 |
| 243.07 | 238.23 | 350.98 | 1.00 | 4.00 |
| 194.64 | 250.66 | 323.49 | 1.00 | 4.00 |
| 249.90 | 209.19 | 292.82 | 1.00 | 4.00 |
| 190.24 | 277.44 | 333.86 | 1.00 | 4.00 |
| 243.81 | 263.45 | 303.88 | 1.00 | 4.00 |
| 220.45 | 244.49 | 322.78 | 1.00 | 4.00 |
| 201.18 | 254.81 | 305.56 | 1.00 | 4.00 |

| | | | | |
|---|---|---|---|---|
| 184.29 | 248.93 | 342.89 | 1.00 | 4.00 |
| 210.74 | 221.15 | 351.83 | 1.00 | 4.00 |
| 242.81 | 269.98 | 276.07 | 1.00 | 4.00 |
| 211.88 | 244.88 | 324.74 | 1.00 | 4.00 |
| 230.05 | 247.49 | 358.71 | 1.00 | 4.00 |
| 189.09 | 256.64 | 326.18 | 1.00 | 4.00 |
| 210.46 | 267.79 | 336.16 | 1.00 | 4.00 |
| 230.84 | 228.52 | 298.42 | 1.00 | 4.00 |
| 200.61 | 271.96 | 304.53 | 1.00 | 4.00 |
| 216.95 | 254.63 | 362.55 | 1.00 | 4.00 |
| 234.46 | 223.24 | 383.57 | 1.00 | 4.00 |
| 193.36 | 247.36 | 366.60 | 1.00 | 4.00 |
| 221.63 | 266.24 | 309.20 | 1.00 | 4.00 |
| 198.53 | 224.73 | 265.71 | 1.00 | 4.00 |
| 211.97 | 267.40 | 318.29 | 1.00 | 4.00 |
| 223.27 | 269.74 | 343.37 | 1.00 | 4.00 |
| 196.11 | 210.21 | 340.00 | 1.00 | 4.00 |
| 174.77 | 237.67 | 337.42 | 1.00 | 4.00 |
| 210.85 | 266.04 | 320.46 | 1.00 | 4.00 |
| 208.02 | 255.08 | 295.67 | 1.00 | 4.00 |
| 221.36 | 254.83 | 291.28 | 1.00 | 4.00 |
| 203.97 | 291.34 | 380.11 | 1.00 | 4.00 |
| 167.72 | 235.37 | 344.97 | 1.00 | 4.00 |
| 204.59 | 247.45 | 322.41 | 1.00 | 4.00 |
| 215.37 | 255.08 | 344.15 | 1.00 | 4.00 |
| 251.34 | 258.73 | 319.38 | 1.00 | 4.00 |
| 207.57 | 248.47 | 308.92 | 1.00 | 4.00 |
| 190.83 | 287.48 | 372.87 | 1.00 | 4.00 |
| 203.73 | 245.37 | 391.79 | 1.00 | 4.00 |
| 213.64 | 244.26 | 327.05 | 1.00 | 4.00 |
| 226.38 | 250.80 | 371.76 | 1.00 | 4.00 |
| 213.07 | 253.49 | 299.46 | 1.00 | 4.00 |
| 199.82 | 253.20 | 304.77 | 1.00 | 4.00 |
| 190.68 | 264.93 | 316.55 | 1.00 | 4.00 |
| 232.20 | 241.40 | 379.26 | 1.00 | 4.00 |
| 213.72 | 236.81 | 307.23 | 1.00 | 4.00 |
| 194.70 | 238.51 | 440.97 | 1.00 | 4.00 |
| 189.42 | 245.22 | 268.65 | 1.00 | 4.00 |
| 210.99 | 273.77 | 303.11 | 1.00 | 4.00 |
| 214.97 | 248.51 | 304.84 | 1.00 | 4.00 |
| 200.42 | 226.40 | 312.26 | 1.00 | 4.00 |
| 224.66 | 256.46 | 348.16 | 1.00 | 4.00 |
| 204.55 | 242.78 | 316.31 | 1.00 | 4.00 |

| | | | | |
|---|---|---|---|---|
| 226.56 | 273.16 | 222.78 | 1.00 | 4.00 |
| 242.94 | 260.68 | 315.58 | 1.00 | 4.00 |
| 207.72 | 220.97 | 358.36 | 1.00 | 4.00 |
| 216.33 | 245.18 | 322.10 | 1.00 | 4.00 |
| 212.55 | 257.35 | 278.07 | 1.00 | 4.00 |
| 190.81 | 246.41 | 257.53 | 1.00 | 4.00 |
| 234.98 | 247.10 | 346.26 | 1.00 | 4.00 |
| 192.15 | 237.90 | 254.37 | 1.00 | 4.00 |
| 238.47 | 263.17 | 404.55 | 1.00 | 4.00 |
| 175.38 | 220.39 | 248.79 | 1.00 | 4.00 |
| 209.77 | 260.07 | 276.64 | 1.00 | 4.00 |
| 164.79 | 186.43 | 298.06 | 2.00 | 1.00 |
| 137.54 | 146.08 | 251.91 | 2.00 | 1.00 |
| 155.99 | 187.78 | 350.88 | 2.00 | 1.00 |
| 192.27 | 210.97 | 380.19 | 2.00 | 1.00 |
| 160.07 | 163.45 | 279.93 | 2.00 | 1.00 |
| 178.20 | 208.11 | 356.61 | 2.00 | 1.00 |
| 161.78 | 165.09 | 297.47 | 2.00 | 1.00 |
| 155.45 | 158.94 | 288.66 | 2.00 | 1.00 |
| 159.09 | 167.37 | 299.99 | 2.00 | 1.00 |
| 213.51 | 200.22 | 362.17 | 2.00 | 1.00 |
| 149.98 | 170.08 | 286.58 | 2.00 | 1.00 |
| 179.82 | 196.10 | 340.95 | 2.00 | 1.00 |
| 220.44 | 230.37 | 401.41 | 2.00 | 1.00 |
| 188.41 | 185.75 | 349.10 | 2.00 | 1.00 |
| 171.97 | 201.07 | 337.69 | 2.00 | 1.00 |
| 187.82 | 199.89 | 332.59 | 2.00 | 1.00 |
| 305.63 | 261.28 | 514.96 | 2.00 | 1.00 |
| 162.80 | 178.85 | 305.67 | 2.00 | 1.00 |
| 163.26 | 204.04 | 336.19 | 2.00 | 1.00 |
| 152.83 | 169.65 | 280.32 | 2.00 | 1.00 |
| 262.69 | 236.43 | 452.19 | 2.00 | 1.00 |
| 155.18 | 177.65 | 298.83 | 2.00 | 1.00 |
| 157.74 | 189.44 | 318.31 | 2.00 | 1.00 |
| 208.23 | 234.66 | 412.43 | 2.00 | 1.00 |
| 247.00 | 238.71 | 450.14 | 2.00 | 1.00 |
| 197.30 | 204.95 | 361.02 | 2.00 | 1.00 |
| 153.23 | 148.08 | 282.13 | 2.00 | 1.00 |
| 259.79 | 278.05 | 493.28 | 2.00 | 1.00 |
| 252.87 | 284.80 | 537.40 | 2.00 | 1.00 |
| 210.23 | 219.39 | 398.37 | 2.00 | 1.00 |
| 171.45 | 176.01 | 313.44 | 2.00 | 1.00 |
| 208.01 | 201.20 | 377.66 | 2.00 | 1.00 |

| | | | | |
|---|---|---|---|---|
| 282.50 | 297.31 | 521.56 | 2.00 | 1.00 |
| 276.48 | 327.45 | 580.13 | 2.00 | 1.00 |
| 204.06 | 206.48 | 374.31 | 2.00 | 1.00 |
| 202.34 | 212.11 | 362.78 | 2.00 | 1.00 |
| 253.73 | 289.63 | 481.30 | 2.00 | 1.00 |
| 261.25 | 271.72 | 475.55 | 2.00 | 1.00 |
| 263.77 | 279.55 | 482.43 | 2.00 | 1.00 |
| 179.00 | 206.29 | 376.37 | 2.00 | 1.00 |
| 237.43 | 254.70 | 398.94 | 2.00 | 1.00 |
| 241.53 | 244.85 | 413.90 | 2.00 | 1.00 |
| 235.37 | 273.63 | 433.44 | 2.00 | 1.00 |
| 347.55 | 336.89 | 607.51 | 2.00 | 1.00 |
| 241.36 | 238.00 | 435.30 | 2.00 | 1.00 |
| 229.37 | 252.61 | 466.81 | 2.00 | 1.00 |
| 202.71 | 220.50 | 323.50 | 2.00 | 1.00 |
| 276.42 | 295.13 | 472.52 | 2.00 | 1.00 |
| 305.42 | 309.22 | 535.65 | 2.00 | 1.00 |
| 291.17 | 296.06 | 520.73 | 2.00 | 1.00 |
| 224.90 | 226.84 | 414.28 | 2.00 | 1.00 |
| 254.49 | 278.66 | 489.39 | 2.00 | 1.00 |
| 163.39 | 210.13 | 307.54 | 2.00 | 1.00 |
| 300.01 | 326.55 | 529.22 | 2.00 | 1.00 |
| 282.58 | 313.13 | 487.61 | 2.00 | 1.00 |
| 282.36 | 302.62 | 512.53 | 2.00 | 1.00 |
| 186.04 | 192.22 | 352.19 | 2.00 | 1.00 |
| 293.47 | 292.60 | 528.17 | 2.00 | 1.00 |
| 189.43 | 201.01 | 343.87 | 2.00 | 1.00 |
| 275.83 | 321.71 | 495.86 | 2.00 | 1.00 |
| 227.69 | 243.43 | 406.23 | 2.00 | 1.00 |
| 219.48 | 261.95 | 419.18 | 2.00 | 1.00 |
| 334.99 | 364.80 | 579.18 | 2.00 | 1.00 |
| 183.85 | 150.63 | 283.21 | 2.00 | 1.00 |
| 238.98 | 276.51 | 468.99 | 2.00 | 1.00 |
| 246.18 | 247.89 | 425.75 | 2.00 | 1.00 |
| 292.39 | 305.32 | 512.70 | 2.00 | 1.00 |
| 187.60 | 223.56 | 352.90 | 2.00 | 1.00 |
| 279.72 | 318.21 | 483.29 | 2.00 | 1.00 |
| 198.80 | 209.75 | 352.26 | 2.00 | 1.00 |
| 177.47 | 183.02 | 333.64 | 2.00 | 1.00 |
| 242.25 | 238.86 | 417.38 | 2.00 | 1.00 |
| 270.13 | 270.58 | 462.14 | 2.00 | 1.00 |
| 197.85 | 220.28 | 361.21 | 2.00 | 1.00 |
| 202.10 | 219.85 | 354.80 | 2.00 | 1.00 |

| | | | | |
|---|---|---|---|---|
| 221.29 | 242.00 | 390.84 | 2.00 | 1.00 |
| 154.20 | 142.88 | 256.05 | 2.00 | 1.00 |
| 207.26 | 230.28 | 381.75 | 2.00 | 1.00 |
| 217.89 | 213.05 | 396.32 | 2.00 | 1.00 |
| 249.98 | 288.85 | 464.94 | 2.00 | 1.00 |
| 284.48 | 321.22 | 503.65 | 2.00 | 1.00 |
| 197.03 | 210.75 | 350.79 | 2.00 | 1.00 |
| 157.93 | 168.95 | 278.54 | 2.00 | 1.00 |
| 182.43 | 183.46 | 356.25 | 2.00 | 1.00 |
| 206.19 | 242.10 | 390.45 | 2.00 | 1.00 |
| 287.88 | 339.25 | 538.12 | 2.00 | 1.00 |
| 207.06 | 214.18 | 349.93 | 2.00 | 1.00 |
| 196.03 | 221.87 | 369.93 | 2.00 | 1.00 |
| 158.57 | 167.01 | 317.63 | 2.00 | 1.00 |
| 230.36 | 254.84 | 435.49 | 2.00 | 1.00 |
| 289.72 | 314.83 | 528.61 | 2.00 | 1.00 |
| 273.62 | 301.61 | 510.32 | 2.00 | 1.00 |
| 174.10 | 210.60 | 353.77 | 2.00 | 1.00 |
| 192.41 | 216.50 | 381.68 | 2.00 | 1.00 |
| 239.72 | 263.11 | 459.90 | 2.00 | 1.00 |
| 339.17 | 416.43 | 701.14 | 2.00 | 1.00 |
| 279.93 | 322.06 | 524.96 | 2.00 | 1.00 |
| 240.31 | 259.92 | 455.49 | 2.00 | 1.00 |
| 206.53 | 240.04 | 417.89 | 2.00 | 1.00 |
| 285.83 | 312.86 | 544.56 | 2.00 | 1.00 |
| 234.33 | 290.75 | 508.87 | 2.00 | 1.00 |
| 279.57 | 336.98 | 588.01 | 2.00 | 1.00 |
| 227.39 | 263.98 | 459.66 | 2.00 | 1.00 |
| 183.91 | 206.23 | 358.51 | 2.00 | 1.00 |
| 202.13 | 250.26 | 435.88 | 2.00 | 1.00 |
| 326.07 | 367.54 | 671.96 | 2.00 | 1.00 |
| 195.74 | 209.12 | 365.02 | 2.00 | 1.00 |
| 156.23 | 172.75 | 326.08 | 2.00 | 1.00 |
| 537.33 | 562.99 | 1042.18 | 2.00 | 1.00 |
| 207.17 | 222.06 | 406.83 | 2.00 | 1.00 |
| 182.61 | 200.76 | 356.85 | 2.00 | 1.00 |
| 366.34 | 383.89 | 653.82 | 2.00 | 1.00 |
| 305.42 | 332.24 | 649.05 | 2.00 | 1.00 |
| 331.97 | 308.23 | 676.52 | 2.00 | 1.00 |
| 201.20 | 235.89 | 448.76 | 2.00 | 1.00 |
| 267.18 | 217.69 | 527.04 | 2.00 | 1.00 |
| 222.15 | 260.76 | 490.51 | 2.00 | 1.00 |
| 248.34 | 236.90 | 469.94 | 2.00 | 1.00 |

| | | | | |
|---|---|---|---|---|
| 201.89 | 230.41 | 488.67 | 2.00 | 1.00 |
| 301.58 | 330.13 | 593.95 | 2.00 | 1.00 |
| 256.14 | 258.58 | 498.14 | 2.00 | 1.00 |
| 246.15 | 305.30 | 622.43 | 2.00 | 1.00 |
| 285.74 | 275.99 | 596.29 | 2.00 | 1.00 |
| 268.07 | 290.44 | 550.21 | 2.00 | 1.00 |
| 231.04 | 334.27 | 628.41 | 2.00 | 1.00 |
| 294.35 | 354.17 | 624.34 | 2.00 | 1.00 |
| 422.34 | 385.66 | 853.64 | 2.00 | 1.00 |
| 400.47 | 528.30 | 1018.87 | 2.00 | 1.00 |
| 207.46 | 290.15 | 560.94 | 2.00 | 1.00 |
| 355.26 | 446.50 | 810.36 | 2.00 | 1.00 |
| 212.45 | 206.96 | 469.21 | 2.00 | 1.00 |
| 239.43 | 388.19 | 704.30 | 2.00 | 1.00 |
| 228.60 | 284.25 | 539.08 | 2.00 | 1.00 |
| 336.97 | 415.24 | 788.76 | 2.00 | 1.00 |
| 249.31 | 263.13 | 490.17 | 2.00 | 1.00 |
| 185.86 | 250.86 | 528.00 | 2.00 | 1.00 |
| 193.07 | 236.82 | 467.93 | 2.00 | 1.00 |
| 326.41 | 432.92 | 809.54 | 2.00 | 1.00 |
| 310.91 | 397.40 | 746.76 | 2.00 | 1.00 |
| 322.63 | 376.62 | 704.18 | 2.00 | 1.00 |
| 201.19 | 266.69 | 523.51 | 2.00 | 1.00 |
| 172.92 | 231.19 | 439.46 | 2.00 | 1.00 |
| 217.10 | 290.55 | 558.47 | 2.00 | 1.00 |
| 287.29 | 347.10 | 629.18 | 2.00 | 1.00 |
| 321.71 | 395.83 | 743.58 | 2.00 | 1.00 |
| 136.52 | 188.69 | 344.71 | 2.00 | 1.00 |
| 220.89 | 295.09 | 569.99 | 2.00 | 1.00 |
| 249.11 | 283.89 | 527.72 | 2.00 | 1.00 |
| 243.65 | 319.66 | 541.38 | 2.00 | 1.00 |
| 290.63 | 353.24 | 660.24 | 2.00 | 1.00 |
| 180.23 | 227.77 | 409.54 | 2.00 | 1.00 |
| 161.10 | 173.30 | 330.10 | 2.00 | 1.00 |
| 284.12 | 331.36 | 627.79 | 2.00 | 1.00 |
| 224.05 | 265.83 | 537.40 | 2.00 | 1.00 |
| 213.37 | 285.11 | 509.93 | 2.00 | 1.00 |
| 288.12 | 411.33 | 671.20 | 2.00 | 1.00 |
| 227.86 | 271.72 | 502.75 | 2.00 | 1.00 |
| 150.25 | 202.99 | 353.60 | 2.00 | 1.00 |
| 315.46 | 312.13 | 617.17 | 2.00 | 1.00 |
| 234.69 | 270.28 | 519.93 | 2.00 | 1.00 |
| 183.99 | 221.95 | 422.76 | 2.00 | 1.00 |

| | | | | |
|---|---|---|---|---|
| 371.79 | 414.43 | 822.24 | 2.00 | 1.00 |
| 380.11 | 580.24 | 912.66 | 2.00 | 1.00 |
| 202.32 | 282.38 | 509.63 | 2.00 | 1.00 |
| 250.90 | 286.38 | 520.81 | 2.00 | 1.00 |
| 240.35 | 265.25 | 523.70 | 2.00 | 1.00 |
| 277.33 | 319.87 | 625.25 | 2.00 | 1.00 |
| 303.05 | 430.59 | 760.53 | 2.00 | 1.00 |
| 462.32 | 559.09 | 1043.31 | 2.00 | 1.00 |
| 236.48 | 344.18 | 567.33 | 2.00 | 1.00 |
| 232.46 | 298.28 | 487.08 | 2.00 | 1.00 |
| 326.34 | 401.01 | 735.60 | 2.00 | 1.00 |
| 193.03 | 216.55 | 368.80 | 2.00 | 2.00 |
| 228.95 | 240.03 | 445.64 | 2.00 | 2.00 |
| 190.88 | 203.49 | 398.00 | 2.00 | 2.00 |
| 288.60 | 309.88 | 556.46 | 2.00 | 2.00 |
| 203.69 | 179.68 | 375.56 | 2.00 | 2.00 |
| 179.23 | 170.69 | 335.49 | 2.00 | 2.00 |
| 212.84 | 202.77 | 391.88 | 2.00 | 2.00 |
| 214.00 | 183.73 | 392.96 | 2.00 | 2.00 |
| 159.22 | 179.39 | 316.62 | 2.00 | 2.00 |
| 188.79 | 212.99 | 391.47 | 2.00 | 2.00 |
| 204.35 | 242.67 | 442.70 | 2.00 | 2.00 |
| 198.72 | 197.99 | 383.42 | 2.00 | 2.00 |
| 269.02 | 264.18 | 546.89 | 2.00 | 2.00 |
| 208.25 | 239.36 | 459.90 | 2.00 | 2.00 |
| 180.94 | 183.61 | 349.44 | 2.00 | 2.00 |
| 247.06 | 221.66 | 457.66 | 2.00 | 2.00 |
| 230.47 | 206.36 | 464.50 | 2.00 | 2.00 |
| 166.11 | 183.30 | 350.22 | 2.00 | 2.00 |
| 220.02 | 219.35 | 423.36 | 2.00 | 2.00 |
| 218.19 | 175.29 | 386.37 | 2.00 | 2.00 |
| 280.13 | 248.88 | 554.52 | 2.00 | 2.00 |
| 188.97 | 208.56 | 386.51 | 2.00 | 2.00 |
| 208.78 | 216.57 | 426.76 | 2.00 | 2.00 |
| 198.50 | 189.71 | 378.90 | 2.00 | 2.00 |
| 309.26 | 243.82 | 554.18 | 2.00 | 2.00 |
| 216.96 | 240.52 | 433.78 | 2.00 | 2.00 |
| 166.75 | 199.40 | 367.58 | 2.00 | 2.00 |
| 237.64 | 225.25 | 480.90 | 2.00 | 2.00 |
| 274.12 | 258.21 | 560.75 | 2.00 | 2.00 |
| 234.30 | 224.23 | 480.07 | 2.00 | 2.00 |
| 269.45 | 251.86 | 475.40 | 2.00 | 2.00 |
| 274.12 | 291.14 | 610.95 | 2.00 | 2.00 |

| | | | | |
|---|---|---|---|---|
| 202.76 | 213.12 | 393.83 | 2.00 | 2.00 |
| 258.82 | 263.67 | 549.30 | 2.00 | 2.00 |
| 199.51 | 199.17 | 412.26 | 2.00 | 2.00 |
| 244.21 | 270.63 | 508.74 | 2.00 | 2.00 |
| 344.37 | 285.54 | 589.48 | 2.00 | 2.00 |
| 205.34 | 235.22 | 450.58 | 2.00 | 2.00 |
| 163.08 | 157.93 | 334.83 | 2.00 | 2.00 |
| 166.17 | 162.68 | 341.29 | 2.00 | 2.00 |
| 186.60 | 228.14 | 436.48 | 2.00 | 2.00 |
| 243.86 | 245.12 | 504.69 | 2.00 | 2.00 |
| 195.40 | 178.73 | 359.99 | 2.00 | 2.00 |
| 292.24 | 250.49 | 542.16 | 2.00 | 2.00 |
| 160.38 | 157.12 | 334.63 | 2.00 | 2.00 |
| 179.48 | 161.20 | 339.91 | 2.00 | 2.00 |
| 225.84 | 233.28 | 472.26 | 2.00 | 2.00 |
| 222.63 | 238.16 | 485.88 | 2.00 | 2.00 |
| 234.13 | 211.58 | 461.52 | 2.00 | 2.00 |
| 223.33 | 182.95 | 409.83 | 2.00 | 2.00 |
| 193.20 | 169.88 | 350.83 | 2.00 | 2.00 |
| 163.51 | 150.03 | 322.96 | 2.00 | 2.00 |
| 226.20 | 197.22 | 413.56 | 2.00 | 2.00 |
| 238.57 | 245.05 | 503.21 | 2.00 | 2.00 |
| 254.96 | 245.51 | 519.96 | 2.00 | 2.00 |
| 513.67 | 527.68 | 1115.60 | 2.00 | 2.00 |
| 224.95 | 187.57 | 410.16 | 2.00 | 2.00 |
| 166.22 | 141.94 | 308.78 | 2.00 | 2.00 |
| 174.63 | 164.67 | 337.50 | 2.00 | 2.00 |
| 301.81 | 242.05 | 526.46 | 2.00 | 2.00 |
| 223.00 | 238.84 | 465.00 | 2.00 | 2.00 |
| 315.12 | 304.54 | 615.60 | 2.00 | 2.00 |
| 511.12 | 528.11 | 1067.79 | 2.00 | 2.00 |
| 200.80 | 184.64 | 387.60 | 2.00 | 2.00 |
| 130.66 | 136.91 | 288.38 | 2.00 | 2.00 |
| 261.91 | 187.15 | 431.73 | 2.00 | 2.00 |
| 189.99 | 211.44 | 408.78 | 2.00 | 2.00 |
| 235.08 | 229.60 | 443.59 | 2.00 | 2.00 |
| 290.71 | 309.71 | 615.19 | 2.00 | 2.00 |
| 314.44 | 309.62 | 631.76 | 2.00 | 2.00 |
| 158.20 | 168.55 | 367.06 | 2.00 | 2.00 |
| 190.53 | 165.24 | 310.19 | 2.00 | 2.00 |
| 168.29 | 187.41 | 386.08 | 2.00 | 2.00 |
| 209.56 | 238.78 | 439.66 | 2.00 | 2.00 |
| 177.29 | 184.39 | 374.60 | 2.00 | 2.00 |

| | | | | |
|---|---|---|---|---|
| 271.09 | 318.93 | 629.74 | 2.00 | 2.00 |
| 362.19 | 351.74 | 702.77 | 2.00 | 2.00 |
| 171.87 | 168.47 | 356.88 | 2.00 | 2.00 |
| 158.90 | 166.69 | 317.94 | 2.00 | 2.00 |
| 180.15 | 186.85 | 367.24 | 2.00 | 2.00 |
| 250.60 | 283.17 | 537.74 | 2.00 | 2.00 |
| 151.84 | 179.07 | 363.60 | 2.00 | 2.00 |
| 275.63 | 284.84 | 514.52 | 2.00 | 2.00 |
| 270.55 | 234.70 | 503.83 | 2.00 | 2.00 |
| 221.19 | 235.19 | 414.42 | 2.00 | 2.00 |
| 172.74 | 226.85 | 418.01 | 2.00 | 2.00 |
| 142.61 | 178.22 | 333.02 | 2.00 | 2.00 |
| 253.77 | 258.21 | 504.29 | 2.00 | 2.00 |
| 141.90 | 171.19 | 361.04 | 2.00 | 2.00 |
| 139.21 | 164.17 | 358.34 | 2.00 | 2.00 |
| 186.86 | 210.68 | 451.19 | 2.00 | 2.00 |
| 132.56 | 150.30 | 301.67 | 2.00 | 2.00 |
| 151.98 | 251.58 | 467.62 | 2.00 | 2.00 |
| 128.61 | 171.25 | 319.32 | 2.00 | 2.00 |
| 167.47 | 238.07 | 439.18 | 2.00 | 2.00 |
| 171.75 | 258.79 | 489.16 | 2.00 | 2.00 |
| 141.22 | 160.81 | 318.44 | 2.00 | 2.00 |
| 128.44 | 194.38 | 369.65 | 2.00 | 2.00 |
| 143.78 | 206.07 | 407.98 | 2.00 | 2.00 |
| 141.22 | 174.36 | 317.12 | 2.00 | 2.00 |
| 152.32 | 179.97 | 346.81 | 2.00 | 2.00 |
| 121.96 | 171.50 | 353.59 | 2.00 | 2.00 |
| 180.00 | 218.22 | 394.43 | 2.00 | 2.00 |
| 148.05 | 179.00 | 316.40 | 2.00 | 2.00 |
| 115.68 | 135.94 | 257.69 | 2.00 | 2.00 |
| 125.17 | 167.26 | 313.59 | 2.00 | 2.00 |
| 167.11 | 244.73 | 457.09 | 2.00 | 2.00 |
| 121.59 | 177.11 | 302.15 | 2.00 | 2.00 |
| 126.65 | 153.21 | 310.25 | 2.00 | 2.00 |
| 227.26 | 285.64 | 562.91 | 2.00 | 2.00 |
| 205.64 | 283.56 | 576.59 | 2.00 | 2.00 |
| 124.81 | 163.34 | 298.06 | 2.00 | 2.00 |
| 161.76 | 222.18 | 434.42 | 2.00 | 2.00 |
| 156.44 | 214.53 | 386.41 | 2.00 | 2.00 |
| 229.92 | 265.85 | 506.08 | 2.00 | 2.00 |
| 144.23 | 182.80 | 330.94 | 2.00 | 2.00 |
| 132.04 | 162.39 | 330.92 | 2.00 | 2.00 |
| 184.69 | 221.77 | 451.24 | 2.00 | 2.00 |

| | | | | |
|---|---|---|---|---|
| 134.19 | 187.59 | 336.90 | 2.00 | 2.00 |
| 177.21 | 237.84 | 425.89 | 2.00 | 2.00 |
| 161.64 | 201.37 | 381.70 | 2.00 | 2.00 |
| 149.39 | 165.98 | 338.64 | 2.00 | 2.00 |
| 270.74 | 295.60 | 604.59 | 2.00 | 2.00 |
| 173.91 | 250.20 | 393.92 | 2.00 | 2.00 |
| 180.88 | 237.78 | 432.50 | 2.00 | 2.00 |
| 145.97 | 212.20 | 397.55 | 2.00 | 2.00 |
| 160.37 | 188.53 | 363.40 | 2.00 | 2.00 |
| 219.21 | 224.60 | 420.53 | 2.00 | 2.00 |
| 250.03 | 240.47 | 535.50 | 2.00 | 2.00 |
| 167.70 | 188.00 | 343.19 | 2.00 | 2.00 |
| 198.07 | 229.36 | 472.76 | 2.00 | 2.00 |
| 208.15 | 300.51 | 587.67 | 2.00 | 2.00 |
| 150.66 | 212.39 | 411.93 | 2.00 | 2.00 |
| 188.02 | 194.97 | 431.29 | 2.00 | 2.00 |
| 268.95 | 242.70 | 506.07 | 2.00 | 2.00 |
| 210.40 | 211.10 | 418.35 | 2.00 | 2.00 |
| 212.35 | 247.38 | 517.25 | 2.00 | 2.00 |
| 323.59 | 484.85 | 860.20 | 2.00 | 2.00 |
| 219.89 | 262.10 | 517.00 | 2.00 | 2.00 |
| 158.54 | 219.71 | 452.80 | 2.00 | 2.00 |
| 187.82 | 241.62 | 442.32 | 2.00 | 2.00 |
| 165.59 | 175.04 | 351.28 | 2.00 | 2.00 |
| 263.83 | 323.39 | 570.00 | 2.00 | 2.00 |
| 204.76 | 254.63 | 497.09 | 2.00 | 2.00 |
| 226.26 | 267.08 | 579.64 | 2.00 | 2.00 |
| 265.43 | 268.02 | 520.41 | 2.00 | 2.00 |
| 221.61 | 239.91 | 482.29 | 2.00 | 2.00 |
| 174.85 | 261.45 | 450.22 | 2.00 | 2.00 |
| 168.73 | 193.03 | 360.33 | 2.00 | 2.00 |
| 283.44 | 302.17 | 663.68 | 2.00 | 2.00 |
| 255.65 | 280.56 | 548.76 | 2.00 | 2.00 |
| 224.84 | 322.25 | 562.61 | 2.00 | 2.00 |
| 252.03 | 367.72 | 645.20 | 2.00 | 2.00 |
| 234.86 | 250.67 | 491.90 | 2.00 | 2.00 |
| 111.65 | 162.75 | 303.75 | 2.00 | 2.00 |
| 223.35 | 248.08 | 475.18 | 2.00 | 2.00 |
| 184.83 | 245.43 | 457.43 | 2.00 | 2.00 |
| 257.55 | 266.03 | 560.11 | 2.00 | 2.00 |
| 247.25 | 328.59 | 595.59 | 2.00 | 2.00 |
| 187.74 | 246.63 | 464.69 | 2.00 | 2.00 |
| 165.96 | 191.94 | 392.59 | 2.00 | 2.00 |

| | | | | |
|---|---|---|---|---|
| 212.67 | 240.53 | 420.95 | 2.00 | 2.00 |
| 205.59 | 230.57 | 477.01 | 2.00 | 2.00 |
| 192.23 | 216.60 | 440.51 | 2.00 | 2.00 |
| 181.44 | 228.79 | 423.66 | 2.00 | 2.00 |
| 155.71 | 197.41 | 409.74 | 2.00 | 2.00 |
| 207.39 | 243.08 | 444.10 | 2.00 | 2.00 |
| 170.00 | 214.06 | 415.15 | 2.00 | 2.00 |
| 167.59 | 200.64 | 363.54 | 2.00 | 2.00 |
| 216.74 | 190.92 | 387.67 | 2.00 | 3.00 |
| 191.85 | 182.62 | 354.38 | 2.00 | 3.00 |
| 184.96 | 185.48 | 340.48 | 2.00 | 3.00 |
| 191.83 | 215.65 | 423.75 | 2.00 | 3.00 |
| 200.44 | 208.19 | 372.03 | 2.00 | 3.00 |
| 193.83 | 220.62 | 426.79 | 2.00 | 3.00 |
| 188.13 | 196.34 | 342.20 | 2.00 | 3.00 |
| 146.49 | 166.85 | 348.66 | 2.00 | 3.00 |
| 188.85 | 190.20 | 400.85 | 2.00 | 3.00 |
| 184.77 | 180.38 | 351.73 | 2.00 | 3.00 |
| 129.81 | 156.41 | 336.96 | 2.00 | 3.00 |
| 177.19 | 186.63 | 367.16 | 2.00 | 3.00 |
| 182.26 | 171.12 | 364.99 | 2.00 | 3.00 |
| 133.46 | 154.75 | 341.21 | 2.00 | 3.00 |
| 147.45 | 165.75 | 339.30 | 2.00 | 3.00 |
| 170.34 | 188.24 | 374.75 | 2.00 | 3.00 |
| 169.29 | 161.18 | 342.34 | 2.00 | 3.00 |
| 185.32 | 217.37 | 425.61 | 2.00 | 3.00 |
| 164.86 | 177.71 | 358.68 | 2.00 | 3.00 |
| 139.13 | 160.58 | 316.27 | 2.00 | 3.00 |
| 137.64 | 152.75 | 285.35 | 2.00 | 3.00 |
| 167.32 | 196.88 | 363.90 | 2.00 | 3.00 |
| 177.17 | 163.85 | 336.71 | 2.00 | 3.00 |
| 156.26 | 160.73 | 307.38 | 2.00 | 3.00 |
| 131.00 | 164.04 | 318.16 | 2.00 | 3.00 |
| 143.98 | 174.96 | 322.61 | 2.00 | 3.00 |
| 151.73 | 152.28 | 302.38 | 2.00 | 3.00 |
| 195.92 | 190.34 | 382.13 | 2.00 | 3.00 |
| 153.55 | 157.63 | 312.25 | 2.00 | 3.00 |
| 194.21 | 202.07 | 415.46 | 2.00 | 3.00 |
| 140.38 | 177.00 | 317.04 | 2.00 | 3.00 |
| 143.71 | 140.08 | 287.67 | 2.00 | 3.00 |
| 189.08 | 192.26 | 385.43 | 2.00 | 3.00 |
| 273.78 | 256.78 | 507.73 | 2.00 | 3.00 |
| 159.58 | 154.15 | 319.17 | 2.00 | 3.00 |

| | | | | |
|---|---|---|---|---|
| 211.64 | 221.71 | 422.79 | 2.00 | 3.00 |
| 165.86 | 163.07 | 344.70 | 2.00 | 3.00 |
| 170.01 | 157.20 | 321.44 | 2.00 | 3.00 |
| 191.20 | 190.41 | 387.46 | 2.00 | 3.00 |
| 272.94 | 278.60 | 535.60 | 2.00 | 3.00 |
| 157.04 | 193.16 | 366.49 | 2.00 | 3.00 |
| 222.97 | 241.23 | 493.24 | 2.00 | 3.00 |
| 158.81 | 177.45 | 357.13 | 2.00 | 3.00 |
| 173.52 | 189.30 | 358.82 | 2.00 | 3.00 |
| 206.77 | 190.58 | 425.29 | 2.00 | 3.00 |
| 190.45 | 195.58 | 385.84 | 2.00 | 3.00 |
| 293.90 | 301.15 | 561.36 | 2.00 | 3.00 |
| 137.26 | 157.56 | 330.87 | 2.00 | 3.00 |
| 231.56 | 274.05 | 504.44 | 2.00 | 3.00 |
| 224.86 | 232.18 | 437.86 | 2.00 | 3.00 |
| 185.28 | 200.63 | 412.76 | 2.00 | 3.00 |
| 225.10 | 195.69 | 457.55 | 2.00 | 3.00 |
| 220.67 | 229.27 | 429.67 | 2.00 | 3.00 |
| 260.53 | 219.23 | 498.59 | 2.00 | 3.00 |
| 165.51 | 210.16 | 401.83 | 2.00 | 3.00 |
| 153.47 | 218.64 | 393.07 | 2.00 | 3.00 |
| 197.58 | 215.84 | 421.45 | 2.00 | 3.00 |
| 243.04 | 231.20 | 463.31 | 2.00 | 3.00 |
| 244.15 | 195.38 | 433.42 | 2.00 | 3.00 |
| 174.79 | 192.16 | 345.32 | 2.00 | 3.00 |
| 226.71 | 196.47 | 468.61 | 2.00 | 3.00 |
| 281.77 | 263.39 | 561.97 | 2.00 | 3.00 |
| 136.99 | 181.26 | 346.47 | 2.00 | 3.00 |
| 180.39 | 215.46 | 428.82 | 2.00 | 3.00 |
| 185.90 | 184.48 | 374.51 | 2.00 | 3.00 |
| 205.40 | 189.75 | 391.27 | 2.00 | 3.00 |
| 229.20 | 251.13 | 434.26 | 2.00 | 3.00 |
| 176.97 | 162.80 | 330.35 | 2.00 | 3.00 |
| 316.28 | 287.67 | 614.51 | 2.00 | 3.00 |
| 153.60 | 175.30 | 348.43 | 2.00 | 3.00 |
| 148.18 | 177.74 | 351.44 | 2.00 | 3.00 |
| 183.51 | 208.44 | 430.46 | 2.00 | 3.00 |
| 223.40 | 217.22 | 426.84 | 2.00 | 3.00 |
| 194.00 | 202.66 | 385.17 | 2.00 | 3.00 |
| 154.89 | 139.62 | 318.19 | 2.00 | 3.00 |
| 231.54 | 219.04 | 463.18 | 2.00 | 3.00 |
| 152.61 | 172.14 | 335.62 | 2.00 | 3.00 |
| 154.76 | 212.79 | 399.25 | 2.00 | 3.00 |

| | | | | |
|---|---|---|---|---|
| 189.20 | 166.86 | 397.90 | 2.00 | 3.00 |
| 253.48 | 283.69 | 509.89 | 2.00 | 3.00 |
| 166.89 | 165.62 | 359.98 | 2.00 | 3.00 |
| 202.24 | 214.12 | 423.74 | 2.00 | 3.00 |
| 221.46 | 189.13 | 402.53 | 2.00 | 3.00 |
| 178.75 | 216.12 | 388.92 | 2.00 | 3.00 |
| 147.51 | 160.62 | 351.99 | 2.00 | 3.00 |
| 210.76 | 219.03 | 414.94 | 2.00 | 3.00 |
| 244.46 | 239.34 | 481.60 | 2.00 | 3.00 |
| 155.93 | 177.60 | 320.64 | 2.00 | 3.00 |
| 181.19 | 159.28 | 342.37 | 2.00 | 3.00 |
| 169.92 | 187.29 | 342.94 | 2.00 | 3.00 |
| 171.49 | 210.49 | 382.98 | 2.00 | 3.00 |
| 221.23 | 249.35 | 466.27 | 2.00 | 3.00 |
| 228.18 | 276.00 | 513.64 | 2.00 | 3.00 |
| 138.98 | 152.23 | 314.40 | 2.00 | 3.00 |
| 182.66 | 181.46 | 365.32 | 2.00 | 3.00 |
| 190.08 | 205.61 | 344.02 | 2.00 | 3.00 |
| 165.79 | 160.39 | 348.32 | 2.00 | 3.00 |
| 199.18 | 207.59 | 399.61 | 2.00 | 3.00 |
| 185.20 | 228.97 | 434.95 | 2.00 | 3.00 |
| 158.65 | 197.84 | 362.05 | 2.00 | 3.00 |
| 159.75 | 161.71 | 322.27 | 2.00 | 3.00 |
| 200.05 | 181.88 | 374.87 | 2.00 | 3.00 |
| 169.93 | 183.39 | 344.22 | 2.00 | 3.00 |
| 223.43 | 205.75 | 398.91 | 2.00 | 3.00 |
| 151.82 | 164.63 | 323.01 | 2.00 | 3.00 |
| 195.26 | 181.87 | 368.74 | 2.00 | 3.00 |
| 185.03 | 177.47 | 348.43 | 2.00 | 3.00 |
| 167.96 | 182.13 | 362.92 | 2.00 | 3.00 |
| 213.48 | 186.35 | 379.88 | 2.00 | 3.00 |
| 134.20 | 216.80 | 343.07 | 2.00 | 3.00 |
| 147.00 | 225.92 | 410.25 | 2.00 | 3.00 |
| 133.26 | 197.00 | 321.58 | 2.00 | 3.00 |
| 176.49 | 220.90 | 413.28 | 2.00 | 3.00 |
| 146.76 | 211.56 | 374.82 | 2.00 | 3.00 |
| 172.17 | 234.56 | 401.91 | 2.00 | 3.00 |
| 149.27 | 234.46 | 396.46 | 2.00 | 3.00 |
| 165.02 | 232.35 | 434.48 | 2.00 | 3.00 |
| 144.04 | 199.94 | 355.67 | 2.00 | 3.00 |
| 171.13 | 269.15 | 436.54 | 2.00 | 3.00 |
| 173.13 | 226.39 | 437.61 | 2.00 | 3.00 |
| 166.39 | 222.22 | 398.27 | 2.00 | 3.00 |

| | | | | |
|---|---|---|---|---|
| 171.46 | 229.08 | 417.71 | 2.00 | 3.00 |
| 241.68 | 279.85 | 513.51 | 2.00 | 3.00 |
| 192.36 | 246.39 | 491.77 | 2.00 | 3.00 |
| 160.40 | 231.00 | 401.49 | 2.00 | 3.00 |
| 144.88 | 209.04 | 360.52 | 2.00 | 3.00 |
| 199.29 | 224.63 | 423.71 | 2.00 | 3.00 |
| 187.47 | 255.34 | 444.72 | 2.00 | 3.00 |
| 217.05 | 234.57 | 449.27 | 2.00 | 3.00 |
| 147.41 | 217.21 | 360.17 | 2.00 | 3.00 |
| 222.61 | 301.30 | 487.86 | 2.00 | 3.00 |
| 167.41 | 246.22 | 411.57 | 2.00 | 3.00 |
| 224.97 | 263.28 | 468.93 | 2.00 | 3.00 |
| 144.57 | 191.14 | 346.49 | 2.00 | 3.00 |
| 193.82 | 230.11 | 416.19 | 2.00 | 3.00 |
| 186.55 | 274.57 | 453.56 | 2.00 | 3.00 |
| 190.69 | 228.68 | 437.96 | 2.00 | 3.00 |
| 213.88 | 264.19 | 439.15 | 2.00 | 3.00 |
| 163.40 | 191.92 | 335.30 | 2.00 | 3.00 |
| 190.68 | 223.39 | 398.65 | 2.00 | 3.00 |
| 178.19 | 234.03 | 406.53 | 2.00 | 3.00 |
| 242.33 | 297.75 | 510.09 | 2.00 | 3.00 |
| 185.41 | 215.30 | 379.24 | 2.00 | 3.00 |
| 124.75 | 166.14 | 278.10 | 2.00 | 3.00 |
| 149.19 | 190.24 | 335.01 | 2.00 | 3.00 |
| 209.05 | 262.10 | 454.25 | 2.00 | 3.00 |
| 178.67 | 243.55 | 429.94 | 2.00 | 3.00 |
| 236.53 | 284.20 | 502.84 | 2.00 | 3.00 |
| 164.27 | 188.55 | 343.44 | 2.00 | 3.00 |
| 115.36 | 176.40 | 280.22 | 2.00 | 3.00 |
| 143.49 | 200.18 | 370.95 | 2.00 | 3.00 |
| 142.08 | 191.40 | 333.68 | 2.00 | 3.00 |
| 171.10 | 249.51 | 446.51 | 2.00 | 3.00 |
| 155.12 | 209.69 | 341.00 | 2.00 | 3.00 |
| 156.75 | 188.59 | 326.81 | 2.00 | 3.00 |
| 121.34 | 174.04 | 293.86 | 2.00 | 3.00 |
| 124.23 | 186.85 | 344.01 | 2.00 | 3.00 |
| 164.94 | 222.12 | 398.72 | 2.00 | 3.00 |
| 140.91 | 186.10 | 344.43 | 2.00 | 3.00 |
| 137.46 | 177.58 | 291.82 | 2.00 | 3.00 |
| 140.83 | 176.82 | 303.80 | 2.00 | 3.00 |
| 146.18 | 246.38 | 399.07 | 2.00 | 3.00 |
| 123.72 | 184.65 | 324.97 | 2.00 | 3.00 |
| 139.95 | 179.17 | 329.21 | 2.00 | 3.00 |

| | | | | |
|---|---|---|---|---|
| 135.47 | 167.29 | 303.33 | 2.00 | 3.00 |
| 165.17 | 206.30 | 355.78 | 2.00 | 3.00 |
| 171.95 | 215.55 | 388.36 | 2.00 | 3.00 |
| 113.96 | 202.97 | 320.37 | 2.00 | 3.00 |
| 133.57 | 188.00 | 343.03 | 2.00 | 3.00 |
| 138.82 | 189.67 | 314.96 | 2.00 | 3.00 |
| 148.68 | 207.52 | 367.53 | 2.00 | 3.00 |
| 165.72 | 208.03 | 364.15 | 2.00 | 3.00 |
| 129.32 | 172.55 | 305.44 | 2.00 | 3.00 |
| 137.39 | 187.12 | 348.60 | 2.00 | 3.00 |
| 157.76 | 202.78 | 369.17 | 2.00 | 3.00 |
| 136.14 | 183.44 | 324.19 | 2.00 | 3.00 |
| 147.50 | 182.79 | 306.26 | 2.00 | 3.00 |
| 144.50 | 201.24 | 349.80 | 2.00 | 3.00 |
| 153.52 | 187.16 | 348.69 | 2.00 | 3.00 |
| 180.27 | 205.43 | 364.36 | 2.00 | 3.00 |
| 151.61 | 178.70 | 330.11 | 2.00 | 3.00 |
| 170.65 | 208.57 | 369.79 | 2.00 | 3.00 |
| 243.39 | 284.45 | 500.35 | 2.00 | 3.00 |
| 188.51 | 220.72 | 383.42 | 2.00 | 3.00 |
| 141.99 | 173.41 | 297.83 | 2.00 | 3.00 |
| 236.60 | 318.63 | 558.95 | 2.00 | 3.00 |
| 178.78 | 213.27 | 378.03 | 2.00 | 3.00 |
| 247.31 | 310.13 | 531.94 | 2.00 | 3.00 |
| 184.04 | 243.00 | 423.82 | 2.00 | 3.00 |
| 172.14 | 229.67 | 402.91 | 2.00 | 3.00 |
| 213.72 | 291.81 | 512.96 | 2.00 | 3.00 |
| 183.21 | 230.13 | 406.09 | 2.00 | 3.00 |
| 163.56 | 223.24 | 413.98 | 2.00 | 3.00 |
| 186.98 | 215.57 | 412.14 | 2.00 | 3.00 |
| 180.60 | 244.84 | 454.08 | 2.00 | 3.00 |
| 218.78 | 209.48 | 226.64 | 2.00 | 4.00 |
| 219.16 | 207.27 | 244.53 | 2.00 | 4.00 |
| 210.28 | 206.94 | 266.75 | 2.00 | 4.00 |
| 218.65 | 216.91 | 237.38 | 2.00 | 4.00 |
| 223.55 | 200.90 | 266.85 | 2.00 | 4.00 |
| 229.61 | 220.15 | 222.66 | 2.00 | 4.00 |
| 208.78 | 209.96 | 279.75 | 2.00 | 4.00 |
| 249.85 | 189.11 | 250.07 | 2.00 | 4.00 |
| 204.76 | 212.79 | 229.62 | 2.00 | 4.00 |
| 223.96 | 219.20 | 264.31 | 2.00 | 4.00 |
| 232.39 | 195.99 | 241.88 | 2.00 | 4.00 |
| 211.59 | 221.59 | 229.25 | 2.00 | 4.00 |

| | | | | |
|---|---|---|---|---|
| 194.25 | 216.90 | 274.96 | 2.00 | 4.00 |
| 231.82 | 197.04 | 243.73 | 2.00 | 4.00 |
| 212.07 | 232.22 | 243.36 | 2.00 | 4.00 |
| 199.62 | 198.11 | 262.52 | 2.00 | 4.00 |
| 205.06 | 199.67 | 241.17 | 2.00 | 4.00 |
| 220.25 | 266.48 | 234.67 | 2.00 | 4.00 |
| 221.30 | 242.74 | 233.66 | 2.00 | 4.00 |
| 217.93 | 211.06 | 284.70 | 2.00 | 4.00 |
| 196.56 | 207.88 | 213.27 | 2.00 | 4.00 |
| 227.73 | 234.96 | 266.27 | 2.00 | 4.00 |
| 204.01 | 243.16 | 212.60 | 2.00 | 4.00 |
| 234.26 | 233.03 | 261.49 | 2.00 | 4.00 |
| 206.29 | 224.55 | 217.24 | 2.00 | 4.00 |
| 221.76 | 192.14 | 255.79 | 2.00 | 4.00 |
| 226.76 | 266.15 | 254.10 | 2.00 | 4.00 |
| 225.52 | 237.16 | 230.69 | 2.00 | 4.00 |
| 221.71 | 214.81 | 239.85 | 2.00 | 4.00 |
| 191.98 | 209.79 | 223.18 | 2.00 | 4.00 |
| 215.49 | 225.03 | 276.03 | 2.00 | 4.00 |
| 213.62 | 262.80 | 231.83 | 2.00 | 4.00 |
| 220.94 | 235.21 | 237.39 | 2.00 | 4.00 |
| 214.16 | 208.44 | 226.56 | 2.00 | 4.00 |
| 197.26 | 208.92 | 227.78 | 2.00 | 4.00 |
| 205.54 | 213.19 | 279.32 | 2.00 | 4.00 |
| 219.90 | 238.55 | 232.73 | 2.00 | 4.00 |
| 210.31 | 258.30 | 234.33 | 2.00 | 4.00 |
| 222.47 | 213.83 | 255.48 | 2.00 | 4.00 |
| 224.21 | 209.96 | 225.77 | 2.00 | 4.00 |
| 182.01 | 210.92 | 258.77 | 2.00 | 4.00 |
| 210.43 | 207.90 | 226.05 | 2.00 | 4.00 |
| 192.65 | 233.00 | 243.86 | 2.00 | 4.00 |
| 229.88 | 245.93 | 273.62 | 2.00 | 4.00 |
| 231.39 | 220.38 | 263.27 | 2.00 | 4.00 |
| 224.84 | 212.96 | 234.63 | 2.00 | 4.00 |
| 195.57 | 221.53 | 236.47 | 2.00 | 4.00 |
| 220.86 | 215.60 | 262.92 | 2.00 | 4.00 |
| 189.83 | 218.70 | 277.43 | 2.00 | 4.00 |
| 206.56 | 245.46 | 247.04 | 2.00 | 4.00 |
| 242.46 | 220.50 | 229.96 | 2.00 | 4.00 |
| 227.60 | 213.87 | 275.43 | 2.00 | 4.00 |
| 221.31 | 228.43 | 255.75 | 2.00 | 4.00 |
| 224.13 | 240.86 | 242.44 | 2.00 | 4.00 |
| 206.24 | 231.18 | 228.67 | 2.00 | 4.00 |

| | | | | |
|---|---|---|---|---|
| 204.61 | 227.66 | 264.06 | 2.00 | 4.00 |
| 241.66 | 239.67 | 261.49 | 2.00 | 4.00 |
| 247.78 | 224.87 | 265.51 | 2.00 | 4.00 |
| 239.45 | 244.93 | 261.89 | 2.00 | 4.00 |
| 191.96 | 232.28 | 259.07 | 2.00 | 4.00 |
| 233.74 | 243.22 | 269.52 | 2.00 | 4.00 |
| 211.40 | 217.18 | 260.09 | 2.00 | 4.00 |
| 201.99 | 230.81 | 253.96 | 2.00 | 4.00 |
| 239.68 | 253.98 | 255.88 | 2.00 | 4.00 |
| 256.85 | 226.63 | 299.12 | 2.00 | 4.00 |
| 243.00 | 252.97 | 242.94 | 2.00 | 4.00 |
| 210.31 | 234.23 | 252.18 | 2.00 | 4.00 |
| 248.02 | 258.95 | 263.00 | 2.00 | 4.00 |
| 210.16 | 237.06 | 265.57 | 2.00 | 4.00 |
| 210.56 | 258.17 | 253.19 | 2.00 | 4.00 |
| 248.49 | 252.69 | 258.56 | 2.00 | 4.00 |
| 240.56 | 228.74 | 261.27 | 2.00 | 4.00 |
| 214.25 | 254.25 | 250.60 | 2.00 | 4.00 |
| 232.33 | 236.45 | 258.22 | 2.00 | 4.00 |
| 237.96 | 232.50 | 254.04 | 2.00 | 4.00 |
| 232.33 | 219.36 | 362.04 | 2.00 | 4.00 |
| 239.15 | 233.91 | 622.79 | 2.00 | 4.00 |
| 236.85 | 245.26 | 514.45 | 2.00 | 4.00 |
| 206.49 | 261.55 | 348.11 | 2.00 | 4.00 |
| 213.31 | 242.71 | 364.31 | 2.00 | 4.00 |
| 212.16 | 275.29 | 314.36 | 2.00 | 4.00 |
| 206.06 | 256.36 | 298.15 | 2.00 | 4.00 |
| 218.54 | 260.36 | 447.87 | 2.00 | 4.00 |
| 223.70 | 240.57 | 364.30 | 2.00 | 4.00 |
| 243.73 | 247.17 | 274.12 | 2.00 | 4.00 |
| 203.54 | 257.03 | 420.62 | 2.00 | 4.00 |
| 204.76 | 223.40 | 265.03 | 2.00 | 4.00 |
| 166.28 | 243.82 | 370.83 | 2.00 | 4.00 |
| 194.85 | 240.58 | 274.43 | 2.00 | 4.00 |
| 213.79 | 247.07 | 340.55 | 2.00 | 4.00 |
| 189.54 | 221.67 | 273.02 | 2.00 | 4.00 |
| 190.35 | 260.66 | 299.69 | 2.00 | 4.00 |
| 227.00 | 252.12 | 274.02 | 2.00 | 4.00 |
| 237.08 | 237.60 | 291.33 | 2.00 | 4.00 |
| 219.39 | 245.19 | 330.94 | 2.00 | 4.00 |
| 198.85 | 254.93 | 334.08 | 2.00 | 4.00 |
| 227.53 | 235.33 | 314.45 | 2.00 | 4.00 |
| 252.21 | 229.87 | 269.44 | 2.00 | 4.00 |

| | | | | |
|---|---|---|---|---|
| 217.06 | 238.64 | 285.53 | 2.00 | 4.00 |
| 193.23 | 279.77 | 352.30 | 2.00 | 4.00 |
| 231.75 | 290.34 | 299.04 | 2.00 | 4.00 |
| 254.61 | 229.13 | 350.77 | 2.00 | 4.00 |
| 187.95 | 238.57 | 273.46 | 2.00 | 4.00 |
| 216.45 | 227.71 | 318.63 | 2.00 | 4.00 |
| 206.92 | 268.37 | 385.87 | 2.00 | 4.00 |
| 248.77 | 256.08 | 281.22 | 2.00 | 4.00 |
| 212.70 | 246.17 | 314.86 | 2.00 | 4.00 |
| 208.56 | 264.84 | 330.39 | 2.00 | 4.00 |
| 178.50 | 236.05 | 320.16 | 2.00 | 4.00 |
| 216.81 | 225.98 | 358.44 | 2.00 | 4.00 |
| 253.60 | 271.89 | 316.11 | 2.00 | 4.00 |
| 214.90 | 256.85 | 325.14 | 2.00 | 4.00 |
| 225.09 | 244.15 | 322.86 | 2.00 | 4.00 |
| 183.96 | 243.25 | 299.57 | 2.00 | 4.00 |
| 211.99 | 257.65 | 328.56 | 2.00 | 4.00 |
| 239.63 | 234.03 | 356.87 | 2.00 | 4.00 |
| 195.07 | 274.04 | 289.62 | 2.00 | 4.00 |
| 224.08 | 254.24 | 376.11 | 2.00 | 4.00 |
| 233.48 | 222.57 | 372.18 | 2.00 | 4.00 |
| 196.87 | 241.55 | 369.77 | 2.00 | 4.00 |
| 216.79 | 268.38 | 304.06 | 2.00 | 4.00 |
| 197.93 | 232.08 | 278.43 | 2.00 | 4.00 |
| 205.37 | 269.02 | 329.13 | 2.00 | 4.00 |
| 219.23 | 265.89 | 410.83 | 2.00 | 4.00 |
| 194.90 | 213.75 | 330.19 | 2.00 | 4.00 |
| 193.67 | 246.47 | 387.90 | 2.00 | 4.00 |
| 206.11 | 270.08 | 340.09 | 2.00 | 4.00 |
| 223.10 | 235.34 | 285.08 | 2.00 | 4.00 |
| 239.85 | 256.02 | 286.05 | 2.00 | 4.00 |
| 201.17 | 284.16 | 403.81 | 2.00 | 4.00 |
| 170.04 | 222.22 | 322.57 | 2.00 | 4.00 |
| 207.78 | 254.68 | 340.04 | 2.00 | 4.00 |
| 218.82 | 248.55 | 334.86 | 2.00 | 4.00 |
| 254.65 | 248.32 | 308.81 | 2.00 | 4.00 |
| 221.75 | 245.99 | 272.95 | 2.00 | 4.00 |
| 202.92 | 283.03 | 376.87 | 2.00 | 4.00 |
| 201.46 | 236.19 | 332.18 | 2.00 | 4.00 |
| 223.67 | 249.64 | 320.19 | 2.00 | 4.00 |
| 209.70 | 244.70 | 373.64 | 2.00 | 4.00 |
| 206.55 | 253.54 | 283.49 | 2.00 | 4.00 |
| 196.49 | 250.78 | 307.31 | 2.00 | 4.00 |

| | | | | |
|---|---|---|---|---|
| 192.77 | 261.67 | 310.75 | 2.00 | 4.00 |
| 216.88 | 242.14 | 321.64 | 2.00 | 4.00 |
| 214.85 | 226.97 | 307.54 | 2.00 | 4.00 |
| 190.32 | 232.19 | 437.07 | 2.00 | 4.00 |
| 194.44 | 250.39 | 254.63 | 2.00 | 4.00 |
| 210.09 | 265.83 | 296.45 | 2.00 | 4.00 |
| 223.74 | 246.00 | 302.17 | 2.00 | 4.00 |
| 185.27 | 236.19 | 311.18 | 2.00 | 4.00 |
| 216.85 | 251.82 | 386.78 | 2.00 | 4.00 |
| 195.34 | 245.56 | 274.40 | 2.00 | 4.00 |
| 232.93 | 268.50 | 258.72 | 2.00 | 4.00 |
| 228.13 | 256.95 | 334.36 | 2.00 | 4.00 |
| 190.01 | 228.05 | 265.66 | 2.00 | 4.00 |
| 223.76 | 251.04 | 342.90 | 2.00 | 4.00 |
| 202.93 | 258.86 | 245.49 | 2.00 | 4.00 |
| 200.01 | 250.77 | 276.81 | 2.00 | 4.00 |
| 218.51 | 227.70 | 332.21 | 2.00 | 4.00 |
| 180.89 | 247.26 | 306.34 | 2.00 | 4.00 |
| 212.35 | 265.74 | 336.47 | 2.00 | 4.00 |
| 167.00 | 224.18 | 255.89 | 2.00 | 4.00 |
| 193.62 | 240.41 | 307.13 | 2.00 | 4.00 |
| 137.22 | 165.54 | 259.66 | 3.00 | 1.00 |
| 130.96 | 141.46 | 234.33 | 3.00 | 1.00 |
| 163.67 | 160.46 | 289.54 | 3.00 | 1.00 |
| 185.25 | 218.12 | 395.49 | 3.00 | 1.00 |
| 146.50 | 159.67 | 259.88 | 3.00 | 1.00 |
| 179.66 | 205.95 | 356.94 | 3.00 | 1.00 |
| 175.95 | 172.69 | 314.37 | 3.00 | 1.00 |
| 163.26 | 167.85 | 296.31 | 3.00 | 1.00 |
| 156.64 | 161.02 | 298.25 | 3.00 | 1.00 |
| 252.51 | 221.69 | 431.27 | 3.00 | 1.00 |
| 153.21 | 172.05 | 295.99 | 3.00 | 1.00 |
| 182.78 | 188.13 | 337.52 | 3.00 | 1.00 |
| 216.62 | 201.53 | 370.55 | 3.00 | 1.00 |
| 165.87 | 183.40 | 320.20 | 3.00 | 1.00 |
| 168.81 | 214.26 | 348.61 | 3.00 | 1.00 |
| 170.64 | 178.64 | 303.87 | 3.00 | 1.00 |
| 264.32 | 246.80 | 459.22 | 3.00 | 1.00 |
| 160.37 | 169.32 | 297.75 | 3.00 | 1.00 |
| 154.81 | 197.59 | 316.55 | 3.00 | 1.00 |
| 223.37 | 236.24 | 410.09 | 3.00 | 1.00 |
| 245.43 | 230.32 | 424.03 | 3.00 | 1.00 |
| 169.10 | 178.30 | 314.01 | 3.00 | 1.00 |

| | | | | |
|---|---|---|---|---|
| 176.29 | 186.98 | 318.10 | 3.00 | 1.00 |
| 206.68 | 210.87 | 394.80 | 3.00 | 1.00 |
| 216.16 | 221.87 | 400.44 | 3.00 | 1.00 |
| 178.73 | 185.80 | 317.09 | 3.00 | 1.00 |
| 167.29 | 166.06 | 317.48 | 3.00 | 1.00 |
| 243.93 | 285.49 | 498.40 | 3.00 | 1.00 |
| 258.33 | 276.89 | 523.28 | 3.00 | 1.00 |
| 200.76 | 206.73 | 365.90 | 3.00 | 1.00 |
| 188.12 | 172.62 | 325.09 | 3.00 | 1.00 |
| 200.16 | 189.95 | 367.79 | 3.00 | 1.00 |
| 301.83 | 357.39 | 603.59 | 3.00 | 1.00 |
| 245.88 | 293.00 | 530.76 | 3.00 | 1.00 |
| 198.44 | 204.45 | 353.55 | 3.00 | 1.00 |
| 217.35 | 216.83 | 378.97 | 3.00 | 1.00 |
| 216.82 | 247.65 | 404.33 | 3.00 | 1.00 |
| 342.21 | 335.55 | 599.75 | 3.00 | 1.00 |
| 295.68 | 312.18 | 546.41 | 3.00 | 1.00 |
| 192.70 | 224.08 | 409.49 | 3.00 | 1.00 |
| 214.71 | 227.28 | 373.50 | 3.00 | 1.00 |
| 240.15 | 250.45 | 414.78 | 3.00 | 1.00 |
| 256.63 | 296.02 | 481.29 | 3.00 | 1.00 |
| 325.61 | 343.11 | 613.32 | 3.00 | 1.00 |
| 214.54 | 208.80 | 387.35 | 3.00 | 1.00 |
| 225.91 | 242.11 | 452.53 | 3.00 | 1.00 |
| 227.88 | 265.46 | 390.75 | 3.00 | 1.00 |
| 273.51 | 277.60 | 457.29 | 3.00 | 1.00 |
| 282.26 | 304.76 | 515.74 | 3.00 | 1.00 |
| 329.62 | 328.49 | 593.25 | 3.00 | 1.00 |
| 218.98 | 209.88 | 390.68 | 3.00 | 1.00 |
| 230.05 | 264.00 | 454.53 | 3.00 | 1.00 |
| 199.40 | 241.58 | 349.58 | 3.00 | 1.00 |
| 265.52 | 310.08 | 479.78 | 3.00 | 1.00 |
| 267.08 | 283.25 | 454.10 | 3.00 | 1.00 |
| 309.37 | 316.62 | 527.40 | 3.00 | 1.00 |
| 206.17 | 188.79 | 360.76 | 3.00 | 1.00 |
| 284.21 | 305.66 | 528.41 | 3.00 | 1.00 |
| 174.68 | 186.74 | 319.58 | 3.00 | 1.00 |
| 295.84 | 340.66 | 523.44 | 3.00 | 1.00 |
| 223.57 | 260.92 | 413.21 | 3.00 | 1.00 |
| 296.66 | 334.56 | 534.40 | 3.00 | 1.00 |
| 271.00 | 323.28 | 511.08 | 3.00 | 1.00 |
| 166.17 | 145.39 | 268.95 | 3.00 | 1.00 |
| 234.21 | 275.11 | 461.59 | 3.00 | 1.00 |

| | | | | |
|---|---|---|---|---|
| 245.38 | 257.73 | 421.83 | 3.00 | 1.00 |
| 284.96 | 301.24 | 490.55 | 3.00 | 1.00 |
| 205.62 | 244.16 | 379.40 | 3.00 | 1.00 |
| 246.66 | 273.03 | 414.71 | 3.00 | 1.00 |
| 187.13 | 170.47 | 312.32 | 3.00 | 1.00 |
| 231.33 | 251.47 | 435.46 | 3.00 | 1.00 |
| 224.62 | 216.60 | 394.48 | 3.00 | 1.00 |
| 269.13 | 267.34 | 471.67 | 3.00 | 1.00 |
| 181.02 | 215.40 | 349.51 | 3.00 | 1.00 |
| 234.25 | 247.01 | 390.83 | 3.00 | 1.00 |
| 202.87 | 213.29 | 358.00 | 3.00 | 1.00 |
| 144.20 | 156.49 | 273.09 | 3.00 | 1.00 |
| 206.18 | 229.23 | 400.16 | 3.00 | 1.00 |
| 240.71 | 231.90 | 454.74 | 3.00 | 1.00 |
| 236.65 | 277.70 | 452.49 | 3.00 | 1.00 |
| 190.89 | 219.07 | 331.38 | 3.00 | 1.00 |
| 190.37 | 198.38 | 344.54 | 3.00 | 1.00 |
| 140.89 | 166.11 | 269.91 | 3.00 | 1.00 |
| 209.88 | 202.68 | 387.10 | 3.00 | 1.00 |
| 225.14 | 262.88 | 428.89 | 3.00 | 1.00 |
| 284.40 | 340.41 | 535.79 | 3.00 | 1.00 |
| 205.36 | 216.02 | 358.70 | 3.00 | 1.00 |
| 185.81 | 221.59 | 364.32 | 3.00 | 1.00 |
| 177.49 | 186.62 | 346.31 | 3.00 | 1.00 |
| 229.26 | 254.84 | 432.19 | 3.00 | 1.00 |
| 296.38 | 323.22 | 546.16 | 3.00 | 1.00 |
| 269.55 | 281.23 | 465.10 | 3.00 | 1.00 |
| 182.71 | 200.75 | 358.79 | 3.00 | 1.00 |
| 197.04 | 217.34 | 378.09 | 3.00 | 1.00 |
| 246.81 | 271.59 | 482.40 | 3.00 | 1.00 |
| 337.54 | 420.97 | 708.62 | 3.00 | 1.00 |
| 196.02 | 218.94 | 367.60 | 3.00 | 1.00 |
| 278.84 | 309.25 | 541.01 | 3.00 | 1.00 |
| 189.50 | 208.61 | 370.11 | 3.00 | 1.00 |
| 303.41 | 326.62 | 578.02 | 3.00 | 1.00 |
| 205.73 | 228.32 | 409.00 | 3.00 | 1.00 |
| 261.60 | 297.81 | 518.99 | 3.00 | 1.00 |
| 225.40 | 246.36 | 434.80 | 3.00 | 1.00 |
| 190.53 | 216.60 | 380.88 | 3.00 | 1.00 |
| 217.98 | 239.35 | 428.85 | 3.00 | 1.00 |
| 253.61 | 288.90 | 498.64 | 3.00 | 1.00 |
| 181.26 | 196.35 | 353.21 | 3.00 | 1.00 |
| 159.01 | 173.30 | 322.85 | 3.00 | 1.00 |

| | | | | |
|---|---|---|---|---|
| 459.75 | 471.85 | 882.63 | 3.00 | 1.00 |
| 168.20 | 191.44 | 332.61 | 3.00 | 1.00 |
| 184.93 | 205.20 | 345.16 | 3.00 | 1.00 |
| 261.29 | 343.82 | 686.77 | 3.00 | 1.00 |
| 262.36 | 312.81 | 596.80 | 3.00 | 1.00 |
| 266.16 | 266.33 | 554.14 | 3.00 | 1.00 |
| 233.81 | 282.33 | 523.82 | 3.00 | 1.00 |
| 219.60 | 195.39 | 444.61 | 3.00 | 1.00 |
| 216.80 | 287.76 | 509.49 | 3.00 | 1.00 |
| 228.20 | 202.44 | 439.53 | 3.00 | 1.00 |
| 188.53 | 242.85 | 486.89 | 3.00 | 1.00 |
| 311.28 | 319.13 | 572.69 | 3.00 | 1.00 |
| 224.24 | 223.88 | 449.94 | 3.00 | 1.00 |
| 208.73 | 284.89 | 587.00 | 3.00 | 1.00 |
| 310.62 | 344.12 | 647.50 | 3.00 | 1.00 |
| 360.54 | 405.13 | 778.49 | 3.00 | 1.00 |
| 200.20 | 301.04 | 566.26 | 3.00 | 1.00 |
| 344.11 | 378.76 | 738.40 | 3.00 | 1.00 |
| 355.20 | 340.90 | 695.25 | 3.00 | 1.00 |
| 282.57 | 445.82 | 813.68 | 3.00 | 1.00 |
| 241.07 | 299.11 | 547.64 | 3.00 | 1.00 |
| 404.53 | 491.01 | 877.16 | 3.00 | 1.00 |
| 228.32 | 242.74 | 532.21 | 3.00 | 1.00 |
| 203.44 | 298.62 | 552.64 | 3.00 | 1.00 |
| 238.35 | 292.97 | 523.97 | 3.00 | 1.00 |
| 335.55 | 461.65 | 813.93 | 3.00 | 1.00 |
| 237.98 | 270.99 | 513.94 | 3.00 | 1.00 |
| 233.95 | 305.40 | 670.81 | 3.00 | 1.00 |
| 213.91 | 267.87 | 523.55 | 3.00 | 1.00 |
| 379.05 | 508.54 | 898.38 | 3.00 | 1.00 |
| 283.67 | 371.37 | 727.06 | 3.00 | 1.00 |
| 323.51 | 406.83 | 752.99 | 3.00 | 1.00 |
| 253.58 | 340.97 | 679.54 | 3.00 | 1.00 |
| 209.71 | 258.04 | 472.78 | 3.00 | 1.00 |
| 270.99 | 329.20 | 633.85 | 3.00 | 1.00 |
| 262.29 | 313.11 | 532.96 | 3.00 | 1.00 |
| 314.27 | 398.67 | 787.93 | 3.00 | 1.00 |
| 148.12 | 197.55 | 354.64 | 3.00 | 1.00 |
| 190.75 | 237.18 | 472.20 | 3.00 | 1.00 |
| 224.10 | 318.25 | 558.18 | 3.00 | 1.00 |
| 261.68 | 354.28 | 617.96 | 3.00 | 1.00 |
| 277.89 | 353.32 | 662.56 | 3.00 | 1.00 |
| 177.59 | 230.02 | 401.56 | 3.00 | 1.00 |

| | | | | |
|---|---|---|---|---|
| 192.08 | 221.00 | 416.73 | 3.00 | 1.00 |
| 344.11 | 389.31 | 733.46 | 3.00 | 1.00 |
| 197.15 | 242.52 | 489.24 | 3.00 | 1.00 |
| 231.20 | 283.61 | 521.62 | 3.00 | 1.00 |
| 253.17 | 327.77 | 566.94 | 3.00 | 1.00 |
| 201.53 | 251.63 | 467.04 | 3.00 | 1.00 |
| 144.82 | 197.80 | 363.89 | 3.00 | 1.00 |
| 294.50 | 295.52 | 587.90 | 3.00 | 1.00 |
| 225.07 | 264.37 | 511.56 | 3.00 | 1.00 |
| 236.63 | 301.26 | 534.58 | 3.00 | 1.00 |
| 482.52 | 533.44 | 1059.75 | 3.00 | 1.00 |
| 324.91 | 471.33 | 768.13 | 3.00 | 1.00 |
| 157.81 | 228.63 | 400.80 | 3.00 | 1.00 |
| 325.42 | 371.00 | 688.21 | 3.00 | 1.00 |
| 244.91 | 271.84 | 536.33 | 3.00 | 1.00 |
| 286.72 | 353.71 | 665.87 | 3.00 | 1.00 |
| 407.02 | 551.16 | 975.46 | 3.00 | 1.00 |
| 514.86 | 584.08 | 1103.89 | 3.00 | 1.00 |
| 198.33 | 273.72 | 451.36 | 3.00 | 1.00 |
| 217.56 | 296.32 | 476.04 | 3.00 | 1.00 |
| 436.81 | 509.44 | 1013.84 | 3.00 | 1.00 |
| 200.86 | 234.53 | 401.88 | 3.00 | 2.00 |
| 279.57 | 284.25 | 540.24 | 3.00 | 2.00 |
| 192.82 | 190.88 | 383.16 | 3.00 | 2.00 |
| 271.98 | 272.05 | 511.49 | 3.00 | 2.00 |
| 228.93 | 225.25 | 444.92 | 3.00 | 2.00 |
| 172.44 | 186.00 | 320.77 | 3.00 | 2.00 |
| 202.78 | 207.51 | 399.59 | 3.00 | 2.00 |
| 187.22 | 205.14 | 352.42 | 3.00 | 2.00 |
| 165.57 | 190.12 | 332.44 | 3.00 | 2.00 |
| 211.26 | 241.62 | 448.39 | 3.00 | 2.00 |
| 200.94 | 201.81 | 378.18 | 3.00 | 2.00 |
| 202.42 | 207.04 | 396.67 | 3.00 | 2.00 |
| 231.62 | 228.57 | 478.79 | 3.00 | 2.00 |
| 173.19 | 202.33 | 390.68 | 3.00 | 2.00 |
| 202.79 | 192.62 | 375.29 | 3.00 | 2.00 |
| 264.01 | 245.74 | 499.87 | 3.00 | 2.00 |
| 198.25 | 182.85 | 403.22 | 3.00 | 2.00 |
| 174.31 | 193.79 | 369.08 | 3.00 | 2.00 |
| 261.29 | 239.96 | 476.67 | 3.00 | 2.00 |
| 208.59 | 162.24 | 383.21 | 3.00 | 2.00 |
| 250.55 | 221.09 | 484.90 | 3.00 | 2.00 |
| 202.93 | 222.69 | 412.36 | 3.00 | 2.00 |

| | | | | |
|---|---|---|---|---|
| 236.43 | 246.15 | 481.70 | 3.00 | 2.00 |
| 202.88 | 183.80 | 375.91 | 3.00 | 2.00 |
| 280.70 | 231.27 | 512.32 | 3.00 | 2.00 |
| 227.37 | 255.76 | 463.52 | 3.00 | 2.00 |
| 189.83 | 207.43 | 403.25 | 3.00 | 2.00 |
| 210.95 | 209.08 | 421.99 | 3.00 | 2.00 |
| 322.76 | 302.10 | 639.38 | 3.00 | 2.00 |
| 236.69 | 240.51 | 471.34 | 3.00 | 2.00 |
| 292.09 | 268.11 | 501.17 | 3.00 | 2.00 |
| 286.89 | 303.03 | 605.87 | 3.00 | 2.00 |
| 190.41 | 203.56 | 384.75 | 3.00 | 2.00 |
| 273.91 | 275.59 | 566.93 | 3.00 | 2.00 |
| 210.03 | 209.96 | 415.54 | 3.00 | 2.00 |
| 255.59 | 262.46 | 522.64 | 3.00 | 2.00 |
| 339.80 | 285.26 | 592.38 | 3.00 | 2.00 |
| 220.67 | 234.91 | 477.25 | 3.00 | 2.00 |
| 181.79 | 184.20 | 387.43 | 3.00 | 2.00 |
| 174.09 | 163.67 | 359.53 | 3.00 | 2.00 |
| 220.26 | 257.49 | 501.17 | 3.00 | 2.00 |
| 234.36 | 254.48 | 491.12 | 3.00 | 2.00 |
| 229.15 | 204.62 | 397.16 | 3.00 | 2.00 |
| 169.81 | 151.25 | 326.74 | 3.00 | 2.00 |
| 178.21 | 172.85 | 359.08 | 3.00 | 2.00 |
| 189.63 | 165.16 | 361.14 | 3.00 | 2.00 |
| 246.07 | 255.31 | 512.43 | 3.00 | 2.00 |
| 219.48 | 232.25 | 453.86 | 3.00 | 2.00 |
| 295.55 | 275.34 | 602.56 | 3.00 | 2.00 |
| 202.85 | 157.57 | 353.50 | 3.00 | 2.00 |
| 195.72 | 178.56 | 351.76 | 3.00 | 2.00 |
| 163.92 | 160.57 | 330.01 | 3.00 | 2.00 |
| 257.28 | 230.84 | 480.70 | 3.00 | 2.00 |
| 217.78 | 235.09 | 466.17 | 3.00 | 2.00 |
| 327.05 | 312.88 | 670.32 | 3.00 | 2.00 |
| 246.03 | 253.58 | 525.87 | 3.00 | 2.00 |
| 217.06 | 180.81 | 368.03 | 3.00 | 2.00 |
| 153.19 | 136.79 | 289.15 | 3.00 | 2.00 |
| 217.29 | 173.58 | 389.16 | 3.00 | 2.00 |
| 283.61 | 243.68 | 488.83 | 3.00 | 2.00 |
| 210.87 | 216.70 | 429.78 | 3.00 | 2.00 |
| 401.53 | 431.09 | 854.51 | 3.00 | 2.00 |
| 309.44 | 299.21 | 601.25 | 3.00 | 2.00 |
| 217.21 | 201.27 | 439.04 | 3.00 | 2.00 |
| 155.92 | 162.91 | 322.50 | 3.00 | 2.00 |

| | | | | |
|---|---|---|---|---|
| 296.56 | 219.13 | 502.58 | 3.00 | 2.00 |
| 169.89 | 192.16 | 370.90 | 3.00 | 2.00 |
| 245.67 | 256.79 | 499.26 | 3.00 | 2.00 |
| 313.46 | 331.27 | 649.52 | 3.00 | 2.00 |
| 243.45 | 255.02 | 534.74 | 3.00 | 2.00 |
| 172.86 | 173.65 | 371.69 | 3.00 | 2.00 |
| 165.14 | 148.81 | 282.09 | 3.00 | 2.00 |
| 212.81 | 218.37 | 450.50 | 3.00 | 2.00 |
| 192.47 | 207.73 | 389.54 | 3.00 | 2.00 |
| 226.64 | 221.50 | 456.13 | 3.00 | 2.00 |
| 249.37 | 299.90 | 569.63 | 3.00 | 2.00 |
| 288.29 | 245.86 | 515.70 | 3.00 | 2.00 |
| 169.17 | 185.85 | 376.99 | 3.00 | 2.00 |
| 132.93 | 150.63 | 289.14 | 3.00 | 2.00 |
| 211.28 | 218.06 | 426.24 | 3.00 | 2.00 |
| 185.87 | 230.40 | 447.50 | 3.00 | 2.00 |
| 217.42 | 245.94 | 475.91 | 3.00 | 2.00 |
| 244.52 | 251.96 | 467.19 | 3.00 | 2.00 |
| 209.96 | 203.32 | 430.73 | 3.00 | 2.00 |
| 180.61 | 195.07 | 360.10 | 3.00 | 2.00 |
| 160.36 | 205.07 | 372.32 | 3.00 | 2.00 |
| 147.59 | 186.65 | 356.44 | 3.00 | 2.00 |
| 249.66 | 262.23 | 495.00 | 3.00 | 2.00 |
| 128.57 | 157.16 | 321.16 | 3.00 | 2.00 |
| 112.86 | 142.87 | 287.66 | 3.00 | 2.00 |
| 168.38 | 198.72 | 417.63 | 3.00 | 2.00 |
| 154.92 | 179.21 | 354.73 | 3.00 | 2.00 |
| 157.82 | 234.91 | 457.81 | 3.00 | 2.00 |
| 150.79 | 183.10 | 337.85 | 3.00 | 2.00 |
| 155.29 | 237.34 | 456.42 | 3.00 | 2.00 |
| 131.69 | 196.91 | 381.65 | 3.00 | 2.00 |
| 163.54 | 191.21 | 375.57 | 3.00 | 2.00 |
| 138.39 | 224.89 | 421.03 | 3.00 | 2.00 |
| 152.63 | 228.88 | 440.80 | 3.00 | 2.00 |
| 142.88 | 172.54 | 333.51 | 3.00 | 2.00 |
| 139.98 | 173.87 | 330.19 | 3.00 | 2.00 |
| 160.92 | 236.27 | 460.07 | 3.00 | 2.00 |
| 165.27 | 195.26 | 353.61 | 3.00 | 2.00 |
| 131.13 | 159.68 | 288.60 | 3.00 | 2.00 |
| 113.38 | 151.30 | 291.13 | 3.00 | 2.00 |
| 202.87 | 279.46 | 552.77 | 3.00 | 2.00 |
| 169.22 | 227.52 | 416.28 | 3.00 | 2.00 |
| 112.17 | 156.60 | 279.93 | 3.00 | 2.00 |

| | | | | |
|---|---|---|---|---|
| 138.08 | 167.67 | 325.97 | 3.00 | 2.00 |
| 239.22 | 300.32 | 604.46 | 3.00 | 2.00 |
| 201.36 | 261.44 | 508.47 | 3.00 | 2.00 |
| 131.76 | 179.91 | 318.09 | 3.00 | 2.00 |
| 151.13 | 183.04 | 374.12 | 3.00 | 2.00 |
| 138.45 | 191.03 | 341.36 | 3.00 | 2.00 |
| 238.88 | 277.00 | 538.73 | 3.00 | 2.00 |
| 134.28 | 164.17 | 300.26 | 3.00 | 2.00 |
| 144.50 | 165.77 | 330.63 | 3.00 | 2.00 |
| 169.62 | 205.09 | 408.01 | 3.00 | 2.00 |
| 153.22 | 204.90 | 368.70 | 3.00 | 2.00 |
| 177.66 | 214.09 | 405.57 | 3.00 | 2.00 |
| 126.25 | 178.03 | 335.87 | 3.00 | 2.00 |
| 155.28 | 172.46 | 369.94 | 3.00 | 2.00 |
| 278.52 | 299.54 | 606.17 | 3.00 | 2.00 |
| 142.49 | 212.93 | 350.07 | 3.00 | 2.00 |
| 266.74 | 343.52 | 637.71 | 3.00 | 2.00 |
| 152.05 | 210.32 | 406.60 | 3.00 | 2.00 |
| 153.78 | 182.51 | 354.67 | 3.00 | 2.00 |
| 220.97 | 240.41 | 460.14 | 3.00 | 2.00 |
| 190.09 | 185.69 | 403.06 | 3.00 | 2.00 |
| 141.33 | 164.56 | 310.33 | 3.00 | 2.00 |
| 373.92 | 437.91 | 890.33 | 3.00 | 2.00 |
| 147.50 | 243.14 | 438.58 | 3.00 | 2.00 |
| 151.42 | 197.72 | 389.37 | 3.00 | 2.00 |
| 192.58 | 209.11 | 440.35 | 3.00 | 2.00 |
| 252.10 | 278.92 | 499.81 | 3.00 | 2.00 |
| 188.23 | 203.12 | 388.35 | 3.00 | 2.00 |
| 209.73 | 239.41 | 517.80 | 3.00 | 2.00 |
| 255.39 | 361.84 | 655.29 | 3.00 | 2.00 |
| 176.12 | 211.93 | 439.87 | 3.00 | 2.00 |
| 157.56 | 219.72 | 436.07 | 3.00 | 2.00 |
| 178.04 | 210.44 | 399.90 | 3.00 | 2.00 |
| 200.75 | 190.95 | 393.63 | 3.00 | 2.00 |
| 354.32 | 429.78 | 729.67 | 3.00 | 2.00 |
| 240.64 | 308.85 | 610.82 | 3.00 | 2.00 |
| 163.12 | 200.16 | 437.74 | 3.00 | 2.00 |
| 228.10 | 236.49 | 453.25 | 3.00 | 2.00 |
| 195.90 | 248.54 | 490.59 | 3.00 | 2.00 |
| 173.90 | 234.99 | 397.55 | 3.00 | 2.00 |
| 178.69 | 208.37 | 403.42 | 3.00 | 2.00 |
| 231.80 | 222.06 | 506.16 | 3.00 | 2.00 |
| 268.17 | 314.07 | 620.94 | 3.00 | 2.00 |

| | | | | |
|---|---|---|---|---|
| 280.72 | 394.60 | 678.41 | 3.00 | 2.00 |
| 203.48 | 297.77 | 512.52 | 3.00 | 2.00 |
| 185.73 | 208.66 | 425.34 | 3.00 | 2.00 |
| 143.30 | 180.74 | 334.35 | 3.00 | 2.00 |
| 256.00 | 318.47 | 584.51 | 3.00 | 2.00 |
| 178.27 | 222.01 | 409.90 | 3.00 | 2.00 |
| 205.93 | 227.03 | 474.73 | 3.00 | 2.00 |
| 221.07 | 292.51 | 536.55 | 3.00 | 2.00 |
| 253.53 | 257.81 | 506.10 | 3.00 | 2.00 |
| 154.48 | 162.13 | 350.59 | 3.00 | 2.00 |
| 252.68 | 275.70 | 501.99 | 3.00 | 2.00 |
| 224.16 | 238.55 | 499.08 | 3.00 | 2.00 |
| 174.99 | 193.70 | 391.56 | 3.00 | 2.00 |
| 172.81 | 223.78 | 424.80 | 3.00 | 2.00 |
| 185.48 | 219.79 | 461.68 | 3.00 | 2.00 |
| 177.31 | 218.04 | 396.17 | 3.00 | 2.00 |
| 231.20 | 233.41 | 481.25 | 3.00 | 2.00 |
| 182.37 | 219.27 | 396.40 | 3.00 | 2.00 |
| 215.48 | 187.83 | 378.59 | 3.00 | 3.00 |
| 171.17 | 155.59 | 312.07 | 3.00 | 3.00 |
| 186.59 | 180.32 | 347.60 | 3.00 | 3.00 |
| 210.74 | 225.22 | 412.55 | 3.00 | 3.00 |
| 186.25 | 212.41 | 358.52 | 3.00 | 3.00 |
| 225.86 | 217.51 | 411.00 | 3.00 | 3.00 |
| 168.40 | 188.09 | 336.08 | 3.00 | 3.00 |
| 143.29 | 163.73 | 344.57 | 3.00 | 3.00 |
| 222.33 | 193.39 | 409.66 | 3.00 | 3.00 |
| 165.95 | 141.82 | 342.26 | 3.00 | 3.00 |
| 119.93 | 149.07 | 312.12 | 3.00 | 3.00 |
| 201.50 | 190.78 | 379.63 | 3.00 | 3.00 |
| 171.40 | 167.23 | 383.12 | 3.00 | 3.00 |
| 154.25 | 174.58 | 379.33 | 3.00 | 3.00 |
| 146.04 | 151.95 | 306.28 | 3.00 | 3.00 |
| 166.11 | 177.07 | 335.33 | 3.00 | 3.00 |
| 154.55 | 172.68 | 340.16 | 3.00 | 3.00 |
| 154.48 | 168.71 | 335.37 | 3.00 | 3.00 |
| 142.22 | 158.20 | 321.26 | 3.00 | 3.00 |
| 151.09 | 175.73 | 339.48 | 3.00 | 3.00 |
| 155.82 | 173.78 | 319.46 | 3.00 | 3.00 |
| 178.79 | 198.30 | 380.38 | 3.00 | 3.00 |
| 198.83 | 178.30 | 361.97 | 3.00 | 3.00 |
| 161.40 | 164.30 | 324.97 | 3.00 | 3.00 |
| 125.96 | 159.87 | 304.88 | 3.00 | 3.00 |

| | | | | |
|---|---|---|---|---|
| 159.65 | 177.84 | 343.94 | 3.00 | 3.00 |
| 157.74 | 162.22 | 311.20 | 3.00 | 3.00 |
| 210.88 | 193.71 | 386.09 | 3.00 | 3.00 |
| 151.17 | 161.30 | 309.55 | 3.00 | 3.00 |
| 163.08 | 180.32 | 370.89 | 3.00 | 3.00 |
| 155.25 | 179.07 | 336.80 | 3.00 | 3.00 |
| 147.44 | 148.60 | 289.28 | 3.00 | 3.00 |
| 201.21 | 210.67 | 412.54 | 3.00 | 3.00 |
| 282.90 | 259.49 | 497.30 | 3.00 | 3.00 |
| 204.80 | 214.35 | 423.71 | 3.00 | 3.00 |
| 187.26 | 216.65 | 405.02 | 3.00 | 3.00 |
| 166.31 | 163.72 | 335.03 | 3.00 | 3.00 |
| 171.97 | 165.84 | 338.59 | 3.00 | 3.00 |
| 198.52 | 188.58 | 401.64 | 3.00 | 3.00 |
| 334.23 | 334.39 | 622.69 | 3.00 | 3.00 |
| 136.91 | 166.46 | 332.17 | 3.00 | 3.00 |
| 241.72 | 256.92 | 500.27 | 3.00 | 3.00 |
| 176.76 | 183.58 | 368.88 | 3.00 | 3.00 |
| 189.68 | 202.51 | 374.89 | 3.00 | 3.00 |
| 193.85 | 179.41 | 392.09 | 3.00 | 3.00 |
| 199.08 | 189.95 | 388.62 | 3.00 | 3.00 |
| 240.34 | 265.84 | 497.80 | 3.00 | 3.00 |
| 177.05 | 212.25 | 437.41 | 3.00 | 3.00 |
| 246.00 | 271.36 | 505.22 | 3.00 | 3.00 |
| 189.82 | 210.01 | 394.15 | 3.00 | 3.00 |
| 199.38 | 188.82 | 416.16 | 3.00 | 3.00 |
| 247.25 | 228.93 | 489.05 | 3.00 | 3.00 |
| 196.88 | 205.39 | 382.27 | 3.00 | 3.00 |
| 237.16 | 197.12 | 478.49 | 3.00 | 3.00 |
| 132.91 | 193.88 | 342.35 | 3.00 | 3.00 |
| 164.47 | 209.63 | 387.82 | 3.00 | 3.00 |
| 221.47 | 208.46 | 414.17 | 3.00 | 3.00 |
| 213.73 | 186.73 | 416.76 | 3.00 | 3.00 |
| 254.99 | 232.80 | 469.78 | 3.00 | 3.00 |
| 163.30 | 185.54 | 332.50 | 3.00 | 3.00 |
| 289.06 | 233.61 | 589.04 | 3.00 | 3.00 |
| 209.43 | 227.25 | 456.60 | 3.00 | 3.00 |
| 123.08 | 161.44 | 314.97 | 3.00 | 3.00 |
| 188.46 | 207.23 | 430.33 | 3.00 | 3.00 |
| 208.72 | 179.53 | 409.21 | 3.00 | 3.00 |
| 202.94 | 232.28 | 410.10 | 3.00 | 3.00 |
| 201.67 | 217.43 | 393.84 | 3.00 | 3.00 |
| 216.81 | 192.72 | 411.24 | 3.00 | 3.00 |

| | | | | |
|---|---|---|---|---|
| 275.62 | 279.58 | 580.34 | 3.00 | 3.00 |
| 137.56 | 159.93 | 330.65 | 3.00 | 3.00 |
| 166.54 | 211.66 | 420.52 | 3.00 | 3.00 |
| 171.60 | 173.05 | 404.01 | 3.00 | 3.00 |
| 207.84 | 214.03 | 420.17 | 3.00 | 3.00 |
| 167.93 | 175.20 | 345.55 | 3.00 | 3.00 |
| 209.17 | 184.04 | 430.17 | 3.00 | 3.00 |
| 212.63 | 212.67 | 434.52 | 3.00 | 3.00 |
| 147.92 | 174.86 | 333.41 | 3.00 | 3.00 |
| 168.93 | 216.14 | 408.47 | 3.00 | 3.00 |
| 171.98 | 168.81 | 389.24 | 3.00 | 3.00 |
| 246.52 | 252.97 | 492.11 | 3.00 | 3.00 |
| 155.76 | 152.65 | 327.88 | 3.00 | 3.00 |
| 211.43 | 210.72 | 429.15 | 3.00 | 3.00 |
| 209.68 | 204.62 | 415.38 | 3.00 | 3.00 |
| 168.38 | 210.08 | 379.65 | 3.00 | 3.00 |
| 157.43 | 180.56 | 384.06 | 3.00 | 3.00 |
| 213.12 | 231.28 | 433.43 | 3.00 | 3.00 |
| 256.03 | 257.29 | 525.16 | 3.00 | 3.00 |
| 152.12 | 169.58 | 308.65 | 3.00 | 3.00 |
| 201.28 | 176.59 | 370.63 | 3.00 | 3.00 |
| 181.38 | 197.81 | 379.84 | 3.00 | 3.00 |
| 154.45 | 208.42 | 358.55 | 3.00 | 3.00 |
| 209.73 | 242.91 | 463.52 | 3.00 | 3.00 |
| 230.59 | 287.50 | 511.37 | 3.00 | 3.00 |
| 148.07 | 151.35 | 324.40 | 3.00 | 3.00 |
| 184.91 | 197.58 | 362.67 | 3.00 | 3.00 |
| 177.62 | 193.62 | 335.50 | 3.00 | 3.00 |
| 176.67 | 170.76 | 379.27 | 3.00 | 3.00 |
| 219.57 | 213.63 | 421.08 | 3.00 | 3.00 |
| 190.22 | 224.54 | 433.93 | 3.00 | 3.00 |
| 160.28 | 184.60 | 335.61 | 3.00 | 3.00 |
| 156.38 | 156.92 | 307.32 | 3.00 | 3.00 |
| 184.57 | 176.67 | 369.65 | 3.00 | 3.00 |
| 163.16 | 176.29 | 334.77 | 3.00 | 3.00 |
| 221.26 | 211.10 | 399.19 | 3.00 | 3.00 |
| 143.94 | 164.78 | 323.02 | 3.00 | 3.00 |
| 178.18 | 159.21 | 331.50 | 3.00 | 3.00 |
| 191.75 | 188.85 | 378.87 | 3.00 | 3.00 |
| 169.01 | 180.28 | 355.67 | 3.00 | 3.00 |
| 211.83 | 176.56 | 382.10 | 3.00 | 3.00 |
| 164.05 | 241.84 | 407.70 | 3.00 | 3.00 |
| 161.56 | 226.37 | 409.67 | 3.00 | 3.00 |

| | | | | |
|---|---|---|---|---|
| 134.67 | 193.71 | 361.72 | 3.00 | 3.00 |
| 164.58 | 210.53 | 397.80 | 3.00 | 3.00 |
| 159.18 | 242.13 | 419.73 | 3.00 | 3.00 |
| 142.12 | 196.36 | 350.62 | 3.00 | 3.00 |
| 155.72 | 248.61 | 399.44 | 3.00 | 3.00 |
| 196.82 | 274.43 | 486.31 | 3.00 | 3.00 |
| 148.69 | 207.43 | 352.54 | 3.00 | 3.00 |
| 192.39 | 283.78 | 476.03 | 3.00 | 3.00 |
| 143.16 | 205.69 | 390.75 | 3.00 | 3.00 |
| 157.59 | 209.96 | 375.38 | 3.00 | 3.00 |
| 160.45 | 228.46 | 409.64 | 3.00 | 3.00 |
| 206.82 | 240.77 | 446.47 | 3.00 | 3.00 |
| 191.98 | 247.34 | 477.67 | 3.00 | 3.00 |
| 160.67 | 229.15 | 397.59 | 3.00 | 3.00 |
| 192.05 | 249.30 | 430.85 | 3.00 | 3.00 |
| 185.46 | 265.07 | 424.70 | 3.00 | 3.00 |
| 201.53 | 252.95 | 457.58 | 3.00 | 3.00 |
| 163.57 | 216.07 | 398.89 | 3.00 | 3.00 |
| 172.09 | 263.45 | 418.74 | 3.00 | 3.00 |
| 210.24 | 282.62 | 483.24 | 3.00 | 3.00 |
| 170.45 | 242.72 | 399.13 | 3.00 | 3.00 |
| 198.61 | 221.88 | 427.58 | 3.00 | 3.00 |
| 170.65 | 188.42 | 358.59 | 3.00 | 3.00 |
| 181.01 | 222.70 | 389.64 | 3.00 | 3.00 |
| 173.31 | 257.90 | 428.38 | 3.00 | 3.00 |
| 184.00 | 257.82 | 411.37 | 3.00 | 3.00 |
| 179.58 | 232.80 | 395.24 | 3.00 | 3.00 |
| 171.07 | 190.68 | 325.56 | 3.00 | 3.00 |
| 196.98 | 239.81 | 406.41 | 3.00 | 3.00 |
| 166.12 | 224.97 | 390.87 | 3.00 | 3.00 |
| 240.29 | 289.98 | 514.74 | 3.00 | 3.00 |
| 187.96 | 218.41 | 377.84 | 3.00 | 3.00 |
| 123.27 | 167.96 | 293.29 | 3.00 | 3.00 |
| 165.15 | 226.93 | 379.62 | 3.00 | 3.00 |
| 174.29 | 218.89 | 380.10 | 3.00 | 3.00 |
| 197.95 | 250.59 | 437.62 | 3.00 | 3.00 |
| 196.80 | 232.54 | 406.11 | 3.00 | 3.00 |
| 152.39 | 187.58 | 337.18 | 3.00 | 3.00 |
| 111.53 | 175.73 | 280.94 | 3.00 | 3.00 |
| 151.72 | 215.65 | 390.51 | 3.00 | 3.00 |
| 155.29 | 211.72 | 363.21 | 3.00 | 3.00 |
| 183.31 | 257.77 | 452.67 | 3.00 | 3.00 |
| 147.68 | 186.96 | 305.38 | 3.00 | 3.00 |

| | | | | |
|---|---|---|---|---|
| 150.46 | 180.16 | 326.39 | 3.00 | 3.00 |
| 132.33 | 206.61 | 344.32 | 3.00 | 3.00 |
| 119.91 | 174.38 | 321.61 | 3.00 | 3.00 |
| 149.75 | 211.98 | 375.88 | 3.00 | 3.00 |
| 153.92 | 197.57 | 349.47 | 3.00 | 3.00 |
| 141.48 | 185.08 | 299.96 | 3.00 | 3.00 |
| 136.90 | 165.30 | 298.75 | 3.00 | 3.00 |
| 137.10 | 244.28 | 386.39 | 3.00 | 3.00 |
| 133.20 | 184.36 | 326.66 | 3.00 | 3.00 |
| 141.02 | 184.49 | 327.04 | 3.00 | 3.00 |
| 123.95 | 165.99 | 294.45 | 3.00 | 3.00 |
| 172.79 | 217.64 | 366.83 | 3.00 | 3.00 |
| 180.46 | 215.43 | 400.53 | 3.00 | 3.00 |
| 112.22 | 185.17 | 311.91 | 3.00 | 3.00 |
| 143.18 | 202.32 | 357.54 | 3.00 | 3.00 |
| 132.80 | 186.84 | 310.27 | 3.00 | 3.00 |
| 125.57 | 170.65 | 305.45 | 3.00 | 3.00 |
| 168.55 | 217.53 | 384.39 | 3.00 | 3.00 |
| 119.65 | 193.80 | 321.24 | 3.00 | 3.00 |
| 145.82 | 203.26 | 371.34 | 3.00 | 3.00 |
| 173.47 | 223.07 | 380.41 | 3.00 | 3.00 |
| 156.98 | 193.84 | 356.66 | 3.00 | 3.00 |
| 135.32 | 162.63 | 282.23 | 3.00 | 3.00 |
| 167.38 | 215.68 | 373.76 | 3.00 | 3.00 |
| 172.41 | 207.03 | 376.92 | 3.00 | 3.00 |
| 188.83 | 222.74 | 382.30 | 3.00 | 3.00 |
| 124.53 | 165.26 | 296.15 | 3.00 | 3.00 |
| 155.21 | 199.62 | 362.57 | 3.00 | 3.00 |
| 235.99 | 261.93 | 468.38 | 3.00 | 3.00 |
| 196.99 | 221.90 | 391.98 | 3.00 | 3.00 |
| 191.26 | 256.36 | 449.59 | 3.00 | 3.00 |
| 205.41 | 288.13 | 524.57 | 3.00 | 3.00 |
| 180.63 | 224.37 | 393.07 | 3.00 | 3.00 |
| 227.76 | 282.08 | 477.46 | 3.00 | 3.00 |
| 160.17 | 213.52 | 378.11 | 3.00 | 3.00 |
| 145.90 | 196.26 | 363.00 | 3.00 | 3.00 |
| 182.73 | 233.77 | 427.11 | 3.00 | 3.00 |
| 165.93 | 224.36 | 396.91 | 3.00 | 3.00 |
| 170.61 | 253.95 | 441.89 | 3.00 | 3.00 |
| 187.21 | 249.56 | 447.52 | 3.00 | 3.00 |
| 175.83 | 229.99 | 427.18 | 3.00 | 3.00 |
| 216.48 | 209.52 | 246.95 | 3.00 | 4.00 |
| 213.54 | 193.70 | 263.21 | 3.00 | 4.00 |

| | | | | |
|---|---|---|---|---|
| 221.66 | 202.36 | 253.73 | 3.00 | 4.00 |
| 218.20 | 212.22 | 214.23 | 3.00 | 4.00 |
| 236.97 | 196.61 | 255.22 | 3.00 | 4.00 |
| 214.07 | 208.38 | 212.87 | 3.00 | 4.00 |
| 225.41 | 209.71 | 273.31 | 3.00 | 4.00 |
| 244.92 | 184.22 | 238.79 | 3.00 | 4.00 |
| 200.47 | 221.61 | 245.08 | 3.00 | 4.00 |
| 235.10 | 220.01 | 259.76 | 3.00 | 4.00 |
| 230.91 | 231.36 | 246.91 | 3.00 | 4.00 |
| 206.02 | 227.10 | 239.37 | 3.00 | 4.00 |
| 206.53 | 219.64 | 261.36 | 3.00 | 4.00 |
| 225.35 | 194.91 | 242.14 | 3.00 | 4.00 |
| 222.79 | 222.94 | 250.87 | 3.00 | 4.00 |
| 194.34 | 204.69 | 279.13 | 3.00 | 4.00 |
| 209.66 | 196.28 | 233.61 | 3.00 | 4.00 |
| 221.23 | 257.19 | 244.37 | 3.00 | 4.00 |
| 231.14 | 237.70 | 230.82 | 3.00 | 4.00 |
| 210.29 | 219.81 | 293.60 | 3.00 | 4.00 |
| 199.82 | 203.76 | 205.20 | 3.00 | 4.00 |
| 233.62 | 271.55 | 286.70 | 3.00 | 4.00 |
| 221.66 | 239.43 | 218.39 | 3.00 | 4.00 |
| 230.90 | 229.95 | 274.82 | 3.00 | 4.00 |
| 196.43 | 211.47 | 211.46 | 3.00 | 4.00 |
| 227.02 | 208.71 | 268.61 | 3.00 | 4.00 |
| 217.91 | 253.61 | 243.22 | 3.00 | 4.00 |
| 226.03 | 235.35 | 225.88 | 3.00 | 4.00 |
| 223.81 | 213.44 | 231.00 | 3.00 | 4.00 |
| 188.24 | 205.20 | 235.39 | 3.00 | 4.00 |
| 215.01 | 225.40 | 285.88 | 3.00 | 4.00 |
| 204.79 | 257.88 | 237.32 | 3.00 | 4.00 |
| 228.10 | 224.21 | 233.76 | 3.00 | 4.00 |
| 214.29 | 207.76 | 220.97 | 3.00 | 4.00 |
| 185.27 | 201.61 | 250.69 | 3.00 | 4.00 |
| 205.91 | 218.94 | 276.48 | 3.00 | 4.00 |
| 213.61 | 241.90 | 230.53 | 3.00 | 4.00 |
| 212.75 | 251.57 | 241.00 | 3.00 | 4.00 |
| 225.83 | 223.92 | 252.69 | 3.00 | 4.00 |
| 226.84 | 210.76 | 233.38 | 3.00 | 4.00 |
| 193.10 | 207.21 | 263.47 | 3.00 | 4.00 |
| 206.25 | 202.67 | 226.21 | 3.00 | 4.00 |
| 193.57 | 234.15 | 244.09 | 3.00 | 4.00 |
| 218.46 | 236.66 | 269.79 | 3.00 | 4.00 |
| 230.79 | 229.77 | 271.69 | 3.00 | 4.00 |

| | | | | |
|---|---|---|---|---|
| 226.34 | 215.05 | 234.47 | 3.00 | 4.00 |
| 211.18 | 225.77 | 238.06 | 3.00 | 4.00 |
| 210.48 | 209.66 | 274.63 | 3.00 | 4.00 |
| 191.94 | 224.68 | 283.95 | 3.00 | 4.00 |
| 258.79 | 236.64 | 230.70 | 3.00 | 4.00 |
| 250.83 | 215.12 | 233.39 | 3.00 | 4.00 |
| 230.82 | 224.71 | 265.45 | 3.00 | 4.00 |
| 213.39 | 223.32 | 274.11 | 3.00 | 4.00 |
| 214.36 | 240.36 | 238.78 | 3.00 | 4.00 |
| 209.87 | 257.64 | 236.91 | 3.00 | 4.00 |
| 207.94 | 229.30 | 268.88 | 3.00 | 4.00 |
| 232.39 | 244.64 | 268.79 | 3.00 | 4.00 |
| 246.50 | 222.43 | 268.74 | 3.00 | 4.00 |
| 243.04 | 250.21 | 281.35 | 3.00 | 4.00 |
| 190.17 | 231.69 | 245.49 | 3.00 | 4.00 |
| 243.65 | 233.21 | 257.54 | 3.00 | 4.00 |
| 202.42 | 251.12 | 274.94 | 3.00 | 4.00 |
| 203.50 | 235.54 | 248.73 | 3.00 | 4.00 |
| 247.83 | 241.06 | 270.29 | 3.00 | 4.00 |
| 248.92 | 234.48 | 295.34 | 3.00 | 4.00 |
| 227.59 | 261.14 | 251.65 | 3.00 | 4.00 |
| 227.04 | 228.50 | 259.68 | 3.00 | 4.00 |
| 242.10 | 255.85 | 250.94 | 3.00 | 4.00 |
| 205.01 | 220.98 | 262.01 | 3.00 | 4.00 |
| 217.65 | 242.43 | 245.44 | 3.00 | 4.00 |
| 243.35 | 258.14 | 254.39 | 3.00 | 4.00 |
| 247.45 | 226.44 | 281.23 | 3.00 | 4.00 |
| 209.10 | 243.43 | 222.52 | 3.00 | 4.00 |
| 238.00 | 236.87 | 260.97 | 3.00 | 4.00 |
| 226.63 | 247.74 | 258.95 | 3.00 | 4.00 |
| 236.16 | 216.11 | 356.01 | 3.00 | 4.00 |
| 230.99 | 284.75 | 382.90 | 3.00 | 4.00 |
| 229.28 | 240.17 | 451.03 | 3.00 | 4.00 |
| 231.36 | 246.53 | 318.93 | 3.00 | 4.00 |
| 208.68 | 250.10 | 364.69 | 3.00 | 4.00 |
| 220.85 | 262.73 | 369.58 | 3.00 | 4.00 |
| 186.82 | 256.04 | 272.71 | 3.00 | 4.00 |
| 215.85 | 258.36 | 471.73 | 3.00 | 4.00 |
| 221.76 | 242.91 | 393.40 | 3.00 | 4.00 |
| 199.39 | 262.37 | 256.57 | 3.00 | 4.00 |
| 187.94 | 246.56 | 440.11 | 3.00 | 4.00 |
| 221.37 | 231.42 | 300.02 | 3.00 | 4.00 |
| 202.43 | 244.46 | 334.15 | 3.00 | 4.00 |

| | | | | |
|---|---|---|---|---|
| 190.29 | 240.74 | 291.63 | 3.00 | 4.00 |
| 231.08 | 251.84 | 332.13 | 3.00 | 4.00 |
| 177.00 | 214.81 | 299.30 | 3.00 | 4.00 |
| 194.03 | 273.46 | 337.08 | 3.00 | 4.00 |
| 214.79 | 244.29 | 284.73 | 3.00 | 4.00 |
| 247.32 | 244.43 | 324.41 | 3.00 | 4.00 |
| 228.17 | 243.27 | 306.00 | 3.00 | 4.00 |
| 214.01 | 278.69 | 359.49 | 3.00 | 4.00 |
| 238.49 | 227.61 | 328.95 | 3.00 | 4.00 |
| 206.52 | 218.71 | 263.93 | 3.00 | 4.00 |
| 240.94 | 251.30 | 313.71 | 3.00 | 4.00 |
| 198.61 | 276.06 | 323.10 | 3.00 | 4.00 |
| 239.04 | 268.92 | 314.11 | 3.00 | 4.00 |
| 258.67 | 227.54 | 348.64 | 3.00 | 4.00 |
| 176.67 | 227.76 | 256.10 | 3.00 | 4.00 |
| 197.45 | 232.00 | 308.43 | 3.00 | 4.00 |
| 222.07 | 273.07 | 364.62 | 3.00 | 4.00 |
| 247.77 | 274.06 | 265.35 | 3.00 | 4.00 |
| 206.36 | 242.60 | 286.48 | 3.00 | 4.00 |
| 234.04 | 244.51 | 337.35 | 3.00 | 4.00 |
| 184.12 | 217.90 | 288.19 | 3.00 | 4.00 |
| 222.21 | 244.82 | 323.54 | 3.00 | 4.00 |
| 243.50 | 282.05 | 328.95 | 3.00 | 4.00 |
| 213.37 | 251.30 | 322.51 | 3.00 | 4.00 |
| 234.75 | 238.15 | 300.56 | 3.00 | 4.00 |
| 192.80 | 244.30 | 298.20 | 3.00 | 4.00 |
| 216.41 | 237.97 | 341.64 | 3.00 | 4.00 |
| 227.07 | 248.12 | 364.63 | 3.00 | 4.00 |
| 201.23 | 278.82 | 302.00 | 3.00 | 4.00 |
| 228.34 | 232.57 | 378.38 | 3.00 | 4.00 |
| 214.22 | 219.95 | 279.16 | 3.00 | 4.00 |
| 195.75 | 241.24 | 323.47 | 3.00 | 4.00 |
| 216.98 | 263.35 | 302.33 | 3.00 | 4.00 |
| 203.61 | 241.52 | 346.06 | 3.00 | 4.00 |
| 205.35 | 281.12 | 316.23 | 3.00 | 4.00 |
| 215.67 | 254.78 | 398.90 | 3.00 | 4.00 |
| 190.55 | 220.68 | 267.15 | 3.00 | 4.00 |
| 205.66 | 249.08 | 401.87 | 3.00 | 4.00 |
| 207.05 | 270.10 | 320.15 | 3.00 | 4.00 |
| 219.39 | 240.04 | 290.39 | 3.00 | 4.00 |
| 231.33 | 260.26 | 342.10 | 3.00 | 4.00 |
| 206.49 | 285.73 | 464.94 | 3.00 | 4.00 |
| 189.14 | 235.56 | 275.53 | 3.00 | 4.00 |

| | | | | |
|---|---|---|---|---|
| 205.20 | 245.27 | 366.50 | 3.00 | 4.00 |
| 219.18 | 251.53 | 348.64 | 3.00 | 4.00 |
| 225.68 | 249.03 | 281.08 | 3.00 | 4.00 |
| 228.98 | 248.61 | 277.55 | 3.00 | 4.00 |
| 202.85 | 280.06 | 388.33 | 3.00 | 4.00 |
| 209.78 | 249.99 | 347.65 | 3.00 | 4.00 |
| 247.96 | 231.80 | 325.25 | 3.00 | 4.00 |
| 212.82 | 242.76 | 372.51 | 3.00 | 4.00 |
| 212.42 | 252.99 | 279.15 | 3.00 | 4.00 |
| 184.80 | 261.74 | 280.35 | 3.00 | 4.00 |
| 211.54 | 258.97 | 316.36 | 3.00 | 4.00 |
| 223.73 | 230.57 | 334.18 | 3.00 | 4.00 |
| 221.77 | 237.15 | 344.96 | 3.00 | 4.00 |
| 181.74 | 246.74 | 393.17 | 3.00 | 4.00 |
| 224.25 | 252.94 | 246.03 | 3.00 | 4.00 |
| 217.51 | 249.59 | 301.74 | 3.00 | 4.00 |
| 227.15 | 243.20 | 315.92 | 3.00 | 4.00 |
| 196.67 | 247.75 | 288.89 | 3.00 | 4.00 |
| 209.53 | 244.51 | 390.93 | 3.00 | 4.00 |
| 205.20 | 250.30 | 252.52 | 3.00 | 4.00 |
| 217.31 | 256.86 | 286.01 | 3.00 | 4.00 |
| 210.98 | 248.92 | 329.29 | 3.00 | 4.00 |
| 193.41 | 252.18 | 248.19 | 3.00 | 4.00 |
| 242.23 | 250.39 | 371.32 | 3.00 | 4.00 |
| 210.07 | 256.11 | 231.25 | 3.00 | 4.00 |
| 221.40 | 267.13 | 275.57 | 3.00 | 4.00 |
| 203.21 | 217.27 | 300.96 | 3.00 | 4.00 |
| 184.50 | 241.13 | 339.31 | 3.00 | 4.00 |
| 202.71 | 260.25 | 286.15 | 3.00 | 4.00 |
| 170.88 | 239.51 | 259.02 | 3.00 | 4.00 |
| 176.09 | 221.39 | 336.77 | 3.00 | 4.00 |
| 140.67 | 157.94 | 248.26 | 4.00 | 1.00 |
| 132.40 | 141.82 | 233.42 | 4.00 | 1.00 |
| 149.02 | 154.39 | 263.54 | 4.00 | 1.00 |
| 159.58 | 202.60 | 354.44 | 4.00 | 1.00 |
| 160.21 | 164.35 | 291.77 | 4.00 | 1.00 |
| 164.18 | 163.84 | 293.88 | 4.00 | 1.00 |
| 157.61 | 166.44 | 294.24 | 4.00 | 1.00 |
| 172.42 | 169.11 | 317.13 | 4.00 | 1.00 |
| 176.12 | 164.58 | 316.40 | 4.00 | 1.00 |
| 152.44 | 177.90 | 297.13 | 4.00 | 1.00 |
| 233.32 | 188.86 | 390.81 | 4.00 | 1.00 |
| 165.70 | 188.94 | 321.31 | 4.00 | 1.00 |

| | | | | |
|---|---|---|---|---|
| 165.26 | 199.04 | 326.75 | 4.00 | 1.00 |
| 221.25 | 205.68 | 396.92 | 4.00 | 1.00 |
| 228.00 | 228.35 | 400.89 | 4.00 | 1.00 |
| 173.89 | 192.98 | 321.03 | 4.00 | 1.00 |
| 221.35 | 213.15 | 404.61 | 4.00 | 1.00 |
| 224.55 | 218.18 | 395.64 | 4.00 | 1.00 |
| 183.89 | 181.19 | 337.39 | 4.00 | 1.00 |
| 161.50 | 175.81 | 297.96 | 4.00 | 1.00 |
| 220.87 | 221.29 | 405.13 | 4.00 | 1.00 |
| 161.64 | 168.27 | 300.39 | 4.00 | 1.00 |
| 182.07 | 193.45 | 347.33 | 4.00 | 1.00 |
| 231.17 | 280.93 | 476.95 | 4.00 | 1.00 |
| 217.12 | 231.89 | 425.71 | 4.00 | 1.00 |
| 178.33 | 172.75 | 319.86 | 4.00 | 1.00 |
| 220.82 | 224.47 | 404.41 | 4.00 | 1.00 |
| 295.98 | 353.18 | 575.08 | 4.00 | 1.00 |
| 194.12 | 237.12 | 424.56 | 4.00 | 1.00 |
| 186.75 | 203.66 | 342.92 | 4.00 | 1.00 |
| 220.91 | 237.04 | 404.67 | 4.00 | 1.00 |
| 376.68 | 405.88 | 710.37 | 4.00 | 1.00 |
| 260.50 | 273.50 | 482.63 | 4.00 | 1.00 |
| 199.56 | 216.81 | 397.36 | 4.00 | 1.00 |
| 223.12 | 229.89 | 369.25 | 4.00 | 1.00 |
| 336.27 | 373.70 | 634.43 | 4.00 | 1.00 |
| 279.82 | 309.97 | 552.71 | 4.00 | 1.00 |
| 188.30 | 193.06 | 347.57 | 4.00 | 1.00 |
| 220.82 | 256.61 | 445.03 | 4.00 | 1.00 |
| 229.60 | 280.82 | 411.13 | 4.00 | 1.00 |
| 258.26 | 289.76 | 481.11 | 4.00 | 1.00 |
| 281.91 | 299.22 | 556.17 | 4.00 | 1.00 |
| 194.78 | 183.94 | 343.52 | 4.00 | 1.00 |
| 224.67 | 252.48 | 448.14 | 4.00 | 1.00 |
| 206.82 | 240.16 | 354.16 | 4.00 | 1.00 |
| 249.37 | 271.73 | 461.18 | 4.00 | 1.00 |
| 373.55 | 381.59 | 634.17 | 4.00 | 1.00 |
| 197.76 | 177.17 | 335.68 | 4.00 | 1.00 |
| 271.74 | 308.01 | 513.53 | 4.00 | 1.00 |
| 203.44 | 235.11 | 373.86 | 4.00 | 1.00 |
| 207.65 | 244.57 | 384.21 | 4.00 | 1.00 |
| 310.80 | 344.49 | 553.19 | 4.00 | 1.00 |
| 205.27 | 233.03 | 374.97 | 4.00 | 1.00 |
| 199.66 | 209.41 | 375.75 | 4.00 | 1.00 |
| 238.86 | 262.58 | 434.75 | 4.00 | 1.00 |

| | | | | |
|---|---|---|---|---|
| 225.45 | 245.82 | 387.33 | 4.00 | 1.00 |
| 212.62 | 264.32 | 396.73 | 4.00 | 1.00 |
| 257.19 | 285.81 | 437.17 | 4.00 | 1.00 |
| 186.48 | 153.77 | 291.26 | 4.00 | 1.00 |
| 240.73 | 269.53 | 454.87 | 4.00 | 1.00 |
| 291.37 | 290.65 | 517.00 | 4.00 | 1.00 |
| 199.58 | 240.09 | 370.49 | 4.00 | 1.00 |
| 250.65 | 273.17 | 423.12 | 4.00 | 1.00 |
| 184.31 | 186.27 | 323.13 | 4.00 | 1.00 |
| 171.00 | 193.90 | 327.66 | 4.00 | 1.00 |
| 272.69 | 262.33 | 517.30 | 4.00 | 1.00 |
| 211.71 | 239.02 | 395.46 | 4.00 | 1.00 |
| 219.61 | 242.35 | 371.56 | 4.00 | 1.00 |
| 185.29 | 190.57 | 337.45 | 4.00 | 1.00 |
| 169.89 | 187.85 | 307.62 | 4.00 | 1.00 |
| 254.80 | 288.27 | 483.37 | 4.00 | 1.00 |
| 225.11 | 264.97 | 422.02 | 4.00 | 1.00 |
| 191.91 | 204.74 | 350.01 | 4.00 | 1.00 |
| 183.72 | 201.19 | 341.27 | 4.00 | 1.00 |
| 204.07 | 244.66 | 381.29 | 4.00 | 1.00 |
| 331.60 | 383.11 | 636.78 | 4.00 | 1.00 |
| 186.35 | 208.60 | 339.74 | 4.00 | 1.00 |
| 195.27 | 217.66 | 393.34 | 4.00 | 1.00 |
| 203.95 | 221.15 | 384.12 | 4.00 | 1.00 |
| 290.82 | 306.74 | 554.67 | 4.00 | 1.00 |
| 195.08 | 220.23 | 392.61 | 4.00 | 1.00 |
| 271.90 | 275.62 | 487.76 | 4.00 | 1.00 |
| 203.05 | 216.18 | 379.75 | 4.00 | 1.00 |
| 295.30 | 338.73 | 580.87 | 4.00 | 1.00 |
| 200.16 | 222.38 | 390.27 | 4.00 | 1.00 |
| 217.65 | 222.54 | 393.55 | 4.00 | 1.00 |
| 190.12 | 224.52 | 395.84 | 4.00 | 1.00 |
| 247.63 | 273.08 | 474.76 | 4.00 | 1.00 |
| 246.06 | 286.35 | 479.46 | 4.00 | 1.00 |
| 160.69 | 168.16 | 322.42 | 4.00 | 1.00 |
| 284.61 | 291.31 | 575.06 | 4.00 | 1.00 |
| 157.67 | 164.13 | 316.56 | 4.00 | 1.00 |
| 177.54 | 171.33 | 317.34 | 4.00 | 1.00 |
| 266.62 | 324.25 | 634.38 | 4.00 | 1.00 |
| 242.60 | 295.20 | 560.86 | 4.00 | 1.00 |
| 279.37 | 306.44 | 574.39 | 4.00 | 1.00 |
| 204.88 | 201.94 | 436.20 | 4.00 | 1.00 |
| 203.95 | 307.88 | 557.35 | 4.00 | 1.00 |

| | | | | |
|---|---|---|---|---|
| 205.79 | 188.17 | 421.81 | 4.00 | 1.00 |
| 190.92 | 276.85 | 545.88 | 4.00 | 1.00 |
| 241.54 | 249.47 | 510.92 | 4.00 | 1.00 |
| 202.14 | 311.37 | 602.65 | 4.00 | 1.00 |
| 324.72 | 340.59 | 639.11 | 4.00 | 1.00 |
| 277.71 | 327.01 | 647.67 | 4.00 | 1.00 |
| 203.03 | 280.80 | 549.43 | 4.00 | 1.00 |
| 295.37 | 316.18 | 593.95 | 4.00 | 1.00 |
| 232.33 | 360.52 | 681.34 | 4.00 | 1.00 |
| 278.23 | 340.24 | 644.69 | 4.00 | 1.00 |
| 419.24 | 422.83 | 837.03 | 4.00 | 1.00 |
| 195.40 | 260.21 | 497.32 | 4.00 | 1.00 |
| 247.00 | 306.56 | 555.72 | 4.00 | 1.00 |
| 313.05 | 438.13 | 792.48 | 4.00 | 1.00 |
| 269.61 | 307.60 | 615.37 | 4.00 | 1.00 |
| 248.58 | 335.37 | 731.48 | 4.00 | 1.00 |
| 243.27 | 300.06 | 541.95 | 4.00 | 1.00 |
| 249.96 | 304.29 | 566.05 | 4.00 | 1.00 |
| 292.16 | 369.07 | 684.69 | 4.00 | 1.00 |
| 242.11 | 311.84 | 631.30 | 4.00 | 1.00 |
| 235.58 | 275.26 | 509.30 | 4.00 | 1.00 |
| 254.76 | 321.95 | 576.90 | 4.00 | 1.00 |
| 255.25 | 343.43 | 633.84 | 4.00 | 1.00 |
| 211.61 | 271.44 | 496.26 | 4.00 | 1.00 |
| 231.61 | 269.25 | 528.58 | 4.00 | 1.00 |
| 181.99 | 271.58 | 465.46 | 4.00 | 1.00 |
| 283.00 | 342.80 | 606.77 | 4.00 | 1.00 |
| 162.58 | 208.25 | 375.88 | 4.00 | 1.00 |
| 280.44 | 342.69 | 664.28 | 4.00 | 1.00 |
| 331.82 | 334.22 | 655.59 | 4.00 | 1.00 |
| 252.16 | 314.02 | 591.50 | 4.00 | 1.00 |
| 204.70 | 277.58 | 461.96 | 4.00 | 1.00 |
| 227.52 | 282.47 | 509.12 | 4.00 | 1.00 |
| 177.58 | 229.99 | 422.40 | 4.00 | 1.00 |
| 345.43 | 359.66 | 719.39 | 4.00 | 1.00 |
| 231.79 | 280.83 | 519.29 | 4.00 | 1.00 |
| 278.06 | 361.32 | 629.85 | 4.00 | 1.00 |
| 243.83 | 342.96 | 582.94 | 4.00 | 1.00 |
| 170.61 | 244.14 | 397.64 | 4.00 | 1.00 |
| 306.90 | 314.54 | 645.55 | 4.00 | 1.00 |
| 255.87 | 291.99 | 566.99 | 4.00 | 1.00 |
| 193.11 | 244.80 | 450.92 | 4.00 | 1.00 |
| 390.57 | 472.37 | 902.09 | 4.00 | 1.00 |

| | | | | |
|---|---|---|---|---|
| 182.99 | 230.27 | 380.46 | 4.00 | 1.00 |
| 236.17 | 337.07 | 530.25 | 4.00 | 1.00 |
| 378.52 | 422.68 | 867.32 | 4.00 | 1.00 |
| 238.35 | 263.01 | 484.29 | 4.00 | 2.00 |
| 194.55 | 191.17 | 398.66 | 4.00 | 2.00 |
| 168.47 | 162.22 | 328.43 | 4.00 | 2.00 |
| 260.23 | 267.59 | 493.14 | 4.00 | 2.00 |
| 149.32 | 163.53 | 287.77 | 4.00 | 2.00 |
| 216.50 | 223.56 | 414.32 | 4.00 | 2.00 |
| 177.55 | 203.43 | 368.56 | 4.00 | 2.00 |
| 197.45 | 195.87 | 402.31 | 4.00 | 2.00 |
| 196.10 | 195.54 | 355.32 | 4.00 | 2.00 |
| 181.29 | 197.15 | 383.34 | 4.00 | 2.00 |
| 195.56 | 186.74 | 405.76 | 4.00 | 2.00 |
| 207.66 | 194.11 | 374.21 | 4.00 | 2.00 |
| 248.44 | 239.88 | 505.45 | 4.00 | 2.00 |
| 212.50 | 209.00 | 424.20 | 4.00 | 2.00 |
| 162.85 | 190.43 | 363.89 | 4.00 | 2.00 |
| 251.30 | 217.53 | 457.71 | 4.00 | 2.00 |
| 226.23 | 218.32 | 471.14 | 4.00 | 2.00 |
| 191.31 | 196.57 | 374.57 | 4.00 | 2.00 |
| 210.88 | 228.65 | 452.50 | 4.00 | 2.00 |
| 198.34 | 173.46 | 378.86 | 4.00 | 2.00 |
| 262.16 | 234.96 | 494.71 | 4.00 | 2.00 |
| 224.67 | 214.21 | 439.30 | 4.00 | 2.00 |
| 194.27 | 204.87 | 402.40 | 4.00 | 2.00 |
| 296.67 | 264.41 | 565.48 | 4.00 | 2.00 |
| 259.05 | 259.66 | 468.67 | 4.00 | 2.00 |
| 292.47 | 283.58 | 533.53 | 4.00 | 2.00 |
| 237.03 | 232.23 | 470.64 | 4.00 | 2.00 |
| 241.10 | 236.64 | 481.62 | 4.00 | 2.00 |
| 223.58 | 230.72 | 430.05 | 4.00 | 2.00 |
| 290.64 | 279.24 | 540.30 | 4.00 | 2.00 |
| 263.31 | 239.18 | 470.48 | 4.00 | 2.00 |
| 211.57 | 210.44 | 429.65 | 4.00 | 2.00 |
| 188.18 | 170.16 | 389.53 | 4.00 | 2.00 |
| 264.61 | 284.09 | 552.36 | 4.00 | 2.00 |
| 239.97 | 249.74 | 493.40 | 4.00 | 2.00 |
| 279.22 | 249.56 | 470.15 | 4.00 | 2.00 |
| 162.97 | 158.60 | 336.69 | 4.00 | 2.00 |
| 199.18 | 171.08 | 389.36 | 4.00 | 2.00 |
| 286.16 | 302.24 | 592.43 | 4.00 | 2.00 |
| 230.79 | 240.42 | 479.86 | 4.00 | 2.00 |

| | | | | |
|---|---|---|---|---|
| 259.62 | 261.99 | 568.15 | 4.00 | 2.00 |
| 183.55 | 165.33 | 333.72 | 4.00 | 2.00 |
| 169.27 | 165.00 | 334.75 | 4.00 | 2.00 |
| 314.89 | 276.69 | 581.55 | 4.00 | 2.00 |
| 227.28 | 247.05 | 484.41 | 4.00 | 2.00 |
| 311.71 | 301.16 | 645.00 | 4.00 | 2.00 |
| 229.91 | 192.80 | 388.53 | 4.00 | 2.00 |
| 164.02 | 145.00 | 312.74 | 4.00 | 2.00 |
| 245.96 | 188.21 | 438.26 | 4.00 | 2.00 |
| 205.05 | 232.50 | 447.91 | 4.00 | 2.00 |
| 225.13 | 230.29 | 449.41 | 4.00 | 2.00 |
| 324.94 | 279.71 | 588.09 | 4.00 | 2.00 |
| 243.37 | 219.49 | 501.71 | 4.00 | 2.00 |
| 204.40 | 193.61 | 379.14 | 4.00 | 2.00 |
| 292.61 | 225.37 | 513.73 | 4.00 | 2.00 |
| 185.89 | 213.73 | 393.19 | 4.00 | 2.00 |
| 458.25 | 452.36 | 918.93 | 4.00 | 2.00 |
| 231.73 | 244.62 | 506.85 | 4.00 | 2.00 |
| 226.85 | 203.65 | 435.62 | 4.00 | 2.00 |
| 148.04 | 165.56 | 325.11 | 4.00 | 2.00 |
| 261.97 | 253.53 | 509.68 | 4.00 | 2.00 |
| 245.71 | 230.22 | 478.13 | 4.00 | 2.00 |
| 241.27 | 302.76 | 558.08 | 4.00 | 2.00 |
| 288.49 | 232.28 | 486.14 | 4.00 | 2.00 |
| 204.27 | 238.78 | 457.87 | 4.00 | 2.00 |
| 153.99 | 160.52 | 317.21 | 4.00 | 2.00 |
| 175.70 | 205.65 | 408.97 | 4.00 | 2.00 |
| 219.12 | 241.28 | 452.01 | 4.00 | 2.00 |
| 225.81 | 245.02 | 464.59 | 4.00 | 2.00 |
| 178.89 | 182.93 | 400.04 | 4.00 | 2.00 |
| 188.90 | 205.17 | 378.62 | 4.00 | 2.00 |
| 159.16 | 181.27 | 352.68 | 4.00 | 2.00 |
| 175.04 | 229.92 | 456.23 | 4.00 | 2.00 |
| 173.04 | 194.23 | 420.06 | 4.00 | 2.00 |
| 138.81 | 160.30 | 336.00 | 4.00 | 2.00 |
| 193.38 | 248.52 | 498.28 | 4.00 | 2.00 |
| 156.78 | 215.27 | 426.41 | 4.00 | 2.00 |
| 143.00 | 160.80 | 305.14 | 4.00 | 2.00 |
| 142.60 | 231.39 | 451.89 | 4.00 | 2.00 |
| 125.91 | 172.26 | 336.32 | 4.00 | 2.00 |
| 197.49 | 228.90 | 434.87 | 4.00 | 2.00 |
| 146.57 | 203.56 | 398.10 | 4.00 | 2.00 |
| 164.76 | 192.37 | 377.65 | 4.00 | 2.00 |

| | | | | |
|---|---|---|---|---|
| 159.46 | 201.36 | 370.43 | 4.00 | 2.00 |
| 179.82 | 272.34 | 540.02 | 4.00 | 2.00 |
| 150.13 | 167.34 | 317.69 | 4.00 | 2.00 |
| 128.94 | 149.88 | 310.40 | 4.00 | 2.00 |
| 202.35 | 264.57 | 536.56 | 4.00 | 2.00 |
| 160.78 | 196.95 | 369.23 | 4.00 | 2.00 |
| 122.31 | 159.14 | 287.07 | 4.00 | 2.00 |
| 142.03 | 193.86 | 363.81 | 4.00 | 2.00 |
| 176.80 | 235.30 | 437.66 | 4.00 | 2.00 |
| 119.10 | 157.54 | 302.92 | 4.00 | 2.00 |
| 155.54 | 193.96 | 375.03 | 4.00 | 2.00 |
| 141.63 | 177.94 | 330.23 | 4.00 | 2.00 |
| 211.24 | 280.20 | 523.22 | 4.00 | 2.00 |
| 164.46 | 190.05 | 382.44 | 4.00 | 2.00 |
| 177.58 | 206.53 | 415.75 | 4.00 | 2.00 |
| 177.60 | 236.74 | 425.60 | 4.00 | 2.00 |
| 190.90 | 226.79 | 420.82 | 4.00 | 2.00 |
| 124.90 | 166.28 | 308.65 | 4.00 | 2.00 |
| 213.95 | 222.02 | 457.22 | 4.00 | 2.00 |
| 117.89 | 173.96 | 304.84 | 4.00 | 2.00 |
| 266.21 | 318.62 | 598.04 | 4.00 | 2.00 |
| 173.52 | 227.75 | 450.50 | 4.00 | 2.00 |
| 143.81 | 176.18 | 347.85 | 4.00 | 2.00 |
| 181.84 | 172.39 | 363.52 | 4.00 | 2.00 |
| 152.22 | 185.99 | 365.52 | 4.00 | 2.00 |
| 372.38 | 472.63 | 937.03 | 4.00 | 2.00 |
| 145.45 | 240.89 | 433.15 | 4.00 | 2.00 |
| 159.23 | 219.89 | 407.49 | 4.00 | 2.00 |
| 190.26 | 227.26 | 395.86 | 4.00 | 2.00 |
| 163.52 | 175.04 | 345.67 | 4.00 | 2.00 |
| 253.03 | 315.42 | 626.92 | 4.00 | 2.00 |
| 251.41 | 307.78 | 593.06 | 4.00 | 2.00 |
| 152.72 | 181.33 | 385.06 | 4.00 | 2.00 |
| 188.81 | 206.77 | 409.32 | 4.00 | 2.00 |
| 211.15 | 202.50 | 416.00 | 4.00 | 2.00 |
| 339.18 | 429.70 | 737.41 | 4.00 | 2.00 |
| 296.89 | 377.14 | 756.45 | 4.00 | 2.00 |
| 171.36 | 208.13 | 453.54 | 4.00 | 2.00 |
| 204.78 | 279.07 | 541.22 | 4.00 | 2.00 |
| 148.74 | 184.62 | 311.70 | 4.00 | 2.00 |
| 199.84 | 230.10 | 440.00 | 4.00 | 2.00 |
| 224.39 | 235.06 | 454.16 | 4.00 | 2.00 |
| 232.56 | 294.62 | 565.92 | 4.00 | 2.00 |

| | | | | |
|---|---|---|---|---|
| 231.07 | 258.85 | 450.21 | 4.00 | 2.00 |
| 161.63 | 180.68 | 396.18 | 4.00 | 2.00 |
| 171.04 | 177.93 | 338.64 | 4.00 | 2.00 |
| 235.65 | 308.78 | 557.07 | 4.00 | 2.00 |
| 194.73 | 202.82 | 412.19 | 4.00 | 2.00 |
| 219.89 | 288.62 | 552.52 | 4.00 | 2.00 |
| 246.59 | 252.10 | 498.56 | 4.00 | 2.00 |
| 141.83 | 165.51 | 355.50 | 4.00 | 2.00 |
| 260.94 | 282.95 | 527.30 | 4.00 | 2.00 |
| 197.44 | 223.47 | 469.14 | 4.00 | 2.00 |
| 171.14 | 217.51 | 431.21 | 4.00 | 2.00 |
| 185.80 | 232.61 | 457.90 | 4.00 | 2.00 |
| 187.93 | 214.23 | 405.42 | 4.00 | 2.00 |
| 237.57 | 242.79 | 500.06 | 4.00 | 2.00 |
| 179.80 | 220.85 | 383.02 | 4.00 | 2.00 |
| 215.03 | 205.31 | 392.85 | 4.00 | 3.00 |
| 189.05 | 189.90 | 347.11 | 4.00 | 3.00 |
| 196.52 | 191.80 | 365.34 | 4.00 | 3.00 |
| 182.51 | 183.90 | 395.06 | 4.00 | 3.00 |
| 229.97 | 198.39 | 389.33 | 4.00 | 3.00 |
| 141.58 | 161.42 | 339.73 | 4.00 | 3.00 |
| 217.59 | 183.11 | 373.55 | 4.00 | 3.00 |
| 137.33 | 142.20 | 315.45 | 4.00 | 3.00 |
| 146.28 | 142.98 | 292.46 | 4.00 | 3.00 |
| 193.72 | 169.00 | 353.20 | 4.00 | 3.00 |
| 141.77 | 166.41 | 345.18 | 4.00 | 3.00 |
| 148.35 | 153.44 | 302.87 | 4.00 | 3.00 |
| 154.53 | 159.86 | 295.61 | 4.00 | 3.00 |
| 143.14 | 165.60 | 336.26 | 4.00 | 3.00 |
| 166.90 | 159.37 | 322.85 | 4.00 | 3.00 |
| 147.86 | 174.56 | 345.58 | 4.00 | 3.00 |
| 156.45 | 178.49 | 310.30 | 4.00 | 3.00 |
| 170.02 | 186.31 | 370.77 | 4.00 | 3.00 |
| 205.49 | 190.24 | 383.19 | 4.00 | 3.00 |
| 154.92 | 168.95 | 341.02 | 4.00 | 3.00 |
| 167.13 | 183.31 | 341.31 | 4.00 | 3.00 |
| 166.63 | 167.96 | 336.81 | 4.00 | 3.00 |
| 226.57 | 210.04 | 401.23 | 4.00 | 3.00 |
| 182.07 | 186.75 | 380.82 | 4.00 | 3.00 |
| 152.22 | 177.70 | 347.20 | 4.00 | 3.00 |
| 153.15 | 154.60 | 310.06 | 4.00 | 3.00 |
| 209.55 | 220.91 | 414.32 | 4.00 | 3.00 |
| 247.31 | 238.17 | 452.46 | 4.00 | 3.00 |

| | | | | |
|---|---|---|---|---|
| 212.69 | 222.35 | 464.79 | 4.00 | 3.00 |
| 160.28 | 203.43 | 356.53 | 4.00 | 3.00 |
| 191.00 | 207.78 | 399.36 | 4.00 | 3.00 |
| 212.44 | 195.74 | 419.95 | 4.00 | 3.00 |
| 288.82 | 291.05 | 550.03 | 4.00 | 3.00 |
| 202.89 | 210.04 | 441.09 | 4.00 | 3.00 |
| 248.32 | 259.46 | 477.03 | 4.00 | 3.00 |
| 209.29 | 194.33 | 382.47 | 4.00 | 3.00 |
| 199.65 | 194.63 | 409.56 | 4.00 | 3.00 |
| 219.00 | 217.11 | 407.08 | 4.00 | 3.00 |
| 227.54 | 251.81 | 514.74 | 4.00 | 3.00 |
| 192.96 | 220.82 | 435.68 | 4.00 | 3.00 |
| 191.18 | 209.46 | 402.66 | 4.00 | 3.00 |
| 254.51 | 220.81 | 479.04 | 4.00 | 3.00 |
| 239.12 | 214.14 | 458.16 | 4.00 | 3.00 |
| 213.51 | 217.59 | 406.69 | 4.00 | 3.00 |
| 192.72 | 184.53 | 416.43 | 4.00 | 3.00 |
| 217.07 | 254.31 | 467.77 | 4.00 | 3.00 |
| 196.41 | 185.51 | 372.74 | 4.00 | 3.00 |
| 185.57 | 162.77 | 390.43 | 4.00 | 3.00 |
| 262.67 | 263.41 | 511.02 | 4.00 | 3.00 |
| 235.03 | 257.16 | 471.55 | 4.00 | 3.00 |
| 155.11 | 179.42 | 349.62 | 4.00 | 3.00 |
| 141.90 | 178.94 | 340.74 | 4.00 | 3.00 |
| 177.51 | 189.69 | 412.33 | 4.00 | 3.00 |
| 267.88 | 216.72 | 512.44 | 4.00 | 3.00 |
| 183.27 | 198.80 | 380.07 | 4.00 | 3.00 |
| 194.26 | 171.39 | 392.18 | 4.00 | 3.00 |
| 237.75 | 243.16 | 500.89 | 4.00 | 3.00 |
| 122.56 | 159.41 | 311.51 | 4.00 | 3.00 |
| 171.40 | 199.23 | 409.40 | 4.00 | 3.00 |
| 196.17 | 174.50 | 421.34 | 4.00 | 3.00 |
| 173.22 | 190.47 | 386.08 | 4.00 | 3.00 |
| 202.97 | 178.99 | 403.91 | 4.00 | 3.00 |
| 224.29 | 226.55 | 450.02 | 4.00 | 3.00 |
| 167.57 | 188.38 | 359.12 | 4.00 | 3.00 |
| 157.05 | 183.00 | 354.08 | 4.00 | 3.00 |
| 213.15 | 221.79 | 444.19 | 4.00 | 3.00 |
| 168.14 | 164.88 | 361.66 | 4.00 | 3.00 |
| 208.61 | 199.69 | 405.82 | 4.00 | 3.00 |
| 185.15 | 193.53 | 377.62 | 4.00 | 3.00 |
| 164.25 | 196.54 | 382.78 | 4.00 | 3.00 |
| 224.33 | 266.90 | 495.14 | 4.00 | 3.00 |

| | | | | |
|---|---|---|---|---|
| 208.06 | 207.64 | 431.44 | 4.00 | 3.00 |
| 202.41 | 226.32 | 416.41 | 4.00 | 3.00 |
| 212.88 | 188.87 | 378.95 | 4.00 | 3.00 |
| 171.42 | 182.94 | 366.34 | 4.00 | 3.00 |
| 240.96 | 254.47 | 491.69 | 4.00 | 3.00 |
| 258.98 | 283.33 | 525.20 | 4.00 | 3.00 |
| 208.15 | 201.58 | 423.44 | 4.00 | 3.00 |
| 188.31 | 188.54 | 352.89 | 4.00 | 3.00 |
| 180.17 | 213.64 | 394.16 | 4.00 | 3.00 |
| 233.51 | 223.80 | 436.58 | 4.00 | 3.00 |
| 177.05 | 193.09 | 369.38 | 4.00 | 3.00 |
| 171.19 | 175.26 | 334.53 | 4.00 | 3.00 |
| 161.22 | 133.12 | 306.09 | 4.00 | 3.00 |
| 148.86 | 168.67 | 339.94 | 4.00 | 3.00 |
| 177.18 | 227.89 | 426.29 | 4.00 | 3.00 |
| 143.38 | 152.66 | 339.96 | 4.00 | 3.00 |
| 173.61 | 164.96 | 332.45 | 4.00 | 3.00 |
| 183.88 | 175.70 | 362.72 | 4.00 | 3.00 |
| 168.36 | 157.20 | 340.83 | 4.00 | 3.00 |
| 151.94 | 241.21 | 395.75 | 4.00 | 3.00 |
| 158.64 | 213.36 | 399.65 | 4.00 | 3.00 |
| 153.60 | 218.93 | 418.57 | 4.00 | 3.00 |
| 132.73 | 207.88 | 356.37 | 4.00 | 3.00 |
| 155.95 | 213.36 | 393.42 | 4.00 | 3.00 |
| 167.69 | 263.37 | 430.57 | 4.00 | 3.00 |
| 190.65 | 272.22 | 464.52 | 4.00 | 3.00 |
| 165.15 | 217.15 | 387.31 | 4.00 | 3.00 |
| 214.10 | 277.67 | 510.24 | 4.00 | 3.00 |
| 174.47 | 267.85 | 484.04 | 4.00 | 3.00 |
| 153.73 | 215.63 | 379.29 | 4.00 | 3.00 |
| 200.22 | 254.12 | 434.40 | 4.00 | 3.00 |
| 181.63 | 228.75 | 441.52 | 4.00 | 3.00 |
| 156.57 | 232.66 | 398.77 | 4.00 | 3.00 |
| 206.31 | 260.54 | 430.55 | 4.00 | 3.00 |
| 205.80 | 233.34 | 453.04 | 4.00 | 3.00 |
| 160.61 | 219.54 | 375.64 | 4.00 | 3.00 |
| 185.40 | 263.23 | 437.49 | 4.00 | 3.00 |
| 193.06 | 247.26 | 456.59 | 4.00 | 3.00 |
| 196.80 | 263.03 | 433.72 | 4.00 | 3.00 |
| 175.69 | 188.31 | 354.56 | 4.00 | 3.00 |
| 210.00 | 262.94 | 443.81 | 4.00 | 3.00 |
| 208.44 | 295.56 | 517.13 | 4.00 | 3.00 |
| 197.97 | 266.86 | 432.07 | 4.00 | 3.00 |

| | | | | |
|---|---|---|---|---|
| 143.46 | 186.14 | 314.36 | 4.00 | 3.00 |
| 217.47 | 260.04 | 450.59 | 4.00 | 3.00 |
| 161.92 | 226.12 | 374.65 | 4.00 | 3.00 |
| 238.24 | 264.07 | 502.32 | 4.00 | 3.00 |
| 176.79 | 226.56 | 373.18 | 4.00 | 3.00 |
| 122.58 | 168.70 | 301.21 | 4.00 | 3.00 |
| 151.98 | 206.66 | 346.27 | 4.00 | 3.00 |
| 232.65 | 275.45 | 483.19 | 4.00 | 3.00 |
| 168.20 | 194.68 | 340.83 | 4.00 | 3.00 |
| 138.13 | 176.25 | 308.80 | 4.00 | 3.00 |
| 122.88 | 185.96 | 313.30 | 4.00 | 3.00 |
| 156.96 | 219.91 | 389.54 | 4.00 | 3.00 |
| 212.73 | 283.51 | 487.29 | 4.00 | 3.00 |
| 148.01 | 173.79 | 298.57 | 4.00 | 3.00 |
| 146.94 | 183.21 | 322.07 | 4.00 | 3.00 |
| 133.47 | 227.00 | 357.50 | 4.00 | 3.00 |
| 128.96 | 178.31 | 332.24 | 4.00 | 3.00 |
| 152.85 | 213.04 | 387.32 | 4.00 | 3.00 |
| 131.96 | 180.74 | 311.14 | 4.00 | 3.00 |
| 143.72 | 171.50 | 294.93 | 4.00 | 3.00 |
| 153.60 | 186.16 | 325.27 | 4.00 | 3.00 |
| 123.77 | 220.89 | 343.96 | 4.00 | 3.00 |
| 148.77 | 193.56 | 350.09 | 4.00 | 3.00 |
| 148.90 | 192.07 | 332.27 | 4.00 | 3.00 |
| 113.26 | 158.51 | 280.69 | 4.00 | 3.00 |
| 150.06 | 181.77 | 315.23 | 4.00 | 3.00 |
| 188.76 | 229.18 | 418.51 | 4.00 | 3.00 |
| 120.35 | 174.74 | 319.85 | 4.00 | 3.00 |
| 151.82 | 195.86 | 358.39 | 4.00 | 3.00 |
| 138.03 | 209.29 | 350.43 | 4.00 | 3.00 |
| 126.23 | 161.43 | 292.19 | 4.00 | 3.00 |
| 162.13 | 205.76 | 374.84 | 4.00 | 3.00 |
| 120.08 | 178.32 | 312.31 | 4.00 | 3.00 |
| 136.12 | 186.50 | 333.24 | 4.00 | 3.00 |
| 166.28 | 220.16 | 366.49 | 4.00 | 3.00 |
| 153.37 | 209.45 | 377.69 | 4.00 | 3.00 |
| 131.51 | 164.03 | 292.25 | 4.00 | 3.00 |
| 155.84 | 191.56 | 348.08 | 4.00 | 3.00 |
| 186.05 | 226.66 | 409.58 | 4.00 | 3.00 |
| 180.34 | 234.21 | 388.99 | 4.00 | 3.00 |
| 120.26 | 162.91 | 286.83 | 4.00 | 3.00 |
| 176.11 | 232.76 | 429.89 | 4.00 | 3.00 |
| 198.43 | 221.17 | 402.40 | 4.00 | 3.00 |

| | | | | |
|---|---|---|---|---|
| 195.53 | 234.38 | 401.07 | 4.00 | 3.00 |
| 222.49 | 293.72 | 536.14 | 4.00 | 3.00 |
| 200.77 | 242.71 | 437.06 | 4.00 | 3.00 |
| 186.90 | 229.95 | 410.51 | 4.00 | 3.00 |
| 247.26 | 302.57 | 512.84 | 4.00 | 3.00 |
| 151.51 | 202.51 | 372.26 | 4.00 | 3.00 |
| 188.54 | 261.69 | 473.86 | 4.00 | 3.00 |
| 182.26 | 224.20 | 404.59 | 4.00 | 3.00 |
| 201.82 | 278.16 | 491.07 | 4.00 | 3.00 |
| 170.08 | 251.78 | 428.64 | 4.00 | 3.00 |
| 175.31 | 245.14 | 451.48 | 4.00 | 3.00 |
| 211.22 | 308.79 | 549.07 | 4.00 | 3.00 |
| 216.95 | 197.21 | 238.28 | 4.00 | 4.00 |
| 213.48 | 195.67 | 250.38 | 4.00 | 4.00 |
| 225.74 | 194.80 | 242.50 | 4.00 | 4.00 |
| 207.57 | 211.14 | 210.62 | 4.00 | 4.00 |
| 233.18 | 198.04 | 265.55 | 4.00 | 4.00 |
| 222.78 | 208.91 | 221.90 | 4.00 | 4.00 |
| 243.21 | 211.49 | 263.44 | 4.00 | 4.00 |
| 230.22 | 221.09 | 231.76 | 4.00 | 4.00 |
| 195.94 | 217.09 | 260.74 | 4.00 | 4.00 |
| 242.20 | 209.86 | 252.16 | 4.00 | 4.00 |
| 225.36 | 230.55 | 251.92 | 4.00 | 4.00 |
| 199.18 | 219.69 | 258.76 | 4.00 | 4.00 |
| 218.92 | 205.56 | 246.69 | 4.00 | 4.00 |
| 220.93 | 253.52 | 248.84 | 4.00 | 4.00 |
| 215.99 | 226.16 | 237.36 | 4.00 | 4.00 |
| 196.49 | 216.72 | 285.56 | 4.00 | 4.00 |
| 212.56 | 189.49 | 227.87 | 4.00 | 4.00 |
| 217.86 | 250.63 | 253.90 | 4.00 | 4.00 |
| 233.93 | 240.30 | 232.18 | 4.00 | 4.00 |
| 209.51 | 229.14 | 265.71 | 4.00 | 4.00 |
| 202.94 | 194.18 | 204.28 | 4.00 | 4.00 |
| 233.03 | 265.95 | 314.18 | 4.00 | 4.00 |
| 224.57 | 239.35 | 222.24 | 4.00 | 4.00 |
| 233.23 | 216.40 | 275.57 | 4.00 | 4.00 |
| 194.06 | 209.37 | 205.91 | 4.00 | 4.00 |
| 225.30 | 229.50 | 293.69 | 4.00 | 4.00 |
| 210.04 | 256.91 | 221.32 | 4.00 | 4.00 |
| 236.99 | 230.42 | 232.89 | 4.00 | 4.00 |
| 221.68 | 209.77 | 218.47 | 4.00 | 4.00 |
| 192.86 | 207.08 | 251.78 | 4.00 | 4.00 |
| 217.42 | 244.40 | 253.68 | 4.00 | 4.00 |

| | | | | |
|---|---|---|---|---|
| 205.11 | 264.01 | 222.33 | 4.00 | 4.00 |
| 220.99 | 217.34 | 235.61 | 4.00 | 4.00 |
| 224.04 | 206.87 | 214.09 | 4.00 | 4.00 |
| 179.44 | 202.11 | 271.84 | 4.00 | 4.00 |
| 211.47 | 215.68 | 249.00 | 4.00 | 4.00 |
| 202.86 | 247.62 | 231.54 | 4.00 | 4.00 |
| 213.63 | 241.98 | 238.58 | 4.00 | 4.00 |
| 218.87 | 222.48 | 250.85 | 4.00 | 4.00 |
| 213.30 | 203.56 | 252.98 | 4.00 | 4.00 |
| 201.73 | 212.73 | 252.69 | 4.00 | 4.00 |
| 203.75 | 230.10 | 225.23 | 4.00 | 4.00 |
| 205.62 | 239.52 | 259.48 | 4.00 | 4.00 |
| 224.90 | 229.14 | 258.08 | 4.00 | 4.00 |
| 223.98 | 226.09 | 271.82 | 4.00 | 4.00 |
| 215.91 | 206.98 | 229.12 | 4.00 | 4.00 |
| 218.26 | 229.82 | 234.94 | 4.00 | 4.00 |
| 201.01 | 222.79 | 269.77 | 4.00 | 4.00 |
| 202.30 | 227.79 | 285.85 | 4.00 | 4.00 |
| 250.21 | 239.05 | 224.59 | 4.00 | 4.00 |
| 248.10 | 218.38 | 237.29 | 4.00 | 4.00 |
| 229.21 | 229.58 | 276.69 | 4.00 | 4.00 |
| 206.83 | 233.44 | 277.80 | 4.00 | 4.00 |
| 221.99 | 228.57 | 231.66 | 4.00 | 4.00 |
| 200.56 | 259.20 | 245.47 | 4.00 | 4.00 |
| 202.02 | 234.82 | 271.80 | 4.00 | 4.00 |
| 228.18 | 242.48 | 272.19 | 4.00 | 4.00 |
| 244.43 | 227.46 | 261.43 | 4.00 | 4.00 |
| 230.95 | 238.80 | 271.24 | 4.00 | 4.00 |
| 209.36 | 231.23 | 242.40 | 4.00 | 4.00 |
| 238.77 | 223.84 | 265.07 | 4.00 | 4.00 |
| 203.74 | 260.20 | 292.40 | 4.00 | 4.00 |
| 206.43 | 238.09 | 242.56 | 4.00 | 4.00 |
| 252.06 | 237.00 | 258.16 | 4.00 | 4.00 |
| 246.68 | 239.49 | 276.80 | 4.00 | 4.00 |
| 212.26 | 258.29 | 252.57 | 4.00 | 4.00 |
| 227.17 | 228.32 | 289.82 | 4.00 | 4.00 |
| 235.99 | 249.06 | 257.32 | 4.00 | 4.00 |
| 197.18 | 203.54 | 249.82 | 4.00 | 4.00 |
| 216.77 | 234.68 | 263.86 | 4.00 | 4.00 |
| 245.28 | 256.61 | 250.77 | 4.00 | 4.00 |
| 256.42 | 232.68 | 264.86 | 4.00 | 4.00 |
| 208.15 | 247.07 | 243.86 | 4.00 | 4.00 |
| 244.78 | 235.29 | 228.56 | 4.00 | 4.00 |

| | | | | |
|---|---|---|---|---|
| 225.44 | 254.64 | 237.04 | 4.00 | 4.00 |
| 234.11 | 245.99 | 396.64 | 4.00 | 4.00 |
| 231.52 | 263.95 | 385.06 | 4.00 | 4.00 |
| 220.90 | 242.80 | 404.77 | 4.00 | 4.00 |
| 232.47 | 239.78 | 358.22 | 4.00 | 4.00 |
| 198.18 | 266.35 | 406.11 | 4.00 | 4.00 |
| 218.65 | 256.00 | 350.59 | 4.00 | 4.00 |
| 201.90 | 255.01 | 294.59 | 4.00 | 4.00 |
| 216.84 | 264.17 | 425.79 | 4.00 | 4.00 |
| 191.98 | 254.58 | 355.61 | 4.00 | 4.00 |
| 210.34 | 248.07 | 287.48 | 4.00 | 4.00 |
| 197.97 | 249.94 | 400.12 | 4.00 | 4.00 |
| 256.05 | 231.14 | 289.51 | 4.00 | 4.00 |
| 205.70 | 261.35 | 305.50 | 4.00 | 4.00 |
| 199.26 | 248.39 | 325.65 | 4.00 | 4.00 |
| 261.25 | 245.59 | 296.80 | 4.00 | 4.00 |
| 212.24 | 225.20 | 271.05 | 4.00 | 4.00 |
| 197.32 | 258.87 | 329.69 | 4.00 | 4.00 |
| 218.86 | 231.86 | 297.45 | 4.00 | 4.00 |
| 219.12 | 249.51 | 337.59 | 4.00 | 4.00 |
| 233.88 | 239.83 | 276.96 | 4.00 | 4.00 |
| 227.84 | 271.81 | 323.16 | 4.00 | 4.00 |
| 242.27 | 221.77 | 310.88 | 4.00 | 4.00 |
| 207.27 | 220.78 | 268.13 | 4.00 | 4.00 |
| 250.26 | 255.64 | 369.66 | 4.00 | 4.00 |
| 210.85 | 254.04 | 297.42 | 4.00 | 4.00 |
| 244.93 | 257.71 | 345.36 | 4.00 | 4.00 |
| 207.68 | 242.90 | 367.04 | 4.00 | 4.00 |
| 201.90 | 212.08 | 274.90 | 4.00 | 4.00 |
| 189.09 | 244.59 | 280.71 | 4.00 | 4.00 |
| 226.31 | 293.77 | 351.14 | 4.00 | 4.00 |
| 260.90 | 283.51 | 266.28 | 4.00 | 4.00 |
| 205.89 | 237.39 | 309.81 | 4.00 | 4.00 |
| 240.29 | 266.06 | 374.08 | 4.00 | 4.00 |
| 187.22 | 199.67 | 288.50 | 4.00 | 4.00 |
| 231.94 | 273.37 | 292.04 | 4.00 | 4.00 |
| 213.95 | 281.21 | 329.81 | 4.00 | 4.00 |
| 222.21 | 225.12 | 320.21 | 4.00 | 4.00 |
| 235.28 | 237.28 | 304.37 | 4.00 | 4.00 |
| 195.40 | 260.42 | 280.01 | 4.00 | 4.00 |
| 223.63 | 217.30 | 303.90 | 4.00 | 4.00 |
| 204.26 | 272.83 | 328.74 | 4.00 | 4.00 |
| 208.53 | 282.86 | 316.03 | 4.00 | 4.00 |

| | | | | |
|---|---|---|---|---|
| 234.21 | 201.06 | 376.20 | 4.00 | 4.00 |
| 195.01 | 224.73 | 301.74 | 4.00 | 4.00 |
| 201.92 | 253.62 | 312.39 | 4.00 | 4.00 |
| 220.11 | 250.30 | 318.15 | 4.00 | 4.00 |
| 199.33 | 255.78 | 360.17 | 4.00 | 4.00 |
| 203.65 | 281.67 | 324.93 | 4.00 | 4.00 |
| 218.57 | 224.55 | 370.63 | 4.00 | 4.00 |
| 180.00 | 226.29 | 287.18 | 4.00 | 4.00 |
| 203.70 | 248.72 | 358.37 | 4.00 | 4.00 |
| 211.81 | 270.15 | 311.84 | 4.00 | 4.00 |
| 209.36 | 239.95 | 320.56 | 4.00 | 4.00 |
| 208.46 | 270.66 | 367.97 | 4.00 | 4.00 |
| 203.92 | 279.66 | 440.13 | 4.00 | 4.00 |
| 195.90 | 227.99 | 284.87 | 4.00 | 4.00 |
| 209.47 | 241.51 | 375.33 | 4.00 | 4.00 |
| 217.82 | 271.51 | 332.97 | 4.00 | 4.00 |
| 208.60 | 247.46 | 281.90 | 4.00 | 4.00 |
| 206.11 | 262.00 | 353.09 | 4.00 | 4.00 |
| 203.38 | 276.41 | 434.22 | 4.00 | 4.00 |
| 210.36 | 237.25 | 306.47 | 4.00 | 4.00 |
| 274.56 | 232.58 | 342.65 | 4.00 | 4.00 |
| 226.39 | 254.52 | 369.52 | 4.00 | 4.00 |
| 208.24 | 252.68 | 263.58 | 4.00 | 4.00 |
| 180.69 | 282.62 | 284.27 | 4.00 | 4.00 |
| 248.30 | 257.26 | 325.93 | 4.00 | 4.00 |
| 233.40 | 224.29 | 312.64 | 4.00 | 4.00 |
| 210.95 | 253.83 | 394.46 | 4.00 | 4.00 |
| 178.59 | 255.38 | 321.23 | 4.00 | 4.00 |
| 223.75 | 251.36 | 248.77 | 4.00 | 4.00 |
| 217.68 | 250.35 | 293.67 | 4.00 | 4.00 |
| 209.74 | 233.50 | 344.10 | 4.00 | 4.00 |
| 197.38 | 244.43 | 270.38 | 4.00 | 4.00 |
| 208.45 | 251.94 | 388.66 | 4.00 | 4.00 |
| 215.67 | 248.75 | 237.78 | 4.00 | 4.00 |
| 213.43 | 258.55 | 278.66 | 4.00 | 4.00 |
| 211.63 | 231.66 | 316.22 | 4.00 | 4.00 |
| 202.71 | 253.75 | 259.80 | 4.00 | 4.00 |
| 246.79 | 249.52 | 359.03 | 4.00 | 4.00 |
| 207.88 | 257.58 | 227.35 | 4.00 | 4.00 |
| 227.74 | 271.91 | 303.09 | 4.00 | 4.00 |
| 190.87 | 210.81 | 312.25 | 4.00 | 4.00 |
| 201.49 | 245.30 | 368.54 | 4.00 | 4.00 |
| 192.69 | 246.74 | 257.64 | 4.00 | 4.00 |

| | | | | |
|---|---|---|---|---|
| 190.78 | 264.57 | 263.14 | 4.00 | 4.00 |
| 215.28 | 223.98 | 389.65 | 5.00 | 1.00 |
| 213.93 | 226.10 | 407.62 | 5.00 | 1.00 |
| 147.75 | 157.18 | 259.11 | 5.00 | 1.00 |
| 139.67 | 161.19 | 293.20 | 5.00 | 1.00 |
| 183.56 | 184.67 | 328.96 | 5.00 | 1.00 |
| 163.92 | 170.19 | 292.35 | 5.00 | 1.00 |
| 157.03 | 178.23 | 298.52 | 5.00 | 1.00 |
| 176.22 | 173.57 | 314.35 | 5.00 | 1.00 |
| 166.32 | 166.34 | 311.84 | 5.00 | 1.00 |
| 159.36 | 190.63 | 311.49 | 5.00 | 1.00 |
| 223.39 | 181.20 | 377.53 | 5.00 | 1.00 |
| 167.67 | 178.82 | 321.39 | 5.00 | 1.00 |
| 165.03 | 179.92 | 305.61 | 5.00 | 1.00 |
| 224.05 | 186.89 | 391.93 | 5.00 | 1.00 |
| 200.59 | 213.69 | 367.92 | 5.00 | 1.00 |
| 174.58 | 192.67 | 315.71 | 5.00 | 1.00 |
| 249.70 | 224.67 | 456.06 | 5.00 | 1.00 |
| 213.66 | 207.95 | 374.74 | 5.00 | 1.00 |
| 167.14 | 176.09 | 322.29 | 5.00 | 1.00 |
| 179.40 | 199.39 | 346.15 | 5.00 | 1.00 |
| 241.09 | 236.48 | 421.96 | 5.00 | 1.00 |
| 161.48 | 166.77 | 307.66 | 5.00 | 1.00 |
| 181.31 | 204.06 | 344.33 | 5.00 | 1.00 |
| 220.86 | 264.36 | 459.40 | 5.00 | 1.00 |
| 206.29 | 213.25 | 393.70 | 5.00 | 1.00 |
| 164.53 | 173.17 | 309.37 | 5.00 | 1.00 |
| 265.15 | 286.28 | 490.09 | 5.00 | 1.00 |
| 279.22 | 312.21 | 532.57 | 5.00 | 1.00 |
| 180.20 | 208.66 | 374.94 | 5.00 | 1.00 |
| 191.99 | 203.51 | 341.38 | 5.00 | 1.00 |
| 242.47 | 253.17 | 448.60 | 5.00 | 1.00 |
| 352.49 | 414.42 | 703.84 | 5.00 | 1.00 |
| 198.89 | 225.58 | 397.71 | 5.00 | 1.00 |
| 216.16 | 231.58 | 393.39 | 5.00 | 1.00 |
| 280.62 | 303.77 | 470.98 | 5.00 | 1.00 |
| 345.74 | 357.42 | 631.44 | 5.00 | 1.00 |
| 217.08 | 242.03 | 421.33 | 5.00 | 1.00 |
| 205.89 | 229.83 | 410.18 | 5.00 | 1.00 |
| 229.97 | 272.73 | 429.75 | 5.00 | 1.00 |
| 226.97 | 260.39 | 396.23 | 5.00 | 1.00 |
| 268.46 | 300.68 | 505.16 | 5.00 | 1.00 |
| 214.71 | 228.04 | 419.50 | 5.00 | 1.00 |

| | | | | |
|---|---|---|---|---|
| 236.26 | 240.00 | 435.19 | 5.00 | 1.00 |
| 220.00 | 251.82 | 429.78 | 5.00 | 1.00 |
| 226.71 | 275.14 | 398.93 | 5.00 | 1.00 |
| 256.40 | 274.61 | 480.60 | 5.00 | 1.00 |
| 340.71 | 360.43 | 600.79 | 5.00 | 1.00 |
| 179.88 | 158.73 | 297.37 | 5.00 | 1.00 |
| 256.80 | 304.80 | 505.20 | 5.00 | 1.00 |
| 202.50 | 238.64 | 358.64 | 5.00 | 1.00 |
| 215.79 | 258.90 | 395.27 | 5.00 | 1.00 |
| 287.86 | 305.97 | 489.42 | 5.00 | 1.00 |
| 187.79 | 181.47 | 313.15 | 5.00 | 1.00 |
| 273.39 | 289.47 | 511.44 | 5.00 | 1.00 |
| 216.67 | 210.31 | 372.12 | 5.00 | 1.00 |
| 204.92 | 216.68 | 361.90 | 5.00 | 1.00 |
| 219.03 | 267.31 | 400.79 | 5.00 | 1.00 |
| 269.85 | 305.26 | 468.07 | 5.00 | 1.00 |
| 163.16 | 135.52 | 256.08 | 5.00 | 1.00 |
| 224.14 | 247.84 | 422.36 | 5.00 | 1.00 |
| 269.86 | 278.59 | 474.93 | 5.00 | 1.00 |
| 208.84 | 240.20 | 373.02 | 5.00 | 1.00 |
| 220.47 | 247.93 | 374.71 | 5.00 | 1.00 |
| 166.59 | 161.99 | 293.37 | 5.00 | 1.00 |
| 206.86 | 237.78 | 391.54 | 5.00 | 1.00 |
| 296.24 | 333.52 | 552.63 | 5.00 | 1.00 |
| 208.15 | 223.96 | 378.95 | 5.00 | 1.00 |
| 209.71 | 230.60 | 362.71 | 5.00 | 1.00 |
| 169.92 | 185.72 | 322.26 | 5.00 | 1.00 |
| 185.61 | 205.68 | 349.71 | 5.00 | 1.00 |
| 272.73 | 293.42 | 510.03 | 5.00 | 1.00 |
| 213.92 | 243.12 | 396.47 | 5.00 | 1.00 |
| 178.63 | 198.91 | 337.79 | 5.00 | 1.00 |
| 174.93 | 185.45 | 328.31 | 5.00 | 1.00 |
| 212.31 | 230.59 | 385.48 | 5.00 | 1.00 |
| 342.97 | 409.69 | 691.51 | 5.00 | 1.00 |
| 167.49 | 202.67 | 332.84 | 5.00 | 1.00 |
| 225.84 | 225.85 | 421.55 | 5.00 | 1.00 |
| 202.49 | 221.22 | 381.62 | 5.00 | 1.00 |
| 327.84 | 352.01 | 621.62 | 5.00 | 1.00 |
| 182.25 | 206.33 | 382.20 | 5.00 | 1.00 |
| 235.32 | 272.26 | 480.73 | 5.00 | 1.00 |
| 234.61 | 252.95 | 440.51 | 5.00 | 1.00 |
| 296.91 | 370.27 | 633.82 | 5.00 | 1.00 |
| 184.80 | 220.15 | 380.16 | 5.00 | 1.00 |

| | | | | |
|---|---|---|---|---|
| 203.65 | 221.12 | 372.84 | 5.00 | 1.00 |
| 190.87 | 236.86 | 411.71 | 5.00 | 1.00 |
| 293.49 | 340.78 | 594.59 | 5.00 | 1.00 |
| 246.00 | 268.19 | 473.20 | 5.00 | 1.00 |
| 153.31 | 169.26 | 318.27 | 5.00 | 1.00 |
| 235.56 | 257.09 | 502.93 | 5.00 | 1.00 |
| 154.03 | 155.63 | 309.70 | 5.00 | 1.00 |
| 543.17 | 544.08 | 1001.02 | 5.00 | 1.00 |
| 262.06 | 322.88 | 649.53 | 5.00 | 1.00 |
| 323.59 | 338.85 | 688.83 | 5.00 | 1.00 |
| 292.69 | 298.37 | 612.60 | 5.00 | 1.00 |
| 210.57 | 230.15 | 469.00 | 5.00 | 1.00 |
| 290.59 | 326.60 | 586.27 | 5.00 | 1.00 |
| 232.02 | 229.90 | 505.40 | 5.00 | 1.00 |
| 223.85 | 322.69 | 662.46 | 5.00 | 1.00 |
| 234.05 | 256.40 | 522.76 | 5.00 | 1.00 |
| 347.73 | 369.45 | 688.28 | 5.00 | 1.00 |
| 318.54 | 335.14 | 641.72 | 5.00 | 1.00 |
| 254.50 | 320.00 | 643.93 | 5.00 | 1.00 |
| 300.47 | 332.46 | 642.10 | 5.00 | 1.00 |
| 243.78 | 266.26 | 499.40 | 5.00 | 1.00 |
| 190.02 | 290.37 | 572.79 | 5.00 | 1.00 |
| 297.67 | 334.64 | 657.26 | 5.00 | 1.00 |
| 280.81 | 256.68 | 547.44 | 5.00 | 1.00 |
| 237.77 | 352.46 | 699.85 | 5.00 | 1.00 |
| 285.86 | 368.96 | 690.88 | 5.00 | 1.00 |
| 316.45 | 381.64 | 732.99 | 5.00 | 1.00 |
| 240.78 | 283.39 | 570.23 | 5.00 | 1.00 |
| 182.09 | 267.01 | 517.43 | 5.00 | 1.00 |
| 265.93 | 332.89 | 600.06 | 5.00 | 1.00 |
| 263.42 | 293.65 | 515.16 | 5.00 | 1.00 |
| 167.71 | 216.55 | 408.89 | 5.00 | 1.00 |
| 191.75 | 220.43 | 466.51 | 5.00 | 1.00 |
| 237.54 | 277.98 | 534.41 | 5.00 | 1.00 |
| 264.46 | 332.31 | 604.27 | 5.00 | 1.00 |
| 202.89 | 283.15 | 495.26 | 5.00 | 1.00 |
| 350.02 | 431.27 | 799.95 | 5.00 | 1.00 |
| 241.20 | 255.25 | 520.60 | 5.00 | 1.00 |
| 237.22 | 337.35 | 558.46 | 5.00 | 1.00 |
| 237.53 | 259.10 | 488.27 | 5.00 | 1.00 |
| 180.00 | 203.97 | 380.43 | 5.00 | 1.00 |
| 384.46 | 467.54 | 889.14 | 5.00 | 1.00 |
| 294.75 | 296.70 | 574.91 | 5.00 | 1.00 |

| | | | | |
|---|---|---|---|---|
| 211.36 | 277.96 | 496.06 | 5.00 | 1.00 |
| 229.73 | 300.96 | 506.29 | 5.00 | 1.00 |
| 174.39 | 224.18 | 383.40 | 5.00 | 1.00 |
| 240.79 | 288.74 | 574.40 | 5.00 | 1.00 |
| 289.57 | 327.65 | 662.22 | 5.00 | 1.00 |
| 262.37 | 320.25 | 594.46 | 5.00 | 1.00 |
| 272.10 | 335.00 | 633.66 | 5.00 | 1.00 |
| 179.27 | 244.99 | 437.66 | 5.00 | 1.00 |
| 194.24 | 248.03 | 394.65 | 5.00 | 1.00 |
| 234.76 | 239.57 | 502.56 | 5.00 | 1.00 |
| 231.98 | 278.38 | 533.30 | 5.00 | 1.00 |
| 167.58 | 218.02 | 405.06 | 5.00 | 1.00 |
| 353.60 | 456.90 | 857.38 | 5.00 | 1.00 |
| 290.88 | 375.96 | 605.80 | 5.00 | 1.00 |
| 245.96 | 341.86 | 553.19 | 5.00 | 1.00 |
| 254.43 | 286.81 | 574.24 | 5.00 | 1.00 |
| 191.11 | 211.42 | 398.05 | 5.00 | 2.00 |
| 225.34 | 244.88 | 470.97 | 5.00 | 2.00 |
| 175.62 | 172.42 | 334.71 | 5.00 | 2.00 |
| 274.33 | 288.39 | 498.82 | 5.00 | 2.00 |
| 162.86 | 173.95 | 306.43 | 5.00 | 2.00 |
| 223.56 | 239.27 | 439.23 | 5.00 | 2.00 |
| 192.34 | 211.51 | 398.09 | 5.00 | 2.00 |
| 167.35 | 168.77 | 352.92 | 5.00 | 2.00 |
| 188.24 | 191.02 | 352.92 | 5.00 | 2.00 |
| 207.67 | 222.21 | 441.57 | 5.00 | 2.00 |
| 203.29 | 210.83 | 439.59 | 5.00 | 2.00 |
| 209.59 | 199.75 | 385.21 | 5.00 | 2.00 |
| 230.10 | 216.07 | 471.23 | 5.00 | 2.00 |
| 200.01 | 207.94 | 418.32 | 5.00 | 2.00 |
| 162.82 | 179.47 | 354.56 | 5.00 | 2.00 |
| 227.25 | 195.19 | 415.88 | 5.00 | 2.00 |
| 213.81 | 220.74 | 444.12 | 5.00 | 2.00 |
| 174.05 | 178.33 | 335.94 | 5.00 | 2.00 |
| 202.72 | 201.81 | 422.58 | 5.00 | 2.00 |
| 299.23 | 262.61 | 579.79 | 5.00 | 2.00 |
| 245.51 | 242.19 | 466.35 | 5.00 | 2.00 |
| 247.81 | 233.78 | 487.27 | 5.00 | 2.00 |
| 190.54 | 203.03 | 409.69 | 5.00 | 2.00 |
| 274.89 | 239.06 | 522.32 | 5.00 | 2.00 |
| 255.98 | 248.24 | 445.36 | 5.00 | 2.00 |
| 280.87 | 268.14 | 541.45 | 5.00 | 2.00 |
| 256.11 | 234.07 | 503.74 | 5.00 | 2.00 |

| | | | | |
|---|---|---|---|---|
| 181.34 | 212.08 | 430.25 | 5.00 | 2.00 |
| 218.86 | 239.56 | 437.92 | 5.00 | 2.00 |
| 292.39 | 275.62 | 522.65 | 5.00 | 2.00 |
| 202.50 | 207.03 | 379.44 | 5.00 | 2.00 |
| 210.77 | 201.04 | 406.29 | 5.00 | 2.00 |
| 193.77 | 193.57 | 423.63 | 5.00 | 2.00 |
| 288.46 | 295.11 | 586.70 | 5.00 | 2.00 |
| 257.30 | 256.55 | 527.30 | 5.00 | 2.00 |
| 254.00 | 229.87 | 453.16 | 5.00 | 2.00 |
| 147.39 | 144.32 | 317.21 | 5.00 | 2.00 |
| 195.30 | 171.01 | 380.88 | 5.00 | 2.00 |
| 273.81 | 301.27 | 590.58 | 5.00 | 2.00 |
| 199.84 | 196.63 | 410.84 | 5.00 | 2.00 |
| 235.81 | 225.56 | 505.64 | 5.00 | 2.00 |
| 164.31 | 146.84 | 317.73 | 5.00 | 2.00 |
| 175.47 | 147.63 | 320.12 | 5.00 | 2.00 |
| 318.62 | 288.26 | 593.21 | 5.00 | 2.00 |
| 209.40 | 220.80 | 446.46 | 5.00 | 2.00 |
| 294.70 | 284.32 | 605.42 | 5.00 | 2.00 |
| 234.33 | 200.83 | 417.26 | 5.00 | 2.00 |
| 173.40 | 161.68 | 339.97 | 5.00 | 2.00 |
| 262.19 | 206.31 | 464.20 | 5.00 | 2.00 |
| 199.47 | 221.57 | 438.59 | 5.00 | 2.00 |
| 295.62 | 304.05 | 595.85 | 5.00 | 2.00 |
| 278.34 | 233.51 | 501.57 | 5.00 | 2.00 |
| 212.59 | 197.24 | 434.39 | 5.00 | 2.00 |
| 218.31 | 186.87 | 384.37 | 5.00 | 2.00 |
| 198.04 | 195.42 | 439.29 | 5.00 | 2.00 |
| 233.38 | 256.36 | 471.22 | 5.00 | 2.00 |
| 591.14 | 574.54 | 1134.36 | 5.00 | 2.00 |
| 220.86 | 226.43 | 475.72 | 5.00 | 2.00 |
| 291.82 | 240.28 | 509.76 | 5.00 | 2.00 |
| 178.11 | 193.58 | 388.81 | 5.00 | 2.00 |
| 287.59 | 288.36 | 552.74 | 5.00 | 2.00 |
| 216.97 | 224.59 | 450.68 | 5.00 | 2.00 |
| 287.95 | 355.17 | 682.64 | 5.00 | 2.00 |
| 265.81 | 216.93 | 455.47 | 5.00 | 2.00 |
| 251.90 | 279.20 | 520.38 | 5.00 | 2.00 |
| 202.71 | 191.76 | 375.32 | 5.00 | 2.00 |
| 164.03 | 189.92 | 377.10 | 5.00 | 2.00 |
| 215.13 | 216.80 | 412.12 | 5.00 | 2.00 |
| 231.75 | 247.56 | 485.17 | 5.00 | 2.00 |
| 239.86 | 231.75 | 493.29 | 5.00 | 2.00 |

| | | | | |
|---|---|---|---|---|
| 219.70 | 228.04 | 434.39 | 5.00 | 2.00 |
| 167.74 | 186.29 | 362.00 | 5.00 | 2.00 |
| 158.23 | 207.19 | 409.25 | 5.00 | 2.00 |
| 192.50 | 180.65 | 417.09 | 5.00 | 2.00 |
| 194.20 | 222.37 | 467.58 | 5.00 | 2.00 |
| 130.80 | 164.14 | 324.72 | 5.00 | 2.00 |
| 180.87 | 241.59 | 502.81 | 5.00 | 2.00 |
| 136.89 | 153.32 | 297.08 | 5.00 | 2.00 |
| 141.34 | 206.09 | 405.01 | 5.00 | 2.00 |
| 146.79 | 187.76 | 346.81 | 5.00 | 2.00 |
| 165.46 | 220.39 | 423.87 | 5.00 | 2.00 |
| 138.76 | 183.69 | 345.79 | 5.00 | 2.00 |
| 189.27 | 208.68 | 400.63 | 5.00 | 2.00 |
| 162.31 | 225.01 | 397.63 | 5.00 | 2.00 |
| 169.86 | 254.16 | 501.68 | 5.00 | 2.00 |
| 145.24 | 172.75 | 331.04 | 5.00 | 2.00 |
| 138.45 | 161.13 | 325.72 | 5.00 | 2.00 |
| 209.19 | 274.46 | 554.35 | 5.00 | 2.00 |
| 140.83 | 177.44 | 330.06 | 5.00 | 2.00 |
| 127.38 | 159.32 | 301.49 | 5.00 | 2.00 |
| 142.17 | 197.00 | 345.53 | 5.00 | 2.00 |
| 139.80 | 201.52 | 375.19 | 5.00 | 2.00 |
| 122.63 | 169.41 | 314.58 | 5.00 | 2.00 |
| 156.18 | 201.42 | 386.46 | 5.00 | 2.00 |
| 204.47 | 253.83 | 464.68 | 5.00 | 2.00 |
| 158.17 | 240.69 | 437.97 | 5.00 | 2.00 |
| 222.54 | 260.05 | 542.26 | 5.00 | 2.00 |
| 181.46 | 209.64 | 437.89 | 5.00 | 2.00 |
| 178.45 | 248.16 | 428.32 | 5.00 | 2.00 |
| 205.53 | 248.11 | 455.31 | 5.00 | 2.00 |
| 139.97 | 145.99 | 294.45 | 5.00 | 2.00 |
| 182.38 | 183.23 | 391.86 | 5.00 | 2.00 |
| 141.50 | 204.01 | 356.34 | 5.00 | 2.00 |
| 183.36 | 278.41 | 546.29 | 5.00 | 2.00 |
| 184.63 | 229.66 | 445.30 | 5.00 | 2.00 |
| 140.86 | 163.76 | 338.00 | 5.00 | 2.00 |
| 191.43 | 211.04 | 374.88 | 5.00 | 2.00 |
| 167.81 | 206.75 | 427.00 | 5.00 | 2.00 |
| 381.04 | 459.91 | 929.22 | 5.00 | 2.00 |
| 180.81 | 273.94 | 495.19 | 5.00 | 2.00 |
| 171.64 | 221.98 | 417.92 | 5.00 | 2.00 |
| 182.37 | 210.70 | 375.88 | 5.00 | 2.00 |
| 197.94 | 217.41 | 438.84 | 5.00 | 2.00 |

| | | | | |
|---|---|---|---|---|
| 302.93 | 425.20 | 744.59 | 5.00 | 2.00 |
| 200.71 | 220.09 | 442.41 | 5.00 | 2.00 |
| 133.31 | 180.57 | 364.23 | 5.00 | 2.00 |
| 187.99 | 213.08 | 412.22 | 5.00 | 2.00 |
| 192.05 | 195.49 | 417.93 | 5.00 | 2.00 |
| 263.72 | 325.19 | 578.16 | 5.00 | 2.00 |
| 321.11 | 384.43 | 820.70 | 5.00 | 2.00 |
| 222.73 | 308.22 | 560.48 | 5.00 | 2.00 |
| 177.89 | 245.97 | 486.57 | 5.00 | 2.00 |
| 150.04 | 188.29 | 320.12 | 5.00 | 2.00 |
| 287.93 | 300.15 | 617.56 | 5.00 | 2.00 |
| 207.59 | 222.54 | 435.13 | 5.00 | 2.00 |
| 248.52 | 319.94 | 607.09 | 5.00 | 2.00 |
| 240.77 | 242.14 | 461.09 | 5.00 | 2.00 |
| 159.18 | 185.52 | 398.84 | 5.00 | 2.00 |
| 178.07 | 188.14 | 358.79 | 5.00 | 2.00 |
| 240.56 | 303.60 | 556.99 | 5.00 | 2.00 |
| 296.54 | 277.96 | 620.30 | 5.00 | 2.00 |
| 200.36 | 255.46 | 484.94 | 5.00 | 2.00 |
| 211.20 | 222.17 | 437.54 | 5.00 | 2.00 |
| 151.97 | 191.02 | 398.99 | 5.00 | 2.00 |
| 255.80 | 272.36 | 525.25 | 5.00 | 2.00 |
| 167.34 | 210.10 | 420.36 | 5.00 | 2.00 |
| 150.35 | 187.95 | 384.22 | 5.00 | 2.00 |
| 211.07 | 223.06 | 437.79 | 5.00 | 2.00 |
| 176.81 | 217.75 | 425.40 | 5.00 | 2.00 |
| 207.45 | 223.78 | 442.31 | 5.00 | 2.00 |
| 137.77 | 183.84 | 328.00 | 5.00 | 2.00 |
| 198.24 | 200.78 | 387.86 | 5.00 | 3.00 |
| 217.49 | 234.27 | 397.10 | 5.00 | 3.00 |
| 185.92 | 173.15 | 348.52 | 5.00 | 3.00 |
| 182.92 | 190.65 | 415.08 | 5.00 | 3.00 |
| 201.31 | 185.37 | 347.15 | 5.00 | 3.00 |
| 168.52 | 172.91 | 357.38 | 5.00 | 3.00 |
| 190.79 | 171.73 | 336.06 | 5.00 | 3.00 |
| 120.56 | 138.87 | 306.47 | 5.00 | 3.00 |
| 155.41 | 145.84 | 307.23 | 5.00 | 3.00 |
| 170.83 | 157.86 | 322.27 | 5.00 | 3.00 |
| 154.82 | 187.66 | 378.69 | 5.00 | 3.00 |
| 160.42 | 166.04 | 330.22 | 5.00 | 3.00 |
| 156.15 | 160.58 | 306.08 | 5.00 | 3.00 |
| 174.98 | 195.92 | 399.87 | 5.00 | 3.00 |
| 187.20 | 173.40 | 354.92 | 5.00 | 3.00 |

| | | | | |
|---|---|---|---|---|
| 142.62 | 166.92 | 321.11 | 5.00 | 3.00 |
| 164.83 | 176.84 | 332.36 | 5.00 | 3.00 |
| 161.25 | 181.95 | 363.00 | 5.00 | 3.00 |
| 188.40 | 179.21 | 358.89 | 5.00 | 3.00 |
| 157.24 | 173.25 | 365.68 | 5.00 | 3.00 |
| 165.09 | 169.45 | 322.53 | 5.00 | 3.00 |
| 180.55 | 181.47 | 371.62 | 5.00 | 3.00 |
| 231.26 | 216.08 | 398.69 | 5.00 | 3.00 |
| 213.30 | 194.11 | 416.94 | 5.00 | 3.00 |
| 141.21 | 172.02 | 319.93 | 5.00 | 3.00 |
| 178.72 | 177.04 | 362.88 | 5.00 | 3.00 |
| 206.61 | 210.55 | 401.47 | 5.00 | 3.00 |
| 204.30 | 209.12 | 399.44 | 5.00 | 3.00 |
| 222.51 | 211.69 | 452.85 | 5.00 | 3.00 |
| 143.78 | 171.06 | 310.55 | 5.00 | 3.00 |
| 215.65 | 223.38 | 447.02 | 5.00 | 3.00 |
| 211.50 | 203.96 | 411.64 | 5.00 | 3.00 |
| 257.23 | 274.19 | 528.15 | 5.00 | 3.00 |
| 226.82 | 219.93 | 452.74 | 5.00 | 3.00 |
| 200.09 | 215.94 | 410.93 | 5.00 | 3.00 |
| 255.73 | 230.65 | 452.94 | 5.00 | 3.00 |
| 183.30 | 177.06 | 378.20 | 5.00 | 3.00 |
| 225.25 | 227.77 | 433.44 | 5.00 | 3.00 |
| 205.59 | 222.14 | 489.15 | 5.00 | 3.00 |
| 207.93 | 230.66 | 423.56 | 5.00 | 3.00 |
| 204.11 | 206.41 | 413.71 | 5.00 | 3.00 |
| 288.77 | 226.56 | 511.10 | 5.00 | 3.00 |
| 179.78 | 171.65 | 353.93 | 5.00 | 3.00 |
| 236.79 | 237.63 | 459.65 | 5.00 | 3.00 |
| 192.10 | 209.80 | 433.95 | 5.00 | 3.00 |
| 212.45 | 253.37 | 457.36 | 5.00 | 3.00 |
| 207.92 | 196.52 | 399.29 | 5.00 | 3.00 |
| 217.28 | 185.39 | 438.62 | 5.00 | 3.00 |
| 246.43 | 243.24 | 475.96 | 5.00 | 3.00 |
| 209.72 | 220.12 | 430.84 | 5.00 | 3.00 |
| 146.07 | 188.54 | 343.31 | 5.00 | 3.00 |
| 132.35 | 182.22 | 337.91 | 5.00 | 3.00 |
| 161.36 | 182.25 | 383.71 | 5.00 | 3.00 |
| 262.29 | 224.56 | 515.48 | 5.00 | 3.00 |
| 186.93 | 197.07 | 372.40 | 5.00 | 3.00 |
| 221.21 | 191.65 | 452.85 | 5.00 | 3.00 |
| 191.03 | 201.03 | 397.80 | 5.00 | 3.00 |
| 125.20 | 165.96 | 314.51 | 5.00 | 3.00 |

| | | | | |
|---|---|---|---|---|
| 172.05 | 193.17 | 387.35 | 5.00 | 3.00 |
| 263.97 | 228.72 | 522.43 | 5.00 | 3.00 |
| 166.76 | 176.67 | 368.75 | 5.00 | 3.00 |
| 228.12 | 199.29 | 427.81 | 5.00 | 3.00 |
| 214.66 | 206.99 | 412.84 | 5.00 | 3.00 |
| 156.48 | 197.03 | 365.24 | 5.00 | 3.00 |
| 162.81 | 166.05 | 350.76 | 5.00 | 3.00 |
| 225.83 | 220.89 | 453.95 | 5.00 | 3.00 |
| 154.91 | 159.43 | 350.11 | 5.00 | 3.00 |
| 193.69 | 178.69 | 358.10 | 5.00 | 3.00 |
| 164.30 | 178.67 | 343.44 | 5.00 | 3.00 |
| 189.60 | 196.70 | 409.02 | 5.00 | 3.00 |
| 235.25 | 261.54 | 514.19 | 5.00 | 3.00 |
| 162.14 | 176.00 | 349.60 | 5.00 | 3.00 |
| 197.11 | 208.50 | 416.37 | 5.00 | 3.00 |
| 212.45 | 188.18 | 364.02 | 5.00 | 3.00 |
| 190.48 | 197.85 | 395.78 | 5.00 | 3.00 |
| 259.34 | 257.60 | 529.87 | 5.00 | 3.00 |
| 198.39 | 217.22 | 413.42 | 5.00 | 3.00 |
| 189.82 | 183.03 | 373.36 | 5.00 | 3.00 |
| 176.94 | 167.34 | 334.06 | 5.00 | 3.00 |
| 196.67 | 218.21 | 416.11 | 5.00 | 3.00 |
| 185.96 | 213.98 | 408.99 | 5.00 | 3.00 |
| 157.40 | 172.59 | 343.58 | 5.00 | 3.00 |
| 190.65 | 208.61 | 363.68 | 5.00 | 3.00 |
| 161.42 | 134.93 | 316.84 | 5.00 | 3.00 |
| 152.39 | 167.97 | 331.70 | 5.00 | 3.00 |
| 162.04 | 208.20 | 394.62 | 5.00 | 3.00 |
| 143.43 | 144.57 | 326.19 | 5.00 | 3.00 |
| 176.40 | 177.14 | 345.09 | 5.00 | 3.00 |
| 188.38 | 174.50 | 359.87 | 5.00 | 3.00 |
| 176.99 | 165.33 | 357.95 | 5.00 | 3.00 |
| 142.25 | 232.68 | 367.96 | 5.00 | 3.00 |
| 159.99 | 223.43 | 409.21 | 5.00 | 3.00 |
| 168.84 | 227.59 | 414.38 | 5.00 | 3.00 |
| 150.65 | 220.47 | 362.00 | 5.00 | 3.00 |
| 158.75 | 197.49 | 375.38 | 5.00 | 3.00 |
| 172.03 | 257.33 | 459.49 | 5.00 | 3.00 |
| 168.21 | 240.31 | 427.35 | 5.00 | 3.00 |
| 160.55 | 235.60 | 423.89 | 5.00 | 3.00 |
| 218.44 | 285.87 | 530.09 | 5.00 | 3.00 |
| 166.20 | 260.43 | 448.47 | 5.00 | 3.00 |
| 182.84 | 255.64 | 420.47 | 5.00 | 3.00 |

| | | | | |
|---|---|---|---|---|
| 182.36 | 242.74 | 429.25 | 5.00 | 3.00 |
| 186.81 | 234.64 | 436.74 | 5.00 | 3.00 |
| 144.00 | 222.53 | 368.81 | 5.00 | 3.00 |
| 182.72 | 223.68 | 385.30 | 5.00 | 3.00 |
| 207.80 | 227.95 | 449.89 | 5.00 | 3.00 |
| 151.59 | 209.23 | 353.89 | 5.00 | 3.00 |
| 181.14 | 249.23 | 401.63 | 5.00 | 3.00 |
| 209.64 | 262.83 | 518.95 | 5.00 | 3.00 |
| 211.88 | 267.91 | 458.68 | 5.00 | 3.00 |
| 173.03 | 204.05 | 358.63 | 5.00 | 3.00 |
| 244.65 | 302.90 | 513.68 | 5.00 | 3.00 |
| 210.59 | 278.58 | 518.19 | 5.00 | 3.00 |
| 206.17 | 271.17 | 444.34 | 5.00 | 3.00 |
| 126.70 | 173.58 | 304.19 | 5.00 | 3.00 |
| 242.92 | 282.44 | 502.23 | 5.00 | 3.00 |
| 186.48 | 241.19 | 408.62 | 5.00 | 3.00 |
| 204.76 | 223.00 | 423.08 | 5.00 | 3.00 |
| 159.35 | 222.29 | 357.39 | 5.00 | 3.00 |
| 133.29 | 162.94 | 292.99 | 5.00 | 3.00 |
| 147.26 | 205.41 | 353.30 | 5.00 | 3.00 |
| 255.68 | 326.52 | 547.91 | 5.00 | 3.00 |
| 165.42 | 180.13 | 330.75 | 5.00 | 3.00 |
| 129.43 | 165.61 | 292.60 | 5.00 | 3.00 |
| 141.98 | 182.31 | 335.51 | 5.00 | 3.00 |
| 145.84 | 209.44 | 381.04 | 5.00 | 3.00 |
| 196.31 | 266.77 | 457.82 | 5.00 | 3.00 |
| 158.72 | 183.28 | 309.32 | 5.00 | 3.00 |
| 129.79 | 164.00 | 285.35 | 5.00 | 3.00 |
| 134.43 | 223.51 | 356.84 | 5.00 | 3.00 |
| 146.99 | 200.26 | 364.01 | 5.00 | 3.00 |
| 144.32 | 189.80 | 356.42 | 5.00 | 3.00 |
| 117.37 | 167.14 | 291.01 | 5.00 | 3.00 |
| 161.50 | 189.46 | 325.53 | 5.00 | 3.00 |
| 177.18 | 235.29 | 394.02 | 5.00 | 3.00 |
| 113.18 | 187.34 | 303.56 | 5.00 | 3.00 |
| 147.25 | 195.49 | 347.15 | 5.00 | 3.00 |
| 145.58 | 197.09 | 348.38 | 5.00 | 3.00 |
| 125.52 | 164.00 | 297.73 | 5.00 | 3.00 |
| 140.50 | 177.79 | 306.67 | 5.00 | 3.00 |
| 164.33 | 235.89 | 410.73 | 5.00 | 3.00 |
| 117.12 | 172.70 | 313.87 | 5.00 | 3.00 |
| 149.32 | 187.68 | 349.99 | 5.00 | 3.00 |
| 161.63 | 231.78 | 397.35 | 5.00 | 3.00 |

| | | | | |
|---|---|---|---|---|
| 135.99 | 169.51 | 296.14 | 5.00 | 3.00 |
| 141.31 | 179.46 | 328.99 | 5.00 | 3.00 |
| 130.66 | 182.31 | 340.75 | 5.00 | 3.00 |
| 131.59 | 178.76 | 324.79 | 5.00 | 3.00 |
| 136.32 | 193.56 | 321.25 | 5.00 | 3.00 |
| 154.16 | 213.15 | 373.08 | 5.00 | 3.00 |
| 129.22 | 166.72 | 306.25 | 5.00 | 3.00 |
| 149.56 | 181.76 | 326.09 | 5.00 | 3.00 |
| 179.07 | 208.51 | 384.32 | 5.00 | 3.00 |
| 163.06 | 221.11 | 377.84 | 5.00 | 3.00 |
| 131.15 | 167.79 | 292.77 | 5.00 | 3.00 |
| 204.48 | 244.61 | 439.81 | 5.00 | 3.00 |
| 182.18 | 214.08 | 381.04 | 5.00 | 3.00 |
| 176.31 | 224.59 | 372.08 | 5.00 | 3.00 |
| 220.81 | 298.92 | 521.09 | 5.00 | 3.00 |
| 188.29 | 213.95 | 391.56 | 5.00 | 3.00 |
| 166.10 | 205.97 | 370.90 | 5.00 | 3.00 |
| 254.46 | 297.88 | 510.94 | 5.00 | 3.00 |
| 171.69 | 225.07 | 387.00 | 5.00 | 3.00 |
| 198.59 | 275.96 | 501.07 | 5.00 | 3.00 |
| 210.59 | 237.35 | 422.32 | 5.00 | 3.00 |
| 182.01 | 261.65 | 470.12 | 5.00 | 3.00 |
| 148.59 | 212.21 | 377.75 | 5.00 | 3.00 |
| 155.93 | 223.89 | 408.80 | 5.00 | 3.00 |
| 226.71 | 221.40 | 238.80 | 5.00 | 4.00 |
| 224.03 | 211.15 | 215.01 | 5.00 | 4.00 |
| 218.21 | 212.03 | 227.05 | 5.00 | 4.00 |
| 220.28 | 197.29 | 264.94 | 5.00 | 4.00 |
| 191.43 | 212.70 | 266.07 | 5.00 | 4.00 |
| 231.05 | 195.64 | 273.33 | 5.00 | 4.00 |
| 201.76 | 212.49 | 243.98 | 5.00 | 4.00 |
| 246.99 | 209.71 | 267.45 | 5.00 | 4.00 |
| 235.11 | 218.90 | 236.98 | 5.00 | 4.00 |
| 203.95 | 208.28 | 263.78 | 5.00 | 4.00 |
| 245.04 | 199.45 | 247.21 | 5.00 | 4.00 |
| 220.41 | 227.22 | 248.54 | 5.00 | 4.00 |
| 193.54 | 202.77 | 262.04 | 5.00 | 4.00 |
| 223.32 | 203.82 | 248.25 | 5.00 | 4.00 |
| 219.00 | 242.60 | 247.59 | 5.00 | 4.00 |
| 211.35 | 228.44 | 241.27 | 5.00 | 4.00 |
| 198.47 | 217.19 | 265.36 | 5.00 | 4.00 |
| 219.59 | 192.75 | 224.12 | 5.00 | 4.00 |
| 210.87 | 246.09 | 222.58 | 5.00 | 4.00 |

| | | | | |
|---|---|---|---|---|
| 226.52 | 236.52 | 243.63 | 5.00 | 4.00 |
| 207.37 | 230.37 | 239.95 | 5.00 | 4.00 |
| 215.30 | 194.78 | 226.83 | 5.00 | 4.00 |
| 223.53 | 252.58 | 238.75 | 5.00 | 4.00 |
| 239.17 | 237.96 | 223.51 | 5.00 | 4.00 |
| 220.97 | 224.75 | 250.96 | 5.00 | 4.00 |
| 201.06 | 204.03 | 212.53 | 5.00 | 4.00 |
| 229.03 | 259.71 | 313.41 | 5.00 | 4.00 |
| 217.15 | 250.09 | 218.73 | 5.00 | 4.00 |
| 241.04 | 231.54 | 246.89 | 5.00 | 4.00 |
| 209.87 | 211.80 | 210.32 | 5.00 | 4.00 |
| 204.58 | 199.21 | 258.12 | 5.00 | 4.00 |
| 222.71 | 252.01 | 237.42 | 5.00 | 4.00 |
| 208.07 | 256.49 | 223.45 | 5.00 | 4.00 |
| 227.66 | 221.21 | 240.72 | 5.00 | 4.00 |
| 227.12 | 208.59 | 208.75 | 5.00 | 4.00 |
| 187.37 | 194.88 | 273.33 | 5.00 | 4.00 |
| 212.06 | 228.18 | 235.31 | 5.00 | 4.00 |
| 201.35 | 253.46 | 226.69 | 5.00 | 4.00 |
| 223.95 | 227.79 | 245.25 | 5.00 | 4.00 |
| 212.82 | 210.22 | 235.15 | 5.00 | 4.00 |
| 199.38 | 210.93 | 272.34 | 5.00 | 4.00 |
| 199.50 | 214.77 | 242.03 | 5.00 | 4.00 |
| 209.40 | 222.94 | 228.57 | 5.00 | 4.00 |
| 206.48 | 241.63 | 267.05 | 5.00 | 4.00 |
| 235.69 | 222.67 | 259.14 | 5.00 | 4.00 |
| 222.91 | 209.30 | 258.32 | 5.00 | 4.00 |
| 208.07 | 214.92 | 231.46 | 5.00 | 4.00 |
| 210.81 | 229.88 | 241.99 | 5.00 | 4.00 |
| 205.34 | 242.01 | 264.51 | 5.00 | 4.00 |
| 208.17 | 232.95 | 271.04 | 5.00 | 4.00 |
| 234.13 | 232.88 | 220.31 | 5.00 | 4.00 |
| 242.56 | 224.93 | 235.07 | 5.00 | 4.00 |
| 230.19 | 231.63 | 274.93 | 5.00 | 4.00 |
| 202.21 | 232.49 | 280.85 | 5.00 | 4.00 |
| 224.99 | 219.63 | 231.91 | 5.00 | 4.00 |
| 190.06 | 237.70 | 275.08 | 5.00 | 4.00 |
| 203.90 | 248.32 | 257.63 | 5.00 | 4.00 |
| 244.26 | 237.85 | 257.95 | 5.00 | 4.00 |
| 236.37 | 222.22 | 227.22 | 5.00 | 4.00 |
| 216.76 | 236.34 | 249.75 | 5.00 | 4.00 |
| 223.25 | 236.55 | 252.77 | 5.00 | 4.00 |
| 231.87 | 228.20 | 290.40 | 5.00 | 4.00 |

| | | | | |
|---|---|---|---|---|
| 207.43 | 264.23 | 273.87 | 5.00 | 4.00 |
| 209.14 | 244.03 | 243.51 | 5.00 | 4.00 |
| 250.28 | 229.82 | 268.25 | 5.00 | 4.00 |
| 254.00 | 247.72 | 260.14 | 5.00 | 4.00 |
| 198.27 | 237.50 | 261.43 | 5.00 | 4.00 |
| 237.48 | 232.57 | 316.05 | 5.00 | 4.00 |
| 219.80 | 237.95 | 262.14 | 5.00 | 4.00 |
| 197.08 | 247.74 | 265.05 | 5.00 | 4.00 |
| 250.03 | 243.31 | 283.10 | 5.00 | 4.00 |
| 249.16 | 237.57 | 226.18 | 5.00 | 4.00 |
| 241.58 | 246.50 | 256.86 | 5.00 | 4.00 |
| 219.80 | 253.48 | 257.98 | 5.00 | 4.00 |
| 247.42 | 230.33 | 239.48 | 5.00 | 4.00 |
| 223.30 | 250.34 | 250.07 | 5.00 | 4.00 |
| 235.55 | 237.99 | 480.04 | 5.00 | 4.00 |
| 223.70 | 249.30 | 485.57 | 5.00 | 4.00 |
| 220.37 | 292.50 | 452.91 | 5.00 | 4.00 |
| 229.00 | 243.68 | 395.27 | 5.00 | 4.00 |
| 207.40 | 272.88 | 419.14 | 5.00 | 4.00 |
| 210.28 | 253.22 | 297.01 | 5.00 | 4.00 |
| 236.41 | 267.11 | 364.13 | 5.00 | 4.00 |
| 205.49 | 246.61 | 424.13 | 5.00 | 4.00 |
| 202.22 | 251.02 | 303.84 | 5.00 | 4.00 |
| 215.12 | 263.85 | 347.22 | 5.00 | 4.00 |
| 223.53 | 248.47 | 376.74 | 5.00 | 4.00 |
| 252.84 | 242.65 | 336.22 | 5.00 | 4.00 |
| 202.35 | 244.62 | 276.88 | 5.00 | 4.00 |
| 227.90 | 245.54 | 314.86 | 5.00 | 4.00 |
| 260.19 | 226.29 | 288.65 | 5.00 | 4.00 |
| 212.37 | 249.77 | 265.77 | 5.00 | 4.00 |
| 210.85 | 247.55 | 294.58 | 5.00 | 4.00 |
| 242.65 | 230.92 | 310.18 | 5.00 | 4.00 |
| 203.45 | 252.68 | 337.48 | 5.00 | 4.00 |
| 205.51 | 225.52 | 278.51 | 5.00 | 4.00 |
| 229.34 | 266.55 | 311.41 | 5.00 | 4.00 |
| 239.13 | 233.49 | 279.20 | 5.00 | 4.00 |
| 199.46 | 232.63 | 288.94 | 5.00 | 4.00 |
| 214.76 | 256.70 | 398.23 | 5.00 | 4.00 |
| 220.42 | 252.67 | 285.05 | 5.00 | 4.00 |
| 247.90 | 246.94 | 381.35 | 5.00 | 4.00 |
| 199.62 | 256.76 | 364.27 | 5.00 | 4.00 |
| 234.38 | 199.93 | 281.85 | 5.00 | 4.00 |
| 187.19 | 268.93 | 282.89 | 5.00 | 4.00 |

| | | | | |
|---|---|---|---|---|
| 232.75 | 288.75 | 325.92 | 5.00 | 4.00 |
| 263.99 | 254.07 | 286.94 | 5.00 | 4.00 |
| 209.19 | 238.70 | 319.14 | 5.00 | 4.00 |
| 208.08 | 253.59 | 380.06 | 5.00 | 4.00 |
| 201.07 | 210.92 | 308.50 | 5.00 | 4.00 |
| 241.14 | 278.21 | 275.29 | 5.00 | 4.00 |
| 212.28 | 246.94 | 330.68 | 5.00 | 4.00 |
| 231.09 | 238.29 | 339.18 | 5.00 | 4.00 |
| 214.59 | 248.61 | 333.87 | 5.00 | 4.00 |
| 207.43 | 266.30 | 323.45 | 5.00 | 4.00 |
| 224.91 | 220.14 | 282.81 | 5.00 | 4.00 |
| 203.36 | 273.40 | 309.62 | 5.00 | 4.00 |
| 206.04 | 255.49 | 321.76 | 5.00 | 4.00 |
| 236.47 | 216.44 | 381.58 | 5.00 | 4.00 |
| 194.12 | 233.37 | 319.87 | 5.00 | 4.00 |
| 214.66 | 262.20 | 282.43 | 5.00 | 4.00 |
| 209.88 | 227.00 | 283.71 | 5.00 | 4.00 |
| 202.50 | 264.03 | 327.98 | 5.00 | 4.00 |
| 212.33 | 279.81 | 334.88 | 5.00 | 4.00 |
| 212.71 | 198.00 | 346.30 | 5.00 | 4.00 |
| 170.66 | 233.10 | 311.62 | 5.00 | 4.00 |
| 206.99 | 252.05 | 341.20 | 5.00 | 4.00 |
| 216.09 | 265.09 | 314.79 | 5.00 | 4.00 |
| 208.31 | 247.45 | 323.52 | 5.00 | 4.00 |
| 202.29 | 292.86 | 370.07 | 5.00 | 4.00 |
| 183.85 | 262.59 | 386.22 | 5.00 | 4.00 |
| 195.06 | 233.93 | 304.24 | 5.00 | 4.00 |
| 225.58 | 253.99 | 358.14 | 5.00 | 4.00 |
| 229.09 | 265.20 | 314.70 | 5.00 | 4.00 |
| 204.59 | 242.29 | 312.47 | 5.00 | 4.00 |
| 187.73 | 287.74 | 385.90 | 5.00 | 4.00 |
| 209.14 | 265.37 | 434.41 | 5.00 | 4.00 |
| 217.27 | 233.81 | 309.49 | 5.00 | 4.00 |
| 255.36 | 245.86 | 367.27 | 5.00 | 4.00 |
| 233.37 | 253.53 | 327.45 | 5.00 | 4.00 |
| 194.09 | 257.32 | 268.78 | 5.00 | 4.00 |
| 198.39 | 280.45 | 322.02 | 5.00 | 4.00 |
| 246.64 | 244.66 | 372.75 | 5.00 | 4.00 |
| 235.58 | 233.66 | 324.19 | 5.00 | 4.00 |
| 189.56 | 255.18 | 416.84 | 5.00 | 4.00 |
| 198.99 | 250.96 | 290.14 | 5.00 | 4.00 |
| 219.77 | 255.17 | 285.31 | 5.00 | 4.00 |
| 206.46 | 249.15 | 297.04 | 5.00 | 4.00 |

| | | | | |
|---|---|---|---|---|
| 213.41 | 221.42 | 328.55 | 5.00 | 4.00 |
| 218.02 | 249.84 | 297.19 | 5.00 | 4.00 |
| 211.14 | 248.23 | 363.88 | 5.00 | 4.00 |
| 232.38 | 257.50 | 224.05 | 5.00 | 4.00 |
| 237.06 | 262.36 | 299.69 | 5.00 | 4.00 |
| 216.76 | 220.68 | 328.35 | 5.00 | 4.00 |
| 222.69 | 251.23 | 294.51 | 5.00 | 4.00 |
| 231.23 | 257.29 | 322.84 | 5.00 | 4.00 |
| 190.64 | 247.43 | 233.23 | 5.00 | 4.00 |
| 232.69 | 264.68 | 327.85 | 5.00 | 4.00 |
| 193.14 | 216.30 | 233.74 | 5.00 | 4.00 |
| 225.66 | 251.65 | 422.38 | 5.00 | 4.00 |
| 187.22 | 235.69 | 247.13 | 5.00 | 4.00 |
| 211.11 | 273.48 | 260.57 | 5.00 | 4.00 |
